# MIF promotes cell invasion by the LRP1-uPAR interaction in pancreatic cancer cells

**DOI:** 10.3389/fonc.2022.1028070

**Published:** 2023-01-10

**Authors:** Huizhi Sun, Runfen Cheng, Danfang Zhang, Yuhong Guo, Fan Li, Yanlei Li, Yue Li, Xiaoyu Bai, Jing Mo, Chongbiao Huang

**Affiliations:** ^1^ Tianjin Medical University Cancer Institute and Hospital, National Clinical Research Center for Cancer, Key Laboratory of Cancer Prevention and Therapy, Tianjin, China; ^2^ Department of Pathology, Tianjin Medical University, Tianjin, China

**Keywords:** MIF, uPAR, LRP1, hypoxia, spatial transcriptomics

## Abstract

**Introduction:**

Pancreatic ductal adenocarcinoma (PDAC) is characterized by high aggressiveness and a hypoxic tumour microenvironment. Macrophage migration inhibitory factor (MIF) is a hypoxia-related pleiotropic cytokine that plays important roles in cancer. However, its role in PDAC progression has not been fully elucidated.

**Methods:**

The clinical significance of MIF and hypoxia inducible factor 1 subunit alpha (HIF1A) in PDAC was analysed using immunohistochemical staining on PDAC tissues and data from KM-Plotter database. Spatial distribution of MIF and HIF1A gene expression was visualized by spatial transcriptomics in PDAC cell xenografts. To monitor the role of MIF in PDAC cell malignancy, immunostaining, lentivirus shRNA, migration assays, flow cytometry, transcriptomics and in vivo tumorigenicity were performed.

**Results:**

The spatial distribution of MIF and HIF1A was highly correlated and that high MIF expression was associated with poor prognosis of PDAC patients. MIF knockdown impaired cell invasion, with a decrease in the expression of urokinase-type plasminogen activator receptor (uPAR). Although PLAUR transcript was not reduced, a uPAR endocytic receptor, low-density lipoprotein receptor–related protein 1 (LRP1), was upregulated at both the mRNA and protein levels after MIF knockdown. The LRP1 antagonist RAP restored uPAR expression and invasiveness. MIF attenuated the nuclear translocation of p53, a transcriptional regulator of LRP1. Furthermore, MIF downregulation blunted the growth of PDAC cell xenografts and inhibited cell proliferation under normoxia and hypoxia. Transcriptome analysis also provided evidence for the role of MIF in cancer-associated pathways.

**Discussion:**

We demonstrate a novel link between the two pro-invasive agents MIF and uPAR and explain how MIF increases PDAC cell invasion capability. This finding provides a basis for therapeutic intervention of MIF in PDAC progression.

## 1 Introduction

Pancreatic ductal adenocarcinoma (PDAC), the most common type of pancreatic cancer, contributes to a five-year survival rate of only 10% (1). Due to the characteristics of invasive growth, early metastasis and general resistance to chemotherapy or radiotherapy, a potentially curative treatment is surgical resection for PDAC diagnosed early enough (2). The hypoxic tumour microenvironment of PDAC contributes to aggressive tumour biology. Hypoxia is involved in tumour cell genomic instability, immune responses, metabolic reprogramming, cancer stem cells, etc., through effects on levels of mRNA, protein expression and DNA methylation (3). Understanding the mechanisms responsible for the highly aggressive and metastatic characteristics of PDAC is required to improve treatment and disease outcomes. Under hypoxia, significant alterations in the mRNA expression of genes involved in invasion and metastasis were observed across different cancer types, including macrophage migration inhibitory factor (MIF).

MIF is a pluripotent and pleiotropic cytokine and is broadly expressed in various cell types and tissues. MIF plays roles in a multitude of disorders, such as immune abnormalities, heart disease, neurodegeneration, and the development of malignancies (4, 5). MIF is localized extracellularly and intracellularly, interacting with various cell surface and intracellular proteins, so it functions in an autocrine and paracrine way (6–9). Extracellular MIF binds to receptor complexes, and intracellular MIF modifies effector functions, leading to specific phenotypes (10, 11). MIF overexpression is frequently observed in most cancers and is a poor prognostic indicator (12). Under hypoxic conditions, the stabilization of hypoxia-inducible factor 1alpha (HIF-1a) promotes MIF expression (13), and MIF prevents the degradation of HIF-1a (14, 15), showing a collaboration between HIF-1a and MIF in MIF-associated pro/antitumour effects. Because of the multifaceted and critical functions of MIF, it has emerged as a therapeutic target (16–18).

Tumor cell invasion is a complex and multistep process, involving remodeling of the extracellular matrix (ECM) by tumor cell-associated proteases, which lead to proteolysis and dispersion of the obstacles and barriers. Of note is that MIF has emerged as an inducer of cancer cell migration and invasion by up-regulating and activating matrix metalloproteases (MMPs), such as MMP2, MMP9 and MMP13, then contributed to degradation of ECM (19–24). ECM restructure induced by the plasmin, serine protease urokinase plasminogen activator (uPA), uPA receptor (uPAR) and multiple MMPs has been well documented (25–28). The uPAR-uPA system is important for MMP2 and MMP9 activation (27, 29). The uPA has a wide range of targets, and its activation needs the binding of pro-uPA to uPAR (30). Overexpression of uPAR is significantly associated with poor prognosis in PDAC patients (31). uPAR is a membrane-anchored protein and binds to its high-affinity ligand uPA to facilitate the activation of proteases and cell signalling pathways (32, 33). Furthermore, uPA decreases the uPAR protein half-life by interacting with a multiligand endocytic receptor, low-density lipoprotein receptor–related protein 1 (LRP1). As a member of the low-density lipoprotein receptor family, which functions in receptor-mediated uptake of extracellular molecules, LRP1 is reported to regulate the abundance of proteins in the plasma membrane, including uPAR (34). By regulating cell signalling and gene expression, LRP1 is also reported to be associated with tumorigenesis and tumour progression (35).

In this study, we investigated the relationship of MIF expression with hypoxia status, cell invasiveness and MIF-related transcriptional changes in PDAC cells. Interestingly, we observed that the MIF-mediated reduction in cell invasiveness is regulated by the LRP1-uPAR interaction through the mechanism of MIF-induced inhibition of p53 and p53-induced LRP1 expression. We described a newly recognized regulatory mechanism of MIF in cell invasion, indicating potential implications for MIF-targeting therapeutic strategies.

## 2 Materials and methods

### 2.1 Clinical patient samples

Eighty-four pancreatic cancer specimens were obtained with informed consent from patients according to protocols approved by the Tianjin University Cancer Hospital Ethics Committee. Patients were diagnosed with pancreatic duct adenocarcinoma by postoperative pathology, and axillary node metastases were present in 13 patients. The median age of the patients was 59 years (range 41–72 years). The follow-up period began at the time of surgery and ended in June 2016. The collected sample tissues were fixed with neutral buffered formalin and embedded in paraffin for subsequent experiments.

### 2.2 Histopathology, immunohistochemistry and immunofluorescence

Haematoxylin and eosin staining (H&E) and immunohistochemical analysis were performed on pancreatic cancer and xenograft tissues following standard protocols to analyse the tumour histology and the following proteins: MIF (Abcam, ab65869) and Ki-67 (Abcam, ab279653). Immunohistochemical staining was scored as previously published (36). For immunofluorescence, adherent cells were grown on coverslips, fixed in 4% paraformaldehyde for 15 min, and stained with the following antibodies: uPAR (Abcam, ab103791) and p53 (Abcam, ab16665). Alexa Fluor 568 (Invitrogen, A10437) was used as the secondary antibody. Cell nuclei were counterstained with 4’, 6-Diamidino -2-phenylindole dihydrochloride (DAPI), and images were recorded on a Nikon A1R-A1 confocal microscope (Nikon Corporation, Tokyo, Japan).

### 2.3 Clinical significance of MIF and gene expression analysis

The prognostic values of MIF and HIF1A mRNA expression were evaluated using the KM-Plotter database (http://kmplot.com/analysis/). Progression-free survival (PFS) and overall survival (OS) analyses were performed in 177 PDAC patient samples according to the median mRNA expression. We also explored the prognostic values of MIF in TCGA-pancreatic cancer (PAAD) and the relationship between the expression levels of MIF and LRP1 using UCSC Xena (xena.ucsc.edu/) (37).

### 2.4 Spatial transcriptomics

Pancreatic cancer cell line PANC1-derived xenografts were collected as our previous study (38). Briefly, pancreatic cancer cells were subcutaneously transplanted into the groins of nude mice after left hind limb ischaemia by femoral artery ligation or sham operation (n=4 for each group). Frozen sections from the xenografts were analysed by 10x Genomics and spatial transcriptomics sequencing following the manufacturer’s procedure (Supplementary materials: CG000238_Visium Spatial Tissue Optimization User Guide, CG000239_Visium Spatial Gene Expression_User Guide_Rev_A, CG000240_Visium Spatial Protocols_Tissue Preparation Guide_Rev A and SAM000092_Planner_Visium Spatial Gene Expression Workflow Planner_Rev A).

### 2.5 Cell culture

The human pancreatic cancer cell lines PANC-1, SW1990, AsPC-1, BxPC-3 and CFPAC-1 were purchased from the Type Culture Collection Committee of the Chinese Academy of Sciences. Cells were maintained in Roswell Park Memorial Institute 1640 Medium (RPMI 1640), Dulbecco’s Modified Eagle Medium (DMEM), or Iscove’s Modified Dulbecco’s Medium (IMDM) supplemented with 10% FBS and 1% penicillin/streptomycin at 37°C with 5% CO_2_ in a humidified incubator. Cobalt chloride (CoCl_2_)-treated cells were established as an *in vitro* hypoxia-mimetic condition. SW1990, BxPC-3, AsPC-1 and PANC-1cells were treated with 150 μM, 200 μM, 150 μM and 300 μM CoCl_2_ respectively for 24 h. To inhibit LRP1 function, pancreatic cancer cells were treated with the LRP1 antagonist receptor-associated protein (RAP) (0.5 μM) (human recombinant RAP, 553506 Sigma−Aldrich) for 3 days.

### 2.6 Lentivirus production and transduction

The lentivirus expressing shRNA targeting MIF (NM_002415) (targeting 5’-GACAGGGTCTACATCAACTAT-3’) and lentivirus vector for full-length MIF overexpression (NM_002415) were synthesized by GeneChem (Shanghai, China). AsPC-1 and PANC-1 cells were transduced with shRNA targeting MIF (shMIF) or a nontargeting control (shNTC: 5’-TTCTCCGAACGTGTCACGT-3’); SW1990 and BxPC-3 cells were transduced with MIF overexpression lentivirus (exMIF) or a control vector (vec). Stably transduced cell lines were generated by lentivirus infection for 12 h, followed by selection with puromycin (0.5 mg/ml) for 2 weeks.

### 2.7 Western blot analysis

For immunoblot analysis, cells were washed twice with PBS, harvested and lysed with RIPA buffer. Whole-cell proteins were separated by SDS−PAGE, transferred onto polyvinylidene fluoride (PVDF) membranes and immunoblotted with antibodies against MIF (Abcam, ab65869), uPAR (Abcam, ab103791), LRP1 (Abcam, ab92544) and GAPDH (Santa Cruz Biotechnology, sc25778). Blots were detected with WesternBright ECL HRP substrate (R-03031-D2, advansta) and visualized with a C-DiGit Blot Scanner (LI-COR Biosciences).

### 2.8 Cell migration

Cell invasion and migration were examined using transwell assays and scratch wound healing assays as previously described (39). For transwell invasion/migration assays, 2x10^4^ cells were plated in the upper chamber (Transwell chambers, 8 mm pore size, BD Biosciences) in serum-free culture medium with or without Matrigel matrix (BD Biosciences) on the inserts. In the lower chamber, 10% FBS was used as the chemoattractant. After 24 hours, migratory cells on the lower membrane surface were stained with crystal violet and counted for three random 100x fields per well. For wound healing assays, cells were seeded in 12-well plates. Confluent cell monolayers were scraped with 200 µl pipette tips and washed twice with PBS. Wound healing images were captured at 0, 24 and 48 hours after scratching. Cell motility was assessed by measuring the distance from each side of the cell wound. Images were documented under a phase contrast microscope.

### 2.9 Real-time reverse transcription PCR

Total RNA isolation from pancreatic cancer cells was performed using the SPARKeasy Cell RNA Kit (Sparkjade, AC1601) followed by reverse transcription using the All-in-one First-Strand cDNA Synthesis Kit (GeneCopoeia, AORT-0020). Synthesized cDNA was subjected to SYBR Green real-time PCR (All-in-one qPCR Mix, GeneCopoeia, AORT-0600) using the following primers: LRP1 forward 5′-CTGGCGAACAAACACACTGGa-3″, LRP1 reverse 5′-CACGGTCCGGTTGTAGTTGA-3′; PLAUR forward 5′-GAGAGAAGACGTGCAGGGAC-3′. PLAUR reverse 5′-ACTCTTCCACACGGCAATCC-3′. The relative LRP1 or PLAUR mRNA expression levels were normalized to GAPDH expression. Each synthesis and amplification were run in triplicate for the target and internal control genes.

### 2.10 Flow cytometry

Apoptotic cells were quantified using an Annexin V-APC/propidium iodide (PI) apoptosis double staining kit (US EVERBRIGT, A6030) according to the manufacturer’s instructions. Briefly, cells were harvested using trypsin, centrifuged at 2,000 rpm for 5 min, and stained with 5 μl Annexin V-APC and 5 μl PI for 15 min in the dark. Untreated cells were used as the negative controls. Data analysis was performed using a BD AccuriR C6. Annexin V-positive and PI-negative cells were identified as early apoptotic cells, and Annexin V-positive and PI-positive cells were identified as late apoptotic cells. The extent of apoptosis was calculated as the percentage of both cell populations.

### 2.11 Mouse xenograft assays

Female BALB/c-nu mice (4-6 weeks of age) were obtained from Beijing HFK Biosciences (Beijing, China). Animal studies were performed according to protocols approved by the Institutional Animal Care and Use Committee at Tianjin Medical University. Pancreatic cancer cell suspensions (5x10^6^) were prepared for subcutaneous injections into the groin after left hind limb ischaemia by femoral artery ligation or sham operation (40). Tumours were measured using a calliper every second day until harvest at 3-4 weeks, and tumour volumes (TV) were determined using the formula (TV (mm^3^) = 0.5 × length × (width)^2^).

### 2.12 Transcriptomics

Total RNAs of PANC-1 cells transduced with shMIF or shNTC were sent to LC-Bio Technology (Hangzhou, China) for sequencing and analysis. After RNA purification, the 2×150bp paired-end sequencing (PE150) on an Illumina Novaseq™ 6000 was performed. Reads of all samples were aligned to the homo sapiens reference genome using HISAT2 (https://daehwankimlab.github.io/hisat2/, version: hisat2-2.2.1) package. Genes differential expression analysis was performed by DESeq2 software between two different groups. The genes with the parameter of false discovery rate (FDR) below 0.005 and absolute fold change ≥ 2 were considered differentially expressed genes (DEGs). DEGs were then subjected to Gene ontology (GO) enrichment and Kyoto Encyclopedia of Genes and Genomes (KEGG) analysis performed by Lc-bio (https://www.lc-bio.cn/). Details are provided in the Supplementary materials.

### 2.13 Statistical analysis

GraphPad Prism 8.0 (GraphPad Software) was adopted for statistical analysis. All experiments were performed with at least three replicates per condition. Measurement data are reported as the mean ± standard deviation (SD). For comparisons between more than two groups, analysis of variance (ANOVA) followed by *post hoc* Tukey’s (for multiple comparisons) test was used. For comparisons between two groups, Student t test was performed. For data of abnormal distribution, the nonparametric Kruskal-Wallis or Mann Whitney-U test was applied. Survival time was analysed using the Kaplan–Meier method and compared by the log-rank test. For all statistical tests, P ≤0.05 was considered significant.

## 3 Results

### 3.1 HIF1A and MIF expression is associated with worse outcomes in PDAC patients

**Figure 1:**
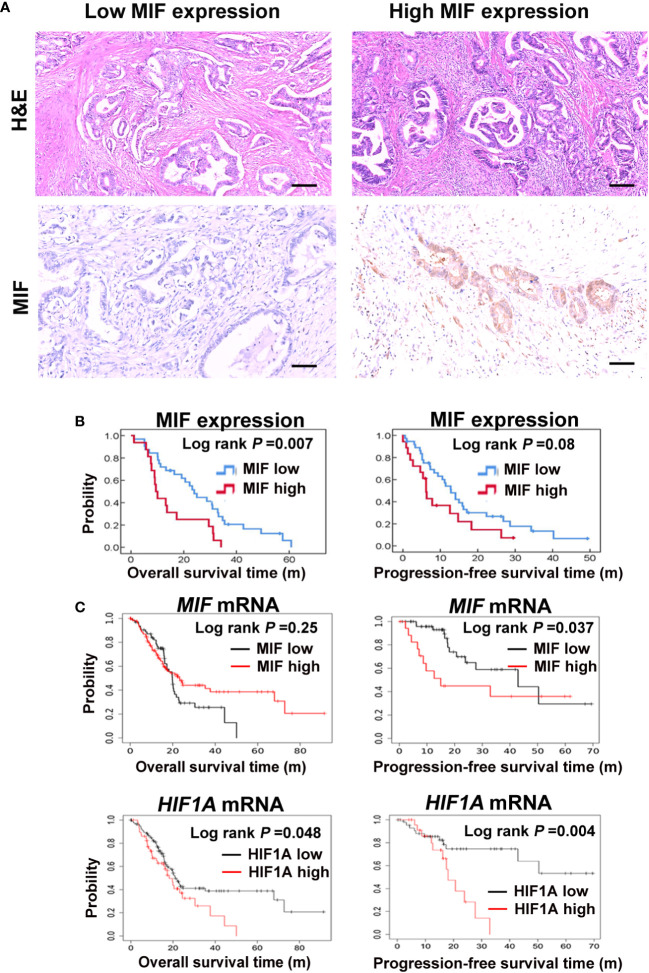
Elevated MIF expression correlates with a poor prognosis in PDAC patients **(A)** Pathological and histological assessment of 84 human PDAC sections, including haematoxylin eosin staining (H&E) and expression of MIF by immunohistochemistry staining. Representative images of MIF immunostaining with low and high intensity. Scale bars, 100 μm. **(B)** Kaplan−Meier plots show the correlations between MIF mRNA expression and the OS/PFS of 84 PDAC patients. **(C)** Survival curves show the relationships between MIF or HIF1A mRNA expression and OS/PFS of 177 PDAC patients from the KM-Plotter database.

High MIF levels are associated with a poor prognosis in multiple cancer types through various mechanisms (41–45). To test the above point, we investigated the involvement of MIF in the outcomes of PDAC patients. Eighty-four patients with PDAC who underwent surgical resection were included in the study. The pathological characteristics of these PDAC tissues were detected by H&E staining. Levels of MIF expression were examined using immunohistochemical techniques, and staining scores were graded as weakly positive (5-49%) and strongly positive (>50%) based on the percentage of positively stained tumour cells (**Figure 1A**). Fifty-seven PDAC patients showing negative or weak tumour MIF expression had a better OS (*P =* 0.007) and a trend for increased PFS (*P =* 0.08) compared with the 27 patients whose tumour showed strong MIF expression using Kaplan−Meier analyses (**Figure 1B**). The clinical significance of MIF and HIF1A mRNA was also analysed in 177 PDAC samples from the KM-Plotter database. Although MIF mRNA expression and OS did not show a correlation (*P =* 0.25), it was associated with poor PFS (*P =* 0.037). The mRNA expression of HIF1A was significantly related to both the OS and PFS of PDAC patients (P = 0.048; P = 0.004). (**Figure 1C**). Although high MIF expression may predict a worse prognosis, no difference of MIF expression was observed in TNM (Tumor, Nodal Involvement, Metastasis) stages of 196 TCGA pancreatic cancers using UCSC Xena database (xena.ucsc.edu/) (*P =* 0.7862, **Figure S1**). These results suggest that high levels of the hypoxia-responsive factor MIF might be a predictor of worse survival of PDAC patients, which is consistent with the study by Ahmed A et al., highlighting the role of MIF as a biomarker candidate for the diagnosis and prognosis of PDAC (44).

### 3.2 Visualization of MIF and HIF1A gene expression in PDAC xenografts by spatial transcriptomics

**Figure 2:**
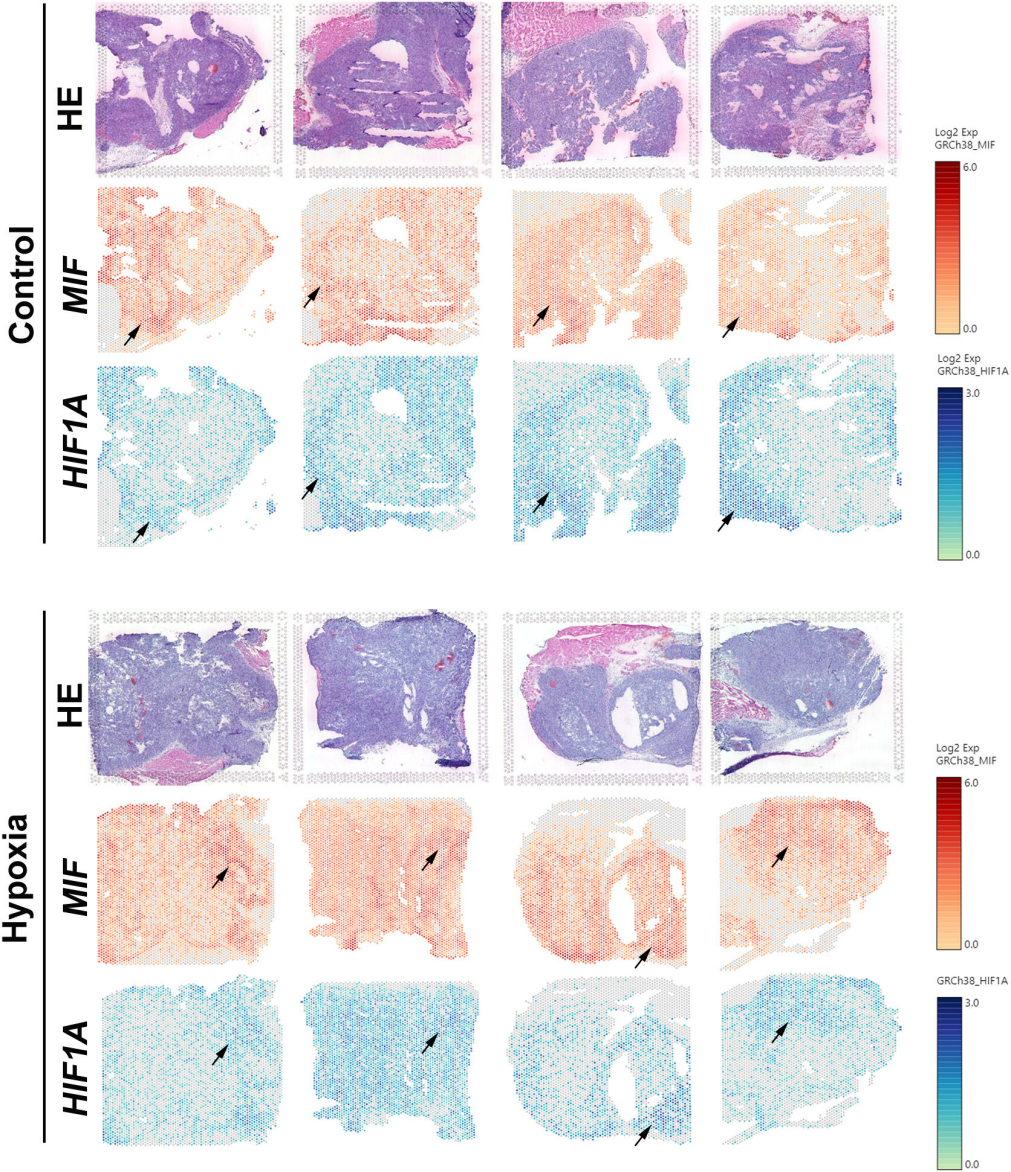
Visualization of the expression patterns of HIF1A and MIF genes in PDAC xenografts by spatial transcriptomics Spatially resolved expression of HIF1A and MIF in tumour sections taken from 8 PDAC xenografts subcutaneously in the mouse groin after femoral artery ligation of the left hind limb (hypoxia) or sham operation (control).

Since hypoxia-induced expression and secretion of MIF has been observed before (13, 46), we next investigated the expression and spatial distributions of the hypoxia marker genes HIF1A and MIF in PDAC xenografts by spatial transcriptomics as we reported previously (38). For this, PANC-1 cells were subcutaneously injected into the mouse groin after femoral artery ligation of the left hind limb or sham operation. By 10x Genomics and spatial transcriptomics sequencing, we investigated the gene expression pattern of HIF1A and MIF in 8 sections from PDAC cell-derived xenografts. The spatially resolved expression profile and position of the HIF1A and MIF genes showed approximately the same levels and overlapping positions (**Figure 2**). These data display positional information on the MIF gene related to the hypoxia marker HIF1A, validating that hypoxia is a potent inducer of MIF expression in PDAC.

### 3.3 MIF is generally expressed in pancreatic cancer cell lines

**Figure 3:**
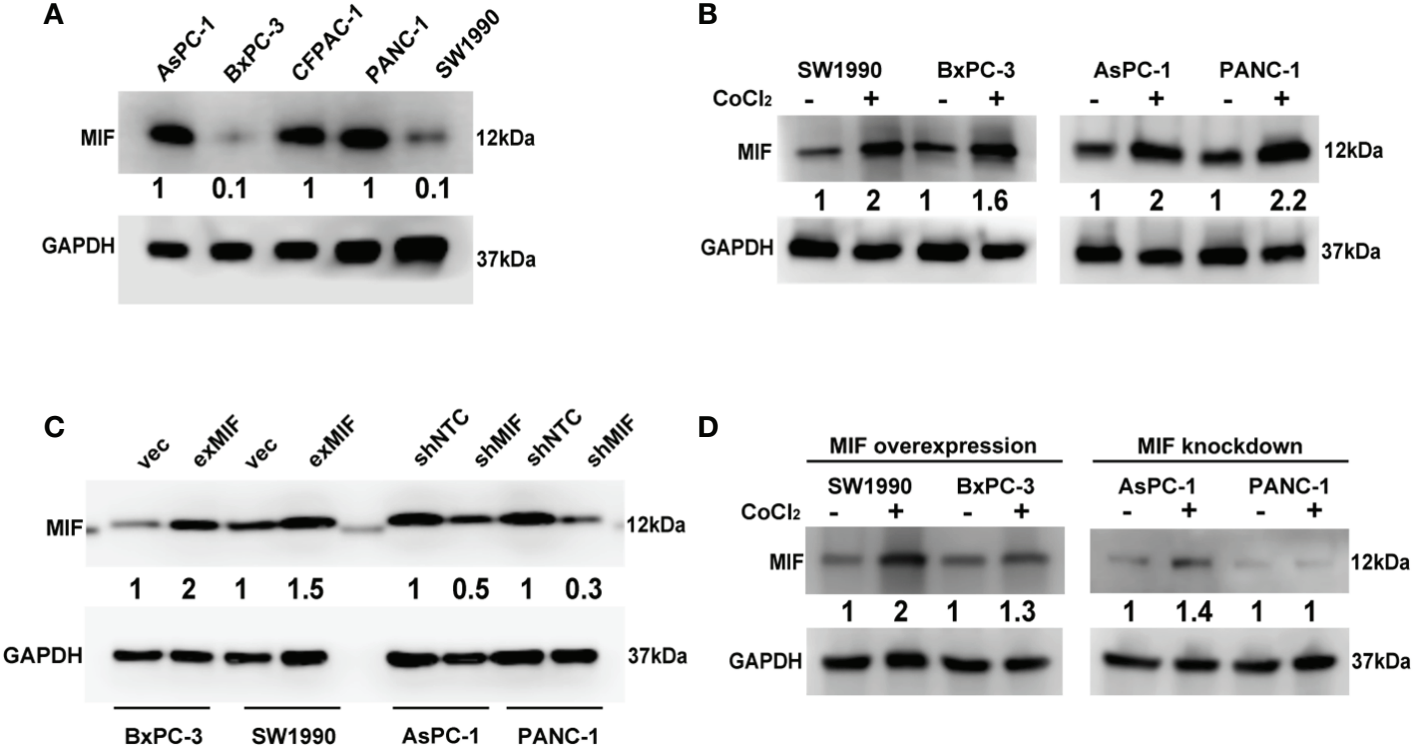
Detection of MIF expression in PDAC cell lines Western blot analysis of MIF protein levels in five human PDAC cell lines **(A)**, MIF expression after CoCl_2_ treatment for 24 h compared with vehicle **(B)**, transduction efficiency of lentivirus-cDNA-MIF (exMIF) or vector (vec) for MIF overexpression and lentivirus-shRNA (shMIF) or nontarget control (shNTC) for MIF knockdown **(C)**, and MIF expression after CoCl_2_ treatment in stably transduced cells **(D)**. The loading control was assessed by GAPDH blotting. The numbers below the blots are quantitative ratios of the MIF/GAPDH band densities. CoCl_2_ concentration used: SW1990, 150 μM; BxPC-3, 200 μM; AsPC-1, 150 μM; PANC-1, 300 μM.

To detect the expression of MIF in most used pancreatic cancer cell lines and its relationship with hypoxia *in vitro*, then we examined MIF protein expression in a panel of pancreatic cancer cell lines using western blotting. The results showed that MIF was generally and differentially expressed in these five pancreatic cancer cell lines (**Figure 3A**). Since the MIF promoter contains responsive elements for HIF-1a (47), we then determined whether there was a correlation between hypoxic conditions and MIF expression *in vitro*. For hypoxia induction, cells were cultured with a hypoxia-mimetic agent, CoCl_2_ at the indicated concentration for 24 h. The changes in MIF protein levels examined by western blotting showed that CoCl_2_ treatment markedly elevated MIF protein levels. (**Figure 3B**).

To determine the effect of MIF on the malignant phenotype of pancreatic cancer cells, we started to construct MIF-overexpressing and MIF-knockdown cells according to the basal expression levels of MIF, as shown in **Figure 3A**. SW1990 and BxPC-3 cells were selected for stable transduction with lentivirus-MIF-cDNA (exMIF) or control vector (vec); PANC-1 and AsPC-1 cells were selected for stable transduction with MIF-shRNA (shMIF) or nontarget control shRNA (shNTC). The up/down regulation efficiency of MIF expression was quantified by western blot. As shown in **Figure 3C**, MIF protein was elevated in both SW1990 and BxPC-3 cells compared with the control vector; MIF-shRNA dramatically reduced MIF protein in both PANC-1 and AsPC-1 cells compared with the nontarget control. In addition, CoCl_2_ treatment markedly elevated the MIF protein levels in MIF-overexpressing cells, whereas in MIF knockdown cells, no significant increase in MIF expression was observed (**Figure 3D**). These data show that MIF is generally expressed in pancreatic cancer cell lines and can be enhanced by the hypoxia-mimicking agent CoCl_2_ *in vitro*.

### 3.4 MIF promotes the invasion of pancreatic cancer cells

**Figure 4:**
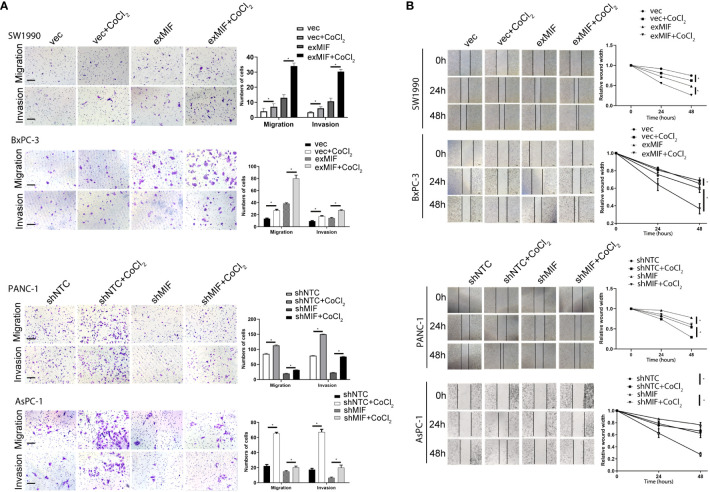
MIF promotes the invasion/migration of PDAC cells *in vitro* **(A)** Transwell migration and invasion assay. Images of migration and invasion of each cell group were presented. The migrating cells were stained with violet color. The bar charts showing the average migration and invasion cell number per field among different experimental groups. Data are the mean ± SD from three independent experiments, *P<0.05. Scale bars, 100 µm. **(B)** Wound healing assay. Representative images showing the scratch at time 0 h, 24 h and 48 h of PDAC cells in indicated conditions. The line charts showing the quantitative analysis of the relative wound width. The values were normalized by the wound width at the same area of the scratch at time 0 h. *P < 0.05.

Increasing data indicated that MIF promoted migration and invasion of cancer cells, so to investigate the effects of MIF on the invasive phenotype of pancreatic cancer cells, we sought to determine the impact of altered expression of MIF on cellular mobility. We observed that overexpression of MIF promoted the migration and invasion of SW1990 and BxPC-3 cells, whereas knockdown of MIF attenuated the migration and invasion of PANC-1 and AsPC-1 cells (**Figure 4A, B**). These findings provide evidence that MIF has a significant role as a positive regulator of PDAC cell mobility.

### 3.5 MIF knockdown inhibits uPAR expression *via* LRP1-mediated internalization

**Figure 5:**
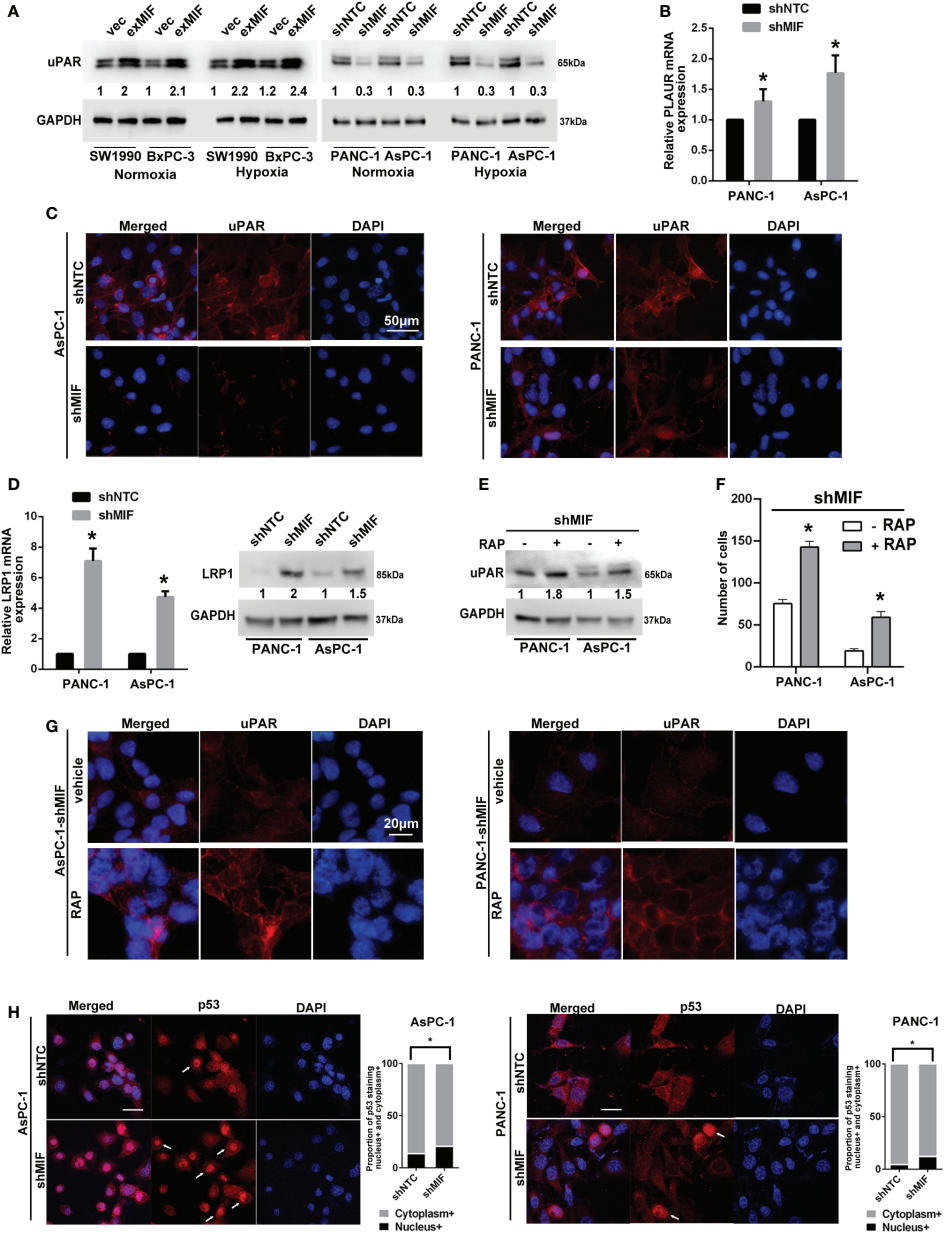
MIF enhances the expression of uPAR *via* LRP1 **(A)** Western blot analysis of uPAR in stably transduced PDAC cell lines after treatment with CoCl_2_ or vehicle for 24 h. The numbers below the blots are quantitative ratios of the uPAR/GAPDH band densities. **(B)** Real-time PCR analysis using specific primers for PLAUR with pancreatic cancer cell lines. **(C)** Confocal microscopy images of shMIF cells for uPAR expression on the cell surface and in the cytoplasm (red). Scale bars, 50 μm. **(D)** Real-time PCR and western blot analysis of the mRNA and protein levels of LRP1 in shMIF cells. **(E)** uPAR levels in shMIF cells treated with the LRP1 antagonist RAP or vehicle. **(F)** Quantification of invasion assays of shMIF cells treated with LRP1 antagonist, RAP or vehicle using Matrigel matrix-coated transwell chambers. **(G)** Immunofluorescence images of uPAR on the cell surface and in the cytoplasm with the addition of RAP or vehicle in shMIF cells. **(H)** Immunofluorescence images and quantification of nuclear/cytoplasmic p53 staining in AsPC-1 and PANC-1 cells. The graphs show the mean of the proportions of p53-positive staining in the nucleus versus the cytoplasm. Scale bars, 20 μm. *P < 0.05.

Cellular invasion/migration is important during tumour progression, and it requires the coordination of various biological events. To move, cells have to use proteinases to break down barriers of the surrounding matrix. Numerous studies demonstrated that MIF has been involved in the migration and invasion of cancer cells by up-regulating the pro-metastatic mediators MMPs, among which MMP2 and MMP9 degrades the ECM and facilitates the tumor cell invasion and metastasis (19, 23, 48, 49). In view of these results, we investigated the influence of MIF silencing and overexpressing on the levels of MMP2 and MMP9 using western blotting assay. Different from other researches, however, no changes of MMP2 or MMP9 expression were detected (data not shown). In consideration of the role of uPA/uPAR/plasmin system-mediated MMPs activation in tumour migration and invasion, we attempted to investigate the influence of MIF silencing and overexpressing on the levels of uPAR, a plasma membrane GPI-anchored protein, that binds with high-affinity and activates the serine protease uPA, thus regulating MMPs activity. Among them, uPAR has been implicated in the invasion and metastasis of tumours and correlated with a poor prognosis in several types of tumours. As shown in **Figure 5A**, the levels of uPAR were reduced in shMIF cells and elevated in exMIF cells. The downregulation of uPAR protein was more obvious in shMIF cells, and subsequent experiments *in vitro* were performed only in shMIF cells.

To determine the molecular basis for the effect of downregulated MIF expression on the levels of uPAR, we sought to detect the mRNA expression of uPAR in shMIF cells using real-time PCR. We found that the levels of uPAR mRNA were slightly elevated in shMIF cells (**Figure 5B**). As a multifunctional receptor, it is known that uPAR is internalized with uPA/PAI-1 (plasminogen activator inhibitor type-1) complexes *via* a mechanism involving LRP1 (34). Unligated uPAR is recycled back to the cell surface; however, uPAR recycling is not 100% efficient. As a result, the cell-surface abundance of uPAR can be reduced by LRP1-mediated uPAR endocytosis (50). To test whether the decreased expression of uPAR in shMIF cells is attributed to uPAR internalization, we detected the location of uPAR by immunofluorescence staining. Cell membrane and cytoplasmic uPAR were visualized by confocal microscopy imaging, which showed a moderate reduction in surface uPAR fluorescence intensity in shMIF cells compared with shNTC cells (**Figure 5C**).

To assess the possibility of the regulation of uPAR expression by LRP1, we detected the mRNA and protein levels of LRP1, which were elevated in shMIF cells (**Figure 5D**). Similarly, expression levels of MIF and LRP1 showed significantly negative correlation in 196 pancreatic cancers from TCGA using UCSC Xena database (Pearson’s rho r = -0.3718, *P =* 2.204e-7, **Figure S2**). This analysis suggested a significant negative correlation between LRP1 and MIF. To determine whether LRP1 participates in uPAR internalization, we measured the expression of uPAR with the LRP1 antagonist RAP (51, 52). We observed increased expression of uPAR in response to RAP in shMIF cells (**Figure 5E**). Importantly, as shown in **Figures 5F, G**, RAP treatment was sufficient to increase the number of invasive cells, and the signal intensity of cell surface uPAR was restored with decreased cytoplasmic uPAR in shMIF cells, suggesting that uPAR expression restoration by LRP1 inhibition leads to rescue of cell mobility. Finally, owing to previous studies suggesting p53-induced LRP1 upregulation and MIF-mediated inhibition of p53 activity (53–55), we reasoned that the increased LRP1 expression in shMIF cells may contribute to the release of p53 suppression by MIF knockdown. As expected, we observed a moderate increase in nuclear p53 fluorescence in MIF knockdown cells (**Figure 5H**) compared to shNTC cells, which corresponds with the effect of MIF on p53 nuclear import (56, 57). These results suggest one possible mechanism by which MIF promotes cell invasion by suppressing p53-regulated LRP1 expression and LRP1-uPAR endocytosis.

### 3.6 MIF and tumour development *in vivo*

MIF has been reported to be involved in the development and progression of a variety of tumours (58), and we next addressed the role of MIF in PDAC progression *in vivo*. We established subcutaneous xenografts in nude mice using MIF knockdown, MIF-overexpressing and control cells.

**Figure 6:**
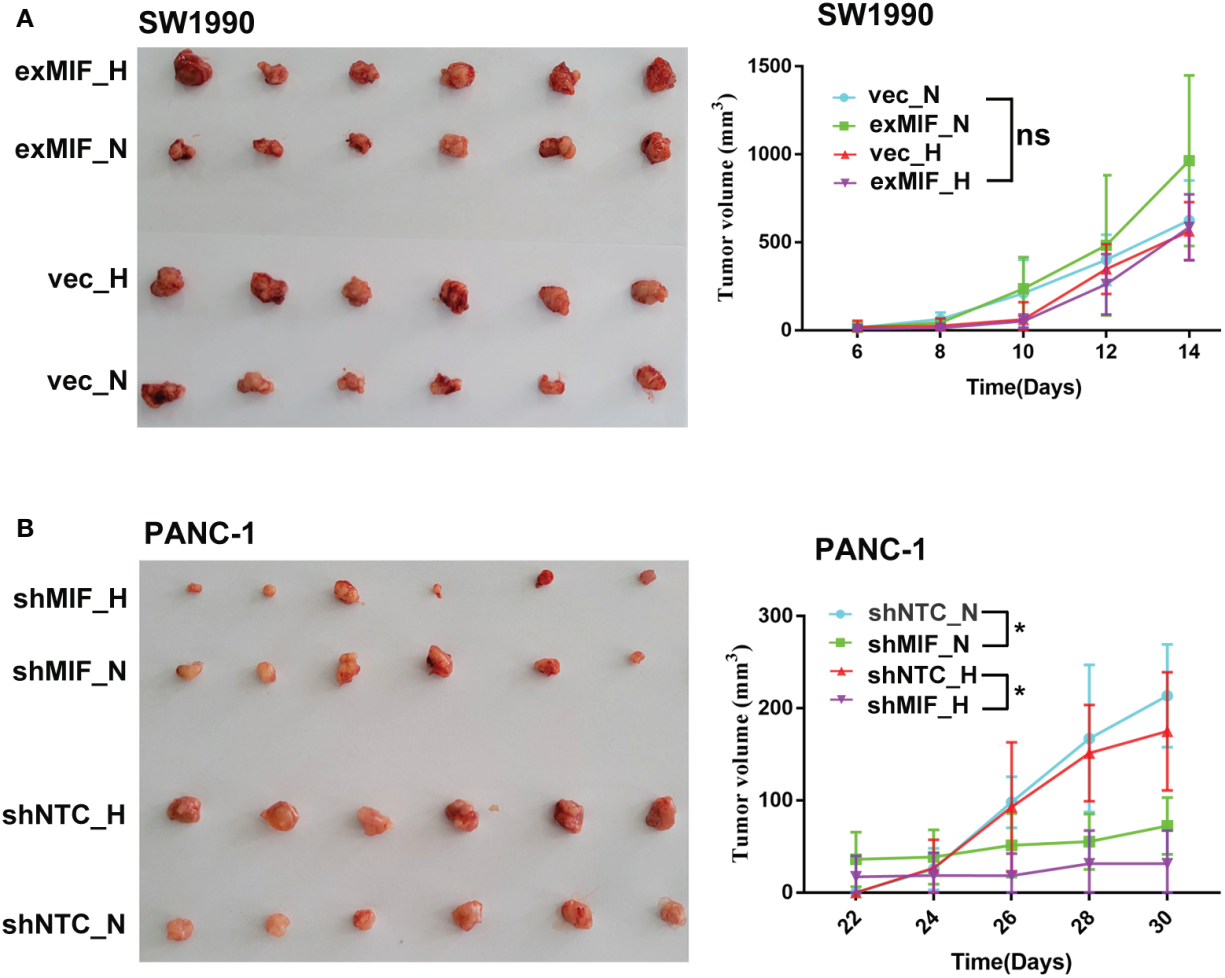
MIF knockdown attenuates the tumorigenesis of PDAC cells *in vivo* Representative images and quantification of subcutaneous xenograft tumours (n = 6 in each group) from stably transduced cell lines SW1990 **(A)** and PANC-1 **(B)** in nude mice after sham operation (N, normoxia) or femoral artery ligation (H, hypoxia). ns, no significance; *P < 0.05.

**Figure 7:**
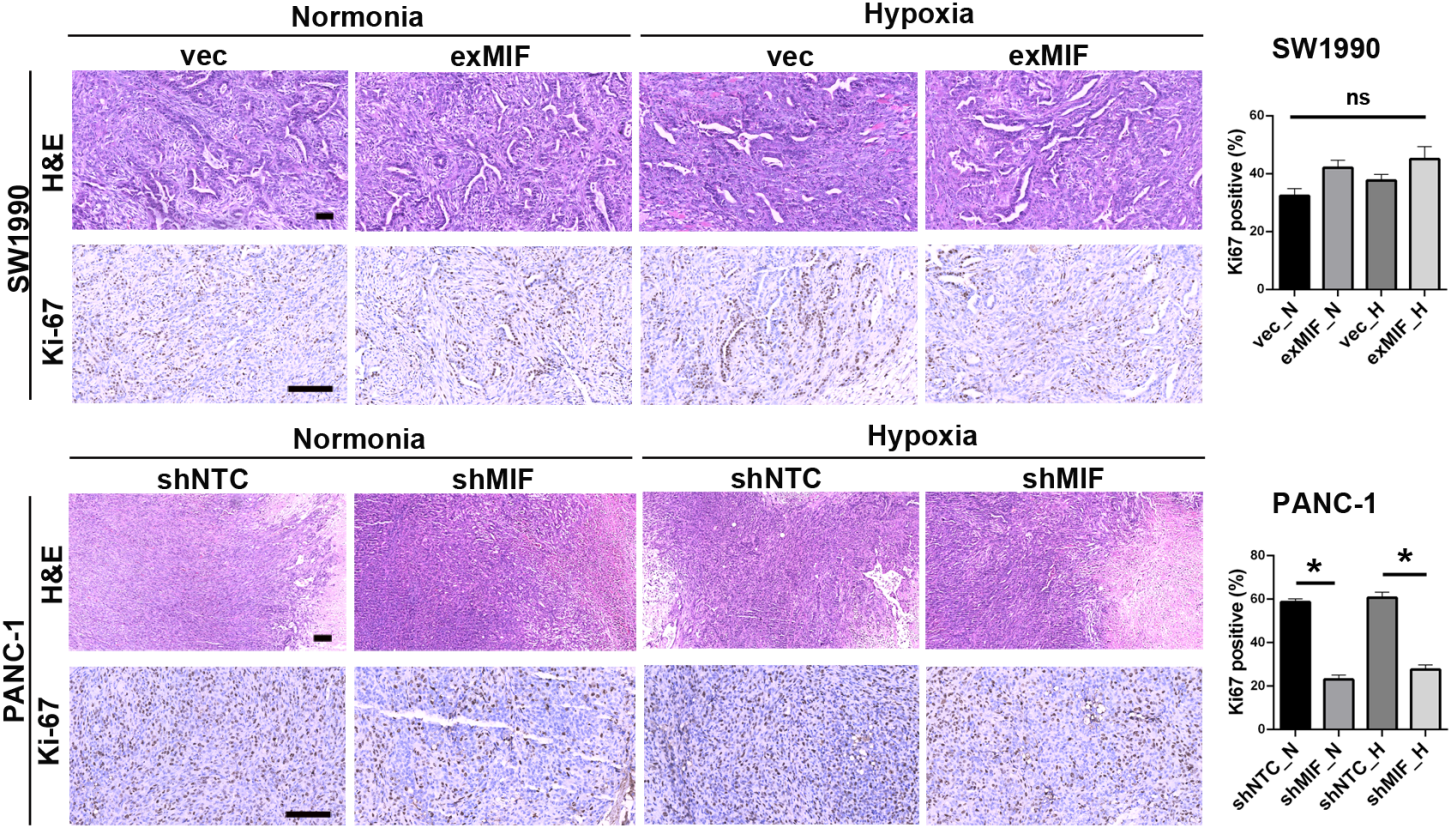
MIF knockdown inhibits proliferation of PDAC cells Representative images of xenograft tumours stained for H&E and Ki-67. The histograms show the quantification of Ki-67 staining; n = 6 per group. *P < 0.05.

The volume of tumours developed in mice injected with shNTC cells was significantly higher than that in mice receiving shMIF PANC-1 cells. However, there were no significant differences in tumour volumes between the vector control and exMIF SW1990 cell groups (**Figure 6**). In addition, there were differences in tumour growth between the normoxic (sham operation) and hypoxic (femoral artery ligation of hind limb) groups either in shMIF vs. shNTC or in exMIF vs. vector groups. The protumorigenic ability of MIF may partially result from its prosurvival and antiapoptotic effects. As shown in **Figure 7**, the expression of Ki-67, a proliferating cell marker, was quantitated on the basis of the percentage. Consistently, while we did not identify significant differences in Ki-67-positive staining in the exMIF and vector control SW1990 cell groups, a low percentage of Ki-67-positive staining was observed in shMIF PANC-1 cells under normoxia and hypoxia. Meanwhile, apoptosis was determined by Annexin V-APC/PI staining analysis using flow cytometry, which showed that the percentages of apoptotic cells were higher in the MIF knockdown groups than in the shNTC groups and fewer apoptotic cells in the MIF-overexpressing groups than in the vector groups (**Figure S3**). With regard to discrepant results between *in vivo* and *in vitro*, we speculate that this may be due to the complex society of the tumour microenvironment *in vivo*, disturbing the prosurvival role of MIF. Collectively, these results indicated that the MIF-involved suppression of tumour growth may be due to its anti-apoptosis role.

### 3.7 MIF knockdown triggers multiple tumour-associated processes in PDAC cells

**Figure 8:**
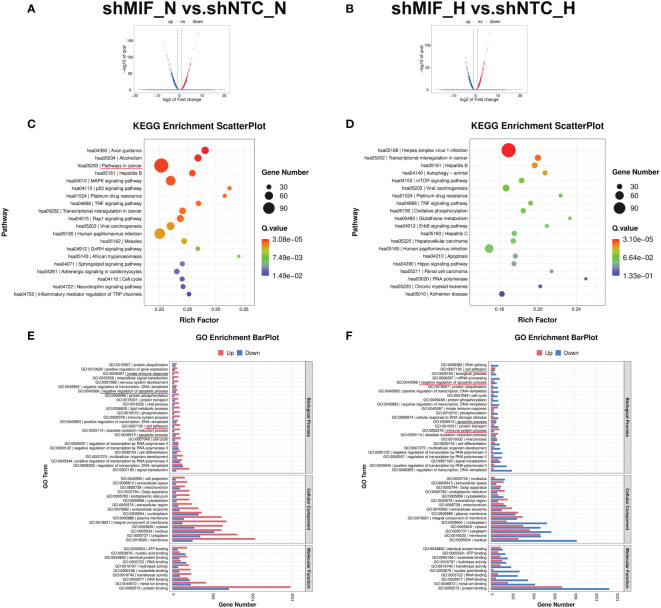
RNA-seq analysis in MIF knockdown PANC-1 cells **(A, B)** Volcano maps demonstrate the DEGs (fold-change >2 and FDR <0.005) regulated by MIF knockdown in PANC-1 cells. **(C, D)** KEGG pathway enrichment of the DEGs. **(E, F)** GO enrichment analysis of DEGs divided into three functional groups: biological process, cellular component, and molecular function.

To further explore the mechanisms underlying the MIF knockdown-induced cell invasive phenotype attenuation, we performed transcriptome RNA-seq analysis in MIF knockdown PANC-1 cells. After screening, 3438 and 2802 significantly DEGs of shMIF vs. shNTC PANC-1 cells in normoxia (2259 up, 1179 down) and hypoxia (985 up, 1817 down) were shown in the volcano figures (**Figures 8A, B**). Then, the DEGs were analysed for their functions and enrichment pathways by GO and KEGG pathway analysis. Several biological processes associated with cancer progression were enriched in shMIF cells, including pathways in cancer, transcriptional misregulation in cancer, innate immune response, regulation of apoptosis and cell adhesion (**Figure 8C-F**). These findings correspond with our *in vivo* analysis and provide more comprehensive explanations for the role of MIF in the malignant behaviour of PDAC cells.

## 4 Discussion

Tumour aggressiveness and treatment resistance are partially due to the complex milieu characterized by hypoxia, which is an important microenvironment feature in PDAC (38). The highly invasive and metastatic nature of pancreatic cancer requires the development of effective therapeutic interventions. Under hypoxia, multiple adaptive changes occur through hypoxic effects on the levels of mRNA, protein expression and DNA methylation. Buffa, F. M. et al. validated a common hypoxia signature in multiple cancers (46). By investigating hypoxia-induced spatial transcriptome distribution in human PDAC cells engrafted into mouse ischaemic hind limbs, we also identified multiple hypoxia-associated genes. Among them, a proinflammatory cytokine, MIF, attracted our attention.

It has been clear for the regulatory role for HIF-1α in the expression of MIF, and the role for MIF in HIF-1α stabilization (13–15). The reprogramming of cancer cells during hypoxic adaptation, in the respects of gene expression alterations, metabolism regulation, ECM deposition, remodeling and degradation, etc., has been extensively reviewed (59–62). In the research field of regulation of cancer cell invasion and metastasis, MIF showed similar effect or intersection with hypoxia, such as association with an increased expression of MMPs and PLAUR (19, 63–65) and macrophage recruitment (66, 67). Given the importance of MIF in malignant disease progression at hypoxia, in-depth studies of MIF directed therapeutics in cancer development are critical. MIF is ubiquitously and constitutively expressed in almost all cells, including numerous human cancer types, and it is involved in numerous biological processes (18). Emerging evidence has demonstrated the biological effects of MIF on the malignant characteristics of PDAC (68–72). Consistently, in the present study, we also observed the role of MIF in pro-invasiveness, anti-apoptosis and prognostic prediction. Interestingly, the main finding and focus of the current study was to investigate the involvement of LRP1-mediated uPAR endocytosis in the MIF-related invasive phenotype of PDAC.

uPAR has been shown to be upregulated in cancer cells, to play a critical role in driving plasminogen-dependent matrix degradation and to affect a variety of physiological and pathological processes, including cell migration, invasion, adhesion, survival and proliferation (73). In this report, we found a decreased protein abundance but not mRNA of uPAR in MIF-knockdown PDAC cells, suggesting that MIF may not affect PLAUR transcription. It is well known that internalization of cell uPAR, followed by either recycling back to the cell surface or degradation, is crucial for its homeostasis and functional signalling. Hence, to test the possibility that MIF altered the stability of uPAR, we examined its cellular location. Our data suggest that MIF knockdown regulated the cellular distribution of uPAR from the cytomembrane to the cytoplasm. The endocytosis of uPAR has been identified through LRP1, which is an endocytic receptor for multiple ligands. Next, we demonstrated the overexpression of LRP1 at both the mRNA and protein levels in MIF knockdown PDAC cells. Based on these results, we hypothesize that LRP1 is involved in the MIF-related decrease in the cell-surface abundance of uPAR. To investigate this possibility, we used the LRP1 antagonist RAP, which blocks the binding of soluble ligands to LRP1, subsequently blocking uPAR internalization. Our data showed that both the total and cell-surface abundance of uPAR protein were restored in the presence of RAP. Collectively, this evidence supports the role of LRP1 as a ubiquitous endocytic cell surface receptor that recognizes a wide range of ligands (34, 74).

Studies have indicated the role of LRP-1 in cancer progression. Although several studies have reported that a low level of LRP1 was associated with the aggressive phenotype of certain types of cancer (75–77), other studies have demonstrated that upregulation of LRP1 expression was observed in the progression of certain types of malignancy (78, 79). These results suggested that LRP1 expression may be dependent on the cancer subtypes and stages. Understanding how LRP1 is regulated is important for unscrambling its pleiotropic role. Data about the basal molecular mechanisms of LRP1 regulation have not been clearly characterized. A review by H. Emonard et al. described the regulation of LRP1 expression at the gene and transcript levels (80). In addition, Leslie PL et al. demonstrated that LRP1 was upregulated at the transcriptional level and protein level through direct promoter binding by p53 and p53-activating stresses (53). Based on previous work confirming the role of MIF in stabilizing the binding of p53-Mdm2, leading to suppression of p53 activity in apoptosis and cell cycle arrest, it is reasonable to presume that the elevated mRNA expression of LRP1 in MIF knockdown cells in the present study might result from MIF-induced inhibition of p53 activity (54, 81–83). More rigorous studies are needed to address this regulatory mechanism.

It is noteworthy that the present study is limited in the following aspects, which should be paid attention to during our further exploration in the effects of MIF on tumour progression: 1) Given MIF as a cytokine that secretes into the environment, considering and exploring its effect on the surrounding cells and cell-cell interactions should be a major concern and await the results of further studies; 2) The influence of MIF-p53-LRP1-uPAR signaling pathway on the ECM remodeling are need to be confirmed; 3) Although CoCl_2_-induced chemical hypoxia is one of the most commonly used models mimicking decreased oxygen concentration, there are limitations to this model. Firstly, the mechanisms by which CoCl_2_ stabilizes HIF1a/2a is not completely understood; Secondly, even HIF1a/2a is stabilized and similar transcriptional activities occurs, the transcription of distinct sets of genes not affected by low oxygen has been observed by CoCl_2_ treatment. We should make sure that the differences between effects of low oxygen concentration and CoCl_2_ in cell metabolism, transcriptional regulation and signaling pathways are adapted to our study purpose. Thirdly, in the CoCl_2_-induced chemical hypoxia model, oxygen is still present in cell culture. We must keep in mind the complex relationship between oxygen- and CoCl_2_-induced cellular responses (84–86).

**Figure 9:**
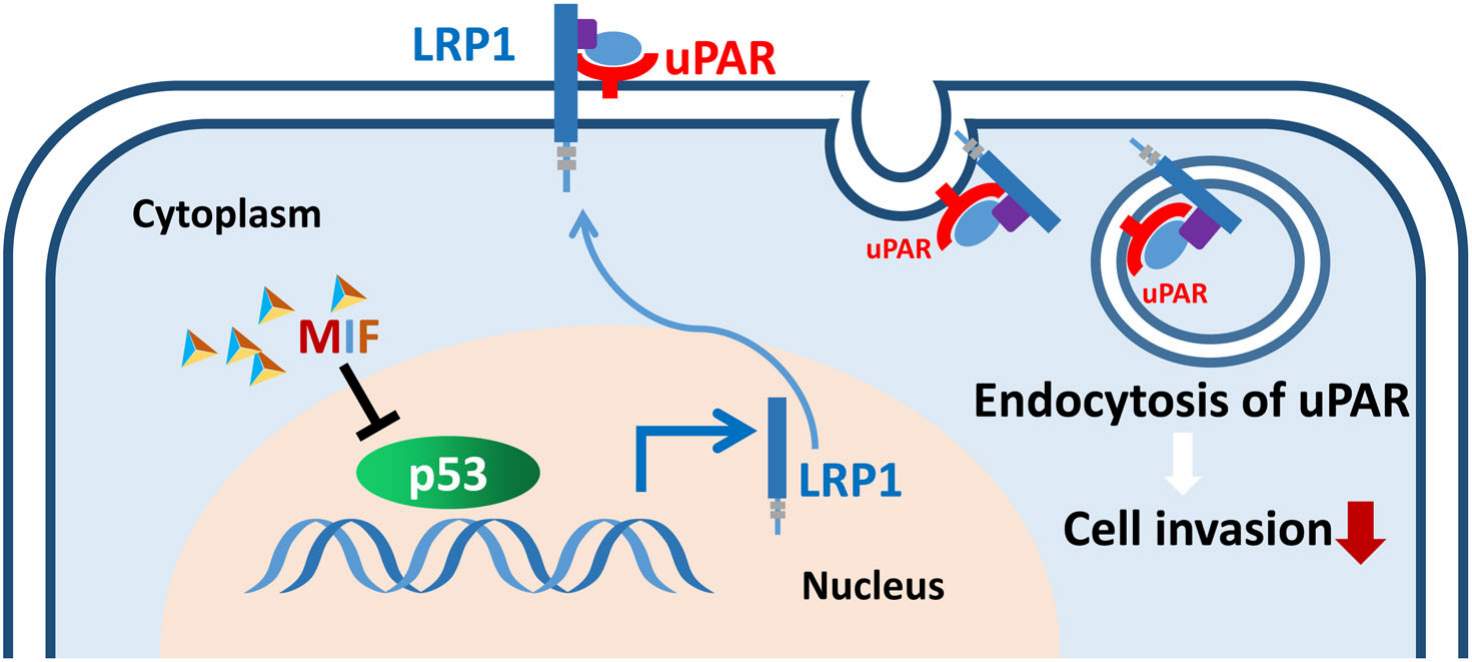
Schematic of the proposed mechanism by which the MIF-p53-LRP1-uPAR interaction promotes the invasion of PDAC cells. The interaction of MIF-p53 inhibits the degradation of p53, which upregulates the transcription of LRP1, and LRP1 then induces the internalization of uPAR and consequently weakens cell invasion.

In conclusion, our current study provides the first evidence regarding the association of LRP1-mediated uPAR endocytosis with the MIF-related invasive phenotype. It will be important to extend the invasion mechanism identified in this study to other types of malignancies. Our study also provides the potential for targeting the novel MIF-p53-LRP1-uPAR pathway (**Figure 9**) to improve the therapeutic outcome for PDAC patients.

## Data availability statement

The original contributions presented in the study are included in the article/supplementary materials. Further inquiries can be directed to the corresponding authors.

## Ethics statement

The studies involving human participants were reviewed and approved by Ethics Committee of Tianjin University Cancer Hospital. The patients/participants provided their written informed consent to participate in this study. The animal study was reviewed and approved by Institutional Animal Care and Use Committee at the Tianjin Medical University.

## Author contributions

Conceptualization, HS, DZ and CH. Methodology, HS and DZ. Software, RC and YLL. Validation, HS, YG, FL and JM. Formal analysis, YLL and RC. Investigation, HS, YL and XB. Resources, HS. Writing—original draft preparation, HS and JM. Writing—review and editing, JM and CH. Visualization, DZ, FL and JM. Supervision, CH. Project administration, CH and JM. Funding acquisition, CH. All authors contributed to the article and approved the submitted version.
