# Supplementary material for: MIF promotes cell invasion by the LRP1-uPAR interaction in pancreatic cancer cells
Source: Front Oncol. 2023 Jan 10;12:1028070. doi: 10.3389/fonc.2022.1028070 (PMC9871987; doi:10.3389/fonc.2022.1028070)

CG000238 Rev A

USER GUIDE

Visium Spatial Gene Expression Reagent Kits - Tissue Optimization

FOR USE WITH

Visium Spatial Tissue Optimization Slide & Reagent Kit, 4 slides PN-1000193

Visium Accessory Kit, PN-1000194

10x GENOMICS

Notices

Document Number

CG000238 • Rev A

Legal Notices

© 2019 10X Genomics, Inc (10x Genomics). All rights reserved. Duplication and/or reproduction of all or any portion of this document without the express written consent of 10x Genomics, is strictly forbidden. Nothing contained herein shall constitute any warranty, express or implied, as to the performance of any products described herein. Any and all warranties applicable to any products are set forth in the applicable terms and conditions of sale accompanying the purchase of such product. 10x Genomics provides no warranty and hereby disclaims any and all warranties as to the use of any third-party products or protocols described herein. The use of products described herein is subject to certain restrictions as set forth in the applicable terms and conditions of sale accompanying the purchase of such product. A non-exhaustive list of 10x Genomics' marks, many of which are registered in the United States and other countries can be viewed at: www.10xgenomics.com/trademarks. 10x Genomics may refer to the products or services offered by other companies by their brand name or company name solely for clarity, and does not claim any rights in those third party marks or names. 10x Genomics products may be covered by one or more of the patents as indicated at: www.10xgenomics.com/patents. The use of products described herein is subject to 10x Genomics Terms and Conditions of Sale, available at www.10xgenomics.com/legal-notices, or such other terms that have been agreed to in writing between 10x Genomics and user. All products and services described herein are intended FOR RESEARCH USE ONLY and NOT FOR USE IN DIAGNOSTIC PROCEDURES.

Licensed Software Updates Warranties

Updates to existing Licensed Software may be required to enable customers to use new or existing products.

Support

Email: support@10xgenomics.com

10x Genomics

6230 Stoneridge Mall Road

Pleasanton, CA 94588 USA

Document Revision Summary

**Document Number**	CG000238
**Title**	Visium Spatial Gene Expression Reagent Kits - Tissue Optimization User Guide
**Revision**	Rev A
**Revision Date**	November 2019

- This is Rev A of the User Guide.

Table of Contents

Introduction 5

Visium Spatial Tissue Optimization Reagent Kits 6

Visium Accessories 7

Recommended Thermal Cyclers 7

Imaging System Recommendations 8

Additional Kits, Reagents & Equipment 9

Protocol Steps & Timing 10

Stepwise Objectives 11

Tips & Best Practices 13

Sample Preparation Guidelines 22

Step 1 24

Tissue Fixation & Imaging 25

1.1 Tissue Fixation 26

1.2 Tissue Staining 27

1.3 Tissue Imaging 28

Step 2 29

Permeabilization & cDNA Synthesis 30

2.1 Tissue Permeabilization 32

2.2 Fluorescent cDNA Synthesis 33

Step 3 34

Tissue Removal 35

3.1 Tissue Removal 36

3.2 Slide Imaging 37

Imaging Guidelines 38

Troubleshooting 41

Appendix 44

Coverslip Application & Removal 45

Introduction

Visium Spatial Tissue Optimization Reagent Kits
Visium Accessories
Recommended Thermal Cyclers
Imaging System Recommendations
Additional Kits, Reagents & Equipment
Protocol Steps & Timing
Stepwise Objectives

Visium Spatial Tissue Optimization Reagent Kits

Visium Spatial Tissue Optimization Slide & Reagent Kit, 4 slides PN-1000193

Visium Spatial Tissue Optimization Reagent Kit, 4 slides PN-1000192 (store at –20°C)

Visium
Spatial Tissue Optimization Reagent Kit

	#	PN
RT Reagent C	1	2000215
RT Enzyme D	1	2000216
Template Switch Oligo	2	3000228
Reducing Agent B	1	2000087
Permeabilization Enzyme	2	2000214

10xGenomics.com 10x GENOMICS

Visium Spatial Tissue Optimization Slide Kit PN-1000191 (store at ambient temperature)

Visium
Spatial Tissue Optimization Slide Kit

	#	PN
Visium Spatial Tissue Optimization Slide	4	3000394
Slide Seals	20	3000279
Slide Cassette	4	3000406
Slide Gasket	4	3000426
Tissue Removal Buffer	1	2000221
Tissue Removal Enzyme	1	3000387

10xGenomics.com 10x GENOMICS

Visium Accessories

Product	Part Number (Kit)	Part Number (Item)
Thermocycler Adaptor	1000194	3000380
Visium Imaging Test Slide		2000235
Slide Alignment Tool		3000433

Recommended Thermal Cyclers

Supplier	Description	Part Number
Bio-Rad	C1000 Touch Thermal Cycler with 96-Deep Well Reaction Module	1851197
Eppendorf	MasterCycler Pro	North America 950030010<br>International 6321 000.019
Thermo Fisher Scientific	Veriti 96-Well Thermal Cycler	4375786

Imaging System Recommendations

The imaging systems listed below were used by 10x Genomics. Any equivalent system with the listed features may be used for imaging. Hardware compatibility may be tested by using the Visium Imaging Test Slide.

Imaging Systems & Specifications	
**Microscopes**<br>(Any equivalent system with the listed features may be used for imaging)	
Nikon	Nikon Eclipse Ti2 with brightfield and fluorescence capacity (TRITC)
Molecular Devices	ImageXpress Nano Automated Slide Imaging System
**Microscope Features**	
Objectives	• 4X (Plan APO λ; NA 0.20)<br>• 10X (Plan APO λ; NA 0.45)<br>• 20X (Plan APO λ; NA 0.75)
Automated Scanning Stage	Microscope tile scanning functionality is required for imaging tissue sections placed on a Capture Area of a Visium Spatial slide.
Brightfield Features	• Color camera (3 x 8 bit, 2424 x 2424 pixel resolution)<br>• White balancing functionality<br>• Minimum Capture Resolution 2.18 µm/pixel<br>• Exposure times 2-10 milli sec
Fluorescence Features*	• Light source (or equivalent) with a wavelength range of 380-680 nm<br>• Monochrome camera (14 bit, 2,424 x 2,424 pixel resolution)<br>• TRITC filter cube (Excitation 542/20, Emission 620/52)<br>(only required for Tissue Optimization protocol)<br>• Minimum Capture Resolution 2.18 µm/pixel<br>• Exposure times 100 milli sec-2 sec

* Only required for Visium Spatial Tissue Optimization protocol & Visium Imaging Test Slide verification

Additional Specifications	
Image Format	Save image as a tiff (preferred) or jpeg
Computer	Computer with sufficient power to handle large images (0.5-5 GB)
Software	Image stitching software<br>(microscope's software or equivalent, like Image J)

Additional Kits, Reagents & Equipment

The items in the table below have been validated by 10x Genomics and are highly recommended for Visium Spatial Reagent Kits protocols. Substituting materials may adversely affect system performance. This list does not include standard laboratory equipment, such as water baths, centrifuges, pH meters, vortex mixers, freezers, etc.

Supplier	Description	Part Number (US)
**Plastics**		
Eppendorf	DNA LoBind Tubes, 1.5 ml	022431021
Corning	Self-Standing Polypropylene Centrifuge Tubes, 50 ml, sterile<br>Corning 250 mL Vacuum System, 0.2 µm Pore 19.6cm^2 NY Membrane	430921<br>430771
Rainin	Tips LTS 20UL Filter RT-L10FLR<br>Tips LTS 200UL Filter RT-L200FLR<br>Tips LTS 1ML Filter RT-L1000FLR	30389226<br>30389240<br>30389213
VWR	Divided Polystyrene Reservoirs	41428-958
Thermo Fisher Scientific	Simport Scientific LockMailer Tamper Evidence Slide Mailer<br>(alternatively, use a 50-ml centrifuge tube)	22-038-399
**Kits & Reagents**		
Thermo Fisher Scientific	Nuclease-free Water<br>Low TE Buffer (10 mM Tris-HCl pH 8.0, 0.1 mM EDTA)<br>Tris Base (White Crystals or Crystalline Powder/Molecular Biology)<br>Universal Mouse Reference RNA<br>(Optional. Alternatively, use any bulk mRNA. 1µg/µl, RIN ≥ 7)	AM9937<br>12090-015<br>BP152-500<br>QS0640
Millipore Sigma	SSC Buffer 20X Concentrate<br>Sodium dodecyl sulfate (SDS) solution, 10% in water<br>Methanol, for HPLC, ≥99.9%<br>2-Propanol (Isopropanol), ≥99.5%<br>Eosin Y Solution, aqueous, 0.5% (w/v) in water<br>Acetic acid, ≥99.9%	S66391L<br>71736<br>34860<br>I9516 - 25 ml<br>HT110216 - 500 ml<br>A6283
Fisher Chemical	Hydrochloric Acid Solution, 0.1N	SA54-1
Agilent	Hematoxylin, Mayer's (Lillie's Modification)<br>(alternatively, use Mayer's Hematoxylin from Electron Microscopy Services)<br>Bluing Buffer, Dako	S330930-2<br>2638102<br>CS70230-2
-	Ultrapure/Milli-Q water (from Milli-Q Integral Ultrapure Water System or equivalent)	
**Equipment**		
Labnet	Slide Spinner<br>(alternatively, use a 50-ml centrifuge tube in a centrifuge with a swing-bucket rotor)	C1303-T
Rainin	Pipet-Lite Multi Pipette L8-200XLS+<br>Pipet-Lite LTS Pipette L-2XLS+<br>Pipet-Lite LTS Pipette L-10XLS+<br>Pipet-Lite LTS Pipette L-20XLS+<br>Pipet-Lite LTS Pipette L-100XLS+<br>Pipet-Lite LTS Pipette L-200XLS+<br>Pipet-Lite LTS Pipette L-1000XLS+	17013805<br>17014393<br>17014388<br>17014392<br>17014384<br>17014391<br>17014382

Protocol Steps & Timing

4 h

Steps		Timing
**Step 1 – Tissue Staining & Imaging**		
1.1	Tissue Fixation	35 min
1.2	Tissue Staining	30 min
1.3	Tissue Imaging*	Variable
**Step 2 – Permeabilization & cDNA Synthesis**		
2.1	Tissue Permeabilization	45 min
2.2	Fluorescent cDNA Synthesis	60 min
**Step 3 – Tissue Removal**		
3.1	Tissue Removal	70 min
3.2	Slide Imaging*	Variable

*~4 h workflow, excluding imaging steps

Stepwise Objectives

The Visium Spatial Gene Expression Solution measures total mRNA in intact tissue sections and maps where that gene activity is occurring. Prior to library preparation, the Visium Spatial Tissue Optimization workflow allows the user to optimize permeabilization conditions for a tissue of interest. Tissue sections are placed onto corresponding Capture Areas on the Visium Spatial Tissue Optimization Slide. These sections are fixed, stained, and permeabilized for different lengths of time. mRNA released during permeabilization binds to capture probes on the slide. cDNA is generated using fluorescently labeled nucleotides to visualize synthesized cDNA. Finally, the tissue is enzymatically removed, leaving fluorescently labeled cDNA that may be visualized using fluorescence microscopy to select the optimal permeabilization time.

At the successful completion of this protocol, users should proceed to library preparation using the Visium Spatial Gene Expression Reagent Kits User Guide (CG000239).

Visium Spatial Tissue Optimization Slide

Each Capture Area on the Visium Spatial Tissue Optimization Slide is defined by an etched frame. Each Capture Area contains oligonucleotides for mRNA capture. Each probe has poly(dT) primers that enables the production of cDNA from poly-adenylated mRNA. These probes do not contain a spatial barcode.

There are eight Capture Areas per slide. Seven of these are used for tissue and one is used for a positive RNA control. Only one tissue type should be tested per slide.

Visium Spatial Tissue Optimization Slide

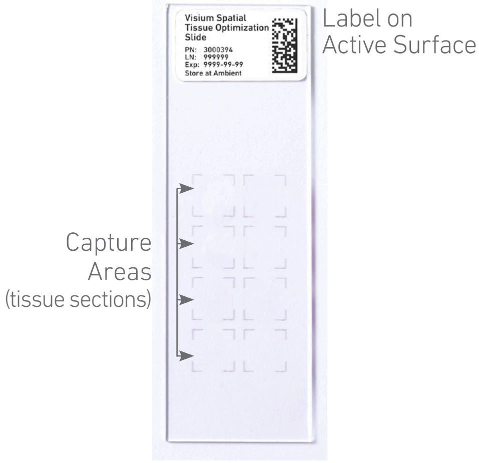

Step 1
Tissue Staining & Imaging

Tissue is cryosectioned and placed on Capture Areas on the Visium Spatial Tissue Optimization slide. Tissue sections are then fixed with methanol and stained with hematoxylin and eosin (H&E). Hematoxylin stains the nuclei of mammalian cells, while Eosin stains the extracellular matrix and cytoplasm. Stained tissue sections are imaged for comparison with final fluorescent images.

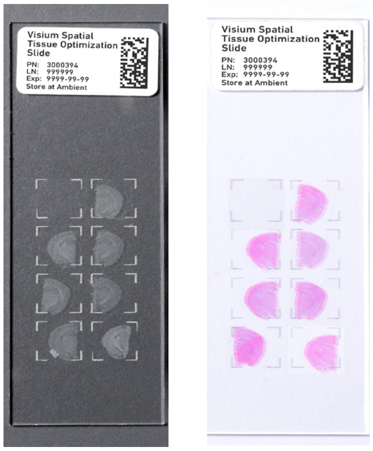

Slides in image are representative.

Step 2
Permeabilization & Fluorescent cDNA Synthesis

Tissue sections are permeabilized for varying amounts of time. Each Capture Area captures poly-adenylated mRNA from the attached tissue section. A Master Mix containing reverse transcription (RT) reagents and fluorescently labeled nucleotides is added on top of the tissue sections, resulting in fluorescently labeled cDNA.

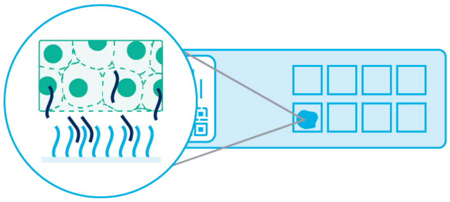

Step 3
Tissue Removal

Tissue is enzymatically removed, leaving behind fluorescent cDNA covalently linked to oligonucleotides on the Visium Spatial Tissue Optimization slide. Fluorescent cDNA is visualized under fluorescence imaging conditions verified using the Visium Imaging Test Slide. H&E and fluorescence images are compared. The permeabilization time that results in maximum fluorescence signal with the lowest signal diffusion is optimal. If the signal is the same at two time points, the longer permeabilization time is considered optimal.

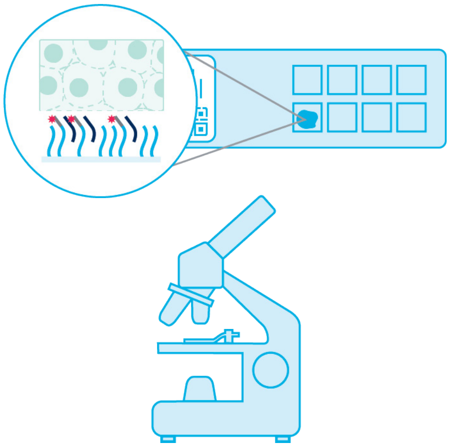

Tips & Best Practices

TIPS

Icons	TIPS<br>Tips & Best Practices section includes additional guidance	Signifies critical step requiring accurate execution	Troubleshooting section includes additional guidance

General Reagent Handling

- Fully thaw and thoroughly mix reagents before use.
- Keep RT Master Mix on ice during setup and use. Promptly move reagents back to the recommended storage.
- Use a pH meter to adjust pH as necessary during buffer preparation.

Pipette Calibration

- Follow manufacturer's calibration and maintenance schedules.

Visium Spatial Tissue Optimization Slide

- Includes 8 Capture Areas (8 x 8 mm), covered with oligonucleotides for mRNA capture.
- The active surface of the slide is defined by a readable label.
- The tissue sections are always placed on the Capture Areas on the active surface. For more information, consult the Visium Spatial Protocols – Tissue Preparation Guide (Demonstrated Protocol CG000240).

Visium Spatial Tissue Optimization Slide

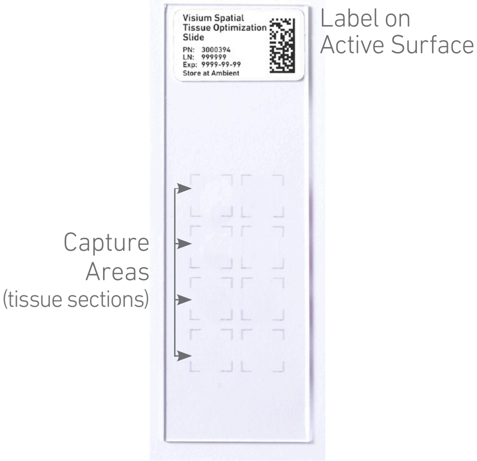

Slide Storage

- Always store slides in a cool, dry environment.
- Store unused slides in original packaging and keep sealed. DO NOT remove desiccant. If necessary, store original packaging in a secondary container, such as a resealable bag.
- After tissue placement, store the slides at **–80°C** in a sealed container.

Slide Storage

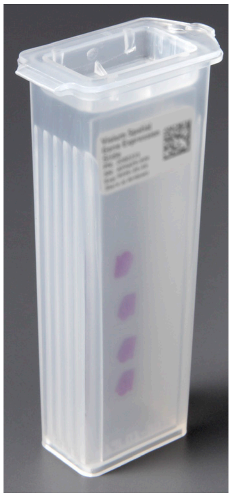

Slides in image are representative.

Slide Handling

- Always wear gloves when handling slides.
- Ensure that the active surface of a slide faces up and is never touched. The orientation of the label on the slide defines the active surface.
- The tissue sections should always be on the active surface of the slide. DO NOT touch the tissue sections on the slide.
- Minimize exposure of the slides to sources of particles and fibers.
- When immersing slides in water, ensure that the tissue sections are completely submerged.
- Keep the slide flat on the bench when adding reagents to the active surface.
- Ensure that no absorbent surface is in contact with the reagents on the slide during incubation.

Active Surface with Tissue Sections

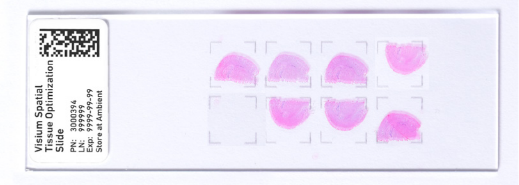

Immersing Slide

Correct Incorrect

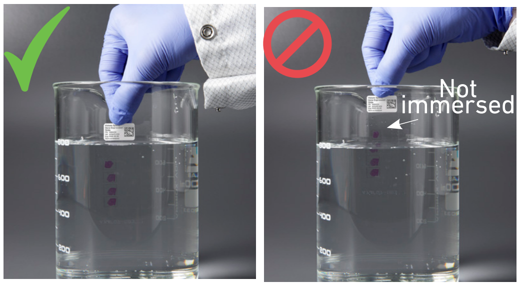

Reagent on Slide

Correct Incorrect

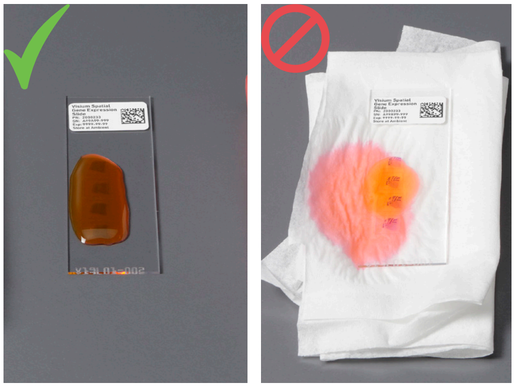

Slides in image are representative.

Slide Cassette

- The Slide Cassette encases the slide and creates leakproof wells for adding reagents.
- Place the slides in the Slide Cassette only when specified.
- The Slide Cassette is disposable and intended for one-time use.
- An Insert Clip and four tabs at the back of the Slide Cassette are used for holding the slide in the cassette, as shown.
- The cassette includes a removable gasket (disposable; one-time use) corresponding to the Capture Areas on the slides.
- The Slide Cassette may be assembled using the Slide Alignment Tool or manually. Instructions for both are provided in the following section.
- See Slide Cassette Assembly & Removal instructions for details.
- Ensure that the back of the Slide Cassette is facing the user prior to assembly. The active surface of the slide with tissue sections will face down such that the slide label is no longer readable.
- Practice assembly with a plain glass slide.
- Applying excessive force to the slide may cause the slide to break.

Slide Cassette

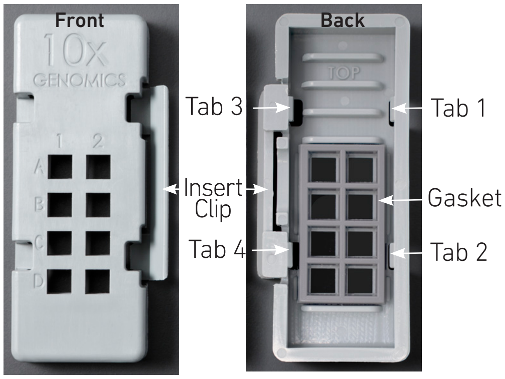

Slide Alignment Tool

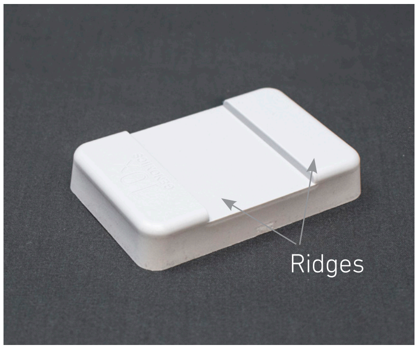

Slide Cassette Assembly

Position Slide Cassette along alignment tool ridges

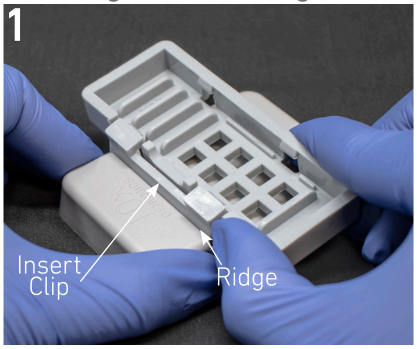

Push Insert Clip along the ridge & press Slide Cassette down

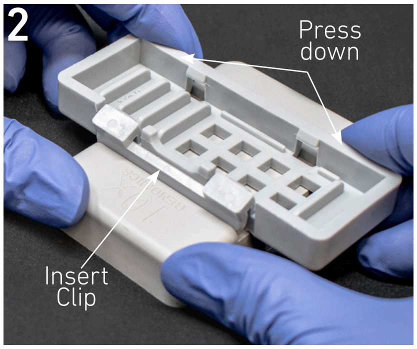

Slide Cassette secured on alignment tool

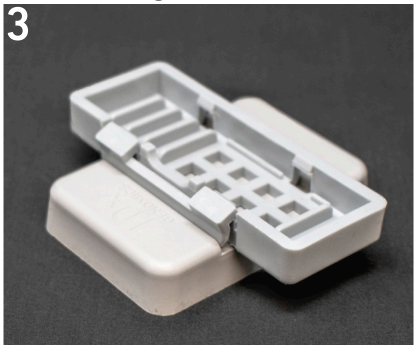

Position Gasket to align with Slide Cassette cutouts

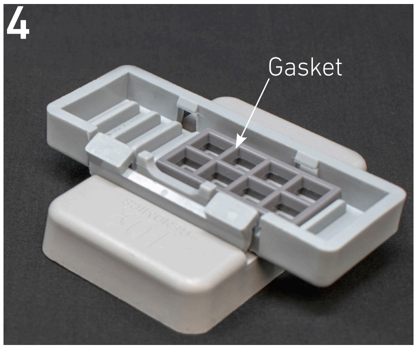

Insert long edge of slide under tabs 1 & 2; ensure slide is flush

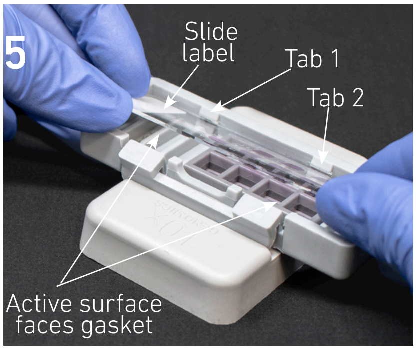

Slide insertion may push gasket out of alignment with slide cutouts. Adjust if necessary.

Press slide down until it is flush with the gasket and under tabs 3 & 4

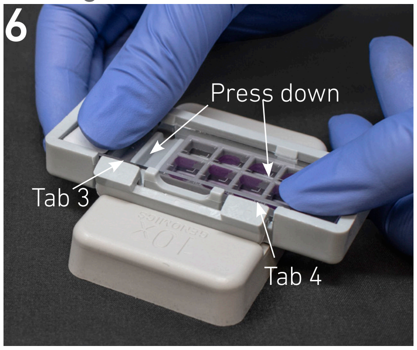

Remove Slide Cassette while pressing slide against the gasket

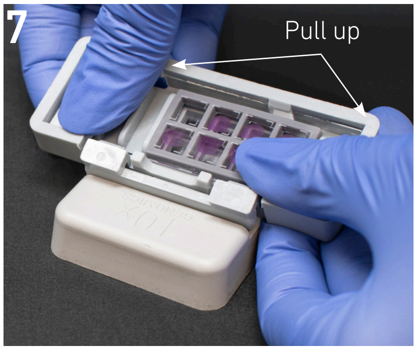

Slide Cassette Removal

Position Slide Cassette along alignment tool ridges

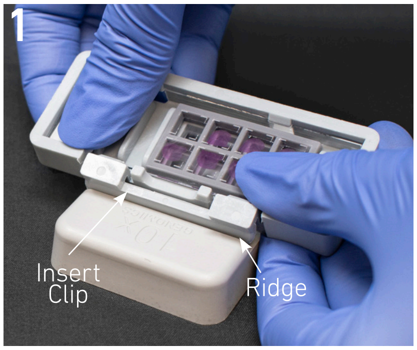

Push Insert Clip along the ridge & press down

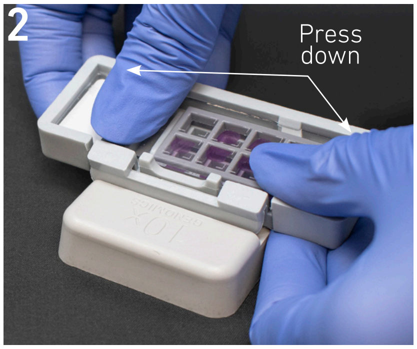

Slide Cassette sits securely on alignment tool

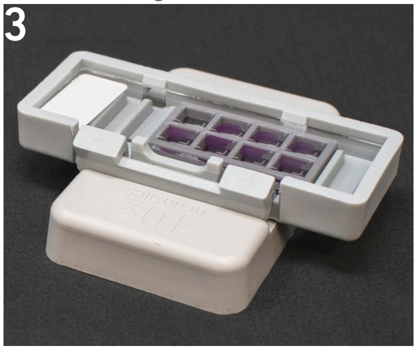

Lift slide at Slide Cassette groove

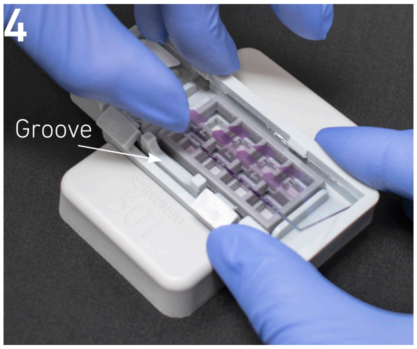

Manual Slide Cassette Assembly & Removal

Assembly

i. Insert the gasket and align the gasket and Slide Cassette cutouts.

ii. Align the label on top of the slide to the top of the Slide Cassette, as shown.

iii. Insert the slide under tabs 1 and 2. Ensure that the long edge of the slide is flush with the side of the Slide Cassette.

iv. Press the insert clip **very firmly** by applying even force on the lower part of the insert clip.

v. Press down on the slide with a finger in between tabs 3 and 4 until the slide is under each tab and release the insert clip.

Removal

i. Press the insert clip **very firmly** to release the slide from the cassette.

ii. Lift slide at Slide Cassette groove between tabs 3 and 4 until the slide can be removed.

Slide Cassette

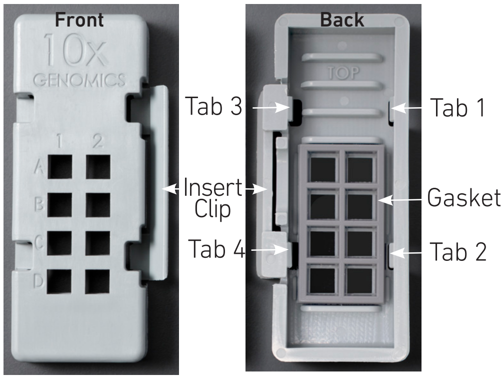

Slide Cassette Assembly

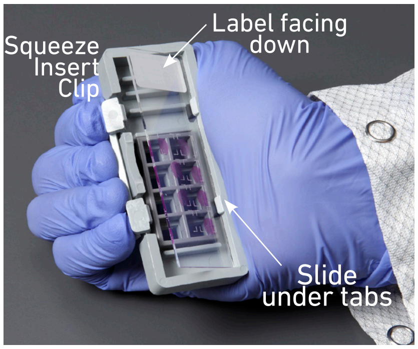

Insert Clip - Press Firmly

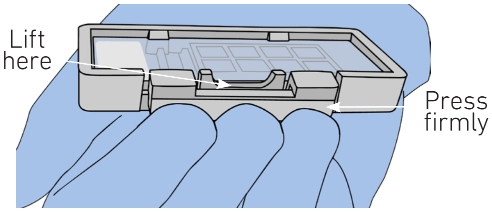

Reagent Addition & Removal from Wells

- Place the assembled slide in the slide cassette flat on the bench.
- Dispense and remove reagents along the side of the wells without touching the tissue sections and without introducing bubbles.
- Always cover the tissue section completely when adding reagents to the well. A gentle tap may help spread the reagent more evenly.
- Ensure that no bubbles are introduced in the process.

Reagent Addition/Removal

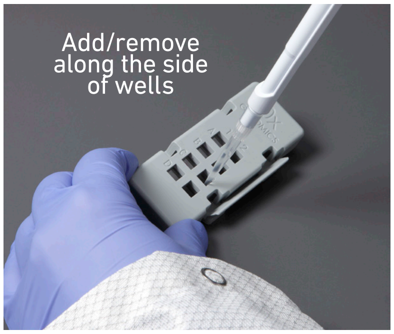

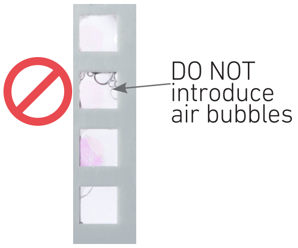

Slide Seal Application & Removal

Application

- Place the Slide Cassette flat on the bench.
- Remove the back of the adhesive Slide Seal.
- Align the Slide Seal with the surface of the Slide Cassette and apply while firmly holding the Slide Cassette.
- Press on the Slide Seal to ensure uniform adhesion.

Removal

- Place the Slide Cassette flat on the bench.
- Pull on the Slide Seal from the edge while firmly holding the Slide Cassette. Ensure that no liquid splashes out of the wells.

Slide Seal Application

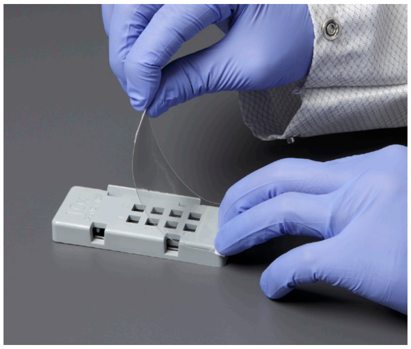

Slide Incubation Guidance

Incubation at a specified temperature

- Position a Thermocycler Adaptor on a thermal cycler that is set at the incubation temperature.
- Ensure that the Thermocycler Adaptor is in contact with the thermal cycler surface uniformly.
- When incubating a slide, position the slide on the Thermocycler Adaptor with the active surface facing up.
- Ensure that the entire bottom surface of the slide is in contact with Thermocycler Adaptor.
- When incubating a slide encased in a Slide Cassette, place the assembled unit on the Thermocycler Adaptor with the wells facing up. The Slide Cassette should always be sealed when on the Thermocycler Adaptor.

Place Thermocycler Adaptor

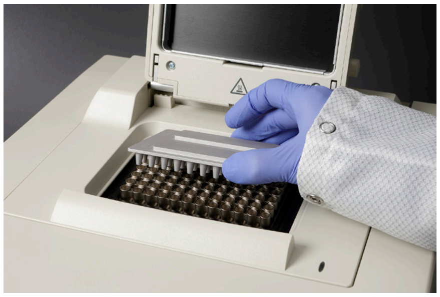

Incubate Slide

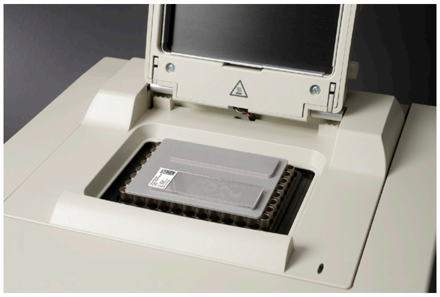

Incubate Assembled Slide Cassette

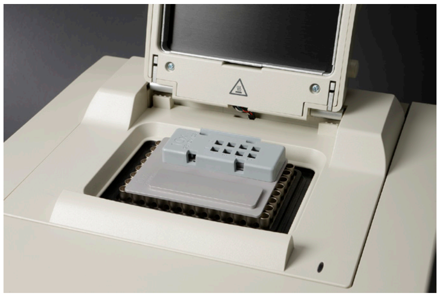

Incubation at room temperature

- Place the slide/Slide Cassette on a flat, clean work surface.
- Ensure that no absorbent surface is in contact with the reagents on the slide during incubation.

Slide Incubation

Correct Incorrect

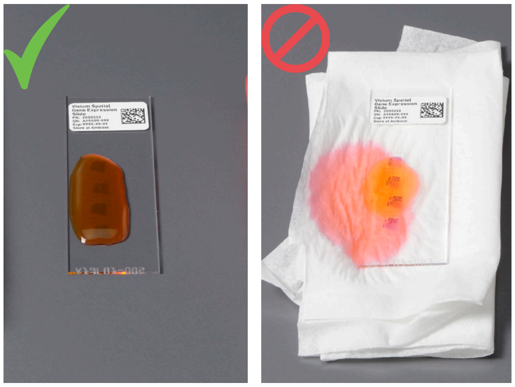

Slides in image are representative.

Sample Preparation Guidelines

Sample Preparation Guidelines

Proper tissue handling and preparation techniques are critical in preserving the morphological quality of the tissue sections and subsequent transcript availability in Visium Spatial protocols.

Listed below are some key considerations for preparing samples that are compatible with the Visium Spatial protocols.
Consult the Visium Spatial Protocols – Tissue Preparation Guide for complete information (Demonstrated Protocol CG000240).

Key Considerations

Slide Handling (before sectioning)

- ☐ Equilibrate Visium slides to cryostat temperature before cryosectioning.
- ☐ Store unused slides in original packaging and keep sealed. DO NOT remove desiccant. If necessary, store original packaging in a secondary container such as a resealable bag.

Freezing and Embedding

- ☐ Snap freeze samples in a bath of isopentane and liquid nitrogen.
- ☐ Store frozen samples at **-80°C** in a sealed container for **long-term** storage prior to embedding.

Cryosectioning

- ☐ Equilibrate OCT tissue block to the cryostat chamber temperature for **30 min**.
- ☐ Place tissue sections on the Capture Area within the etched frame on the slide.
- ☐ Leave one Capture Area empty for the positive control.

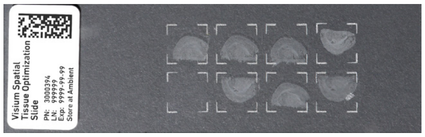

Slide Handling (after sectioning)

- ☐ Maintain slides containing sections in a low moisture environment.
- ☐ Keep slides cold and transport slides on dry ice.
- ☐ DO NOT leave slides at room temperature.

Sample Storage

- ☐ Store slides individually in a sealed container at **-80°C** for up to **a week** to avoid multiple freeze thaw cycles. If necessary, place the sealed container in a secondary container, such as a resealable bag.

Step 1

Tissue Staining & Imaging

1.1 Tissue Fixation

1.2 Tissue Staining

1.3 Tissue Imaging

1.0 Tissue Staining & Imaging

CHECKLIST – GET STARTED!

	Items	10x PN	Preparation & Handling	Storage
**Place at -20°C**				
☐	**Methanol**<br>Dispense 40 ml/slide* in a 50-ml centrifuge tube	-	Chill to **-20°C** before use	Ambient
**Obtain**				
☐	**Visium Spatial Tissue Optimization Slide**<br>(with tissue sections)	3000394	For sample preparation, consult the Visium Spatial Protocols – Tissue Preparation Guide (CG000240).	-80°C
☐	**Slide Cassette**	3000406	See Tips & Best Practices.	Ambient
☐	**Isopropanol**	-	Manufacturer's recommendations.	Ambient
☐	**Hematoxylin, Mayer's (Lillie's Modification)**	-	Manufacturer's recommendations.	Ambient
☐	**Eosin Y Solution**	-	Manufacturer's recommendations.	Ambient
☐	**Bluing Buffer**	-	Manufacturer's recommendations.	Ambient
☐	**Milli-Q Water**	-	-	Ambient
☐	**250 ml Vacuum Filter/ Storage Bottle System**<br>(0.2 µm Filter Nylon membrane)	-	-	Ambient
☐	**Tris Base**	-	Manufacturer's recommendations.	-
☐	**Acetic Acid**	-	Manufacturer's recommendations.	-
**Prepare**				
☐	**Tris-Acetic Acid Buffer**<br>(0.45 M, pH 6.0)	Prepare **200 ml**, store at **room temperature**.<br>• Dissolve **11 g** Tris base in **100 ml** nuclease-free water.<br>• Adjust pH to 6.0 using 100% Acetic Acid.<br>• Bring volume to **200 ml** with nuclease-free water.<br>• Filter through **0.2 µm** nylon filter.		

*If using a Simport Scientific LockMailer Slide Mailer, dispense 10 ml/slide of methanol.

⚠ Ensure that microscope settings have been optimized to capture brightfield images.

Consult the Visium Spatial Gene Expression Imaging Guideline Technical Note (CG000241) for more information.

1.1 Tissue Fixation

Ensure that the methanol (40 ml/slide) dispensed in a 50-ml centrifuge tube is chilled to **-20°C**.

a. Place a Thermocycler Adaptor on a thermal cycler set at **37°C** and equilibrate for **5 min**. Heating the thermal cycler lid is not required.

b. Remove the slide from **-80°C** and place on dry ice in a sealed container.

⚠ Delay in transferring slides to dry ice may result in condensation, which may cause tissue damage and/or shifting of tissue sections on the slide.

c. Place the slide on the Thermocycler Adaptor with the active surface facing up and incubate **1 min** at **37°C**. DO NOT close the thermal cycler lid. Maintain thermal cycler at **37°C** for step 1.2.

d. If necessary, wipe excess liquid from the back of the slide, without touching the tissue sections.

e. Completely immerse slide in the pre-chilled methanol. Secure the tube cap to prevent methanol loss.

f. Incubate **30 min** at **-20°C**.

Place Thermocycler Adaptor

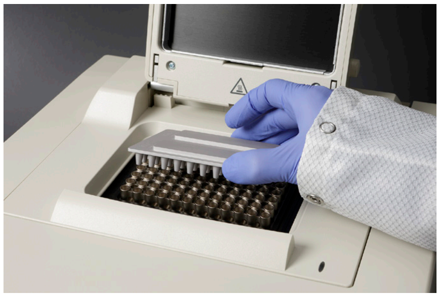

Incubate Slide for 1 min at 37°C

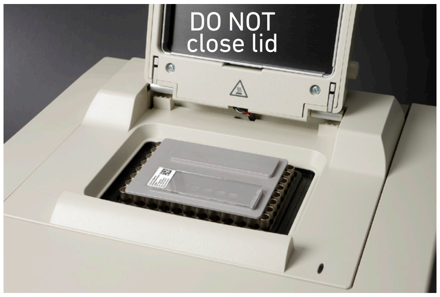

Incubate in Methanol for 30 min at –20°C

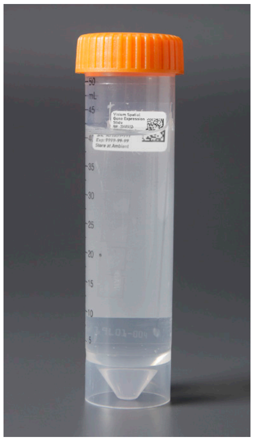

1.2 Tissue Staining

a. Dispense the following volumes of Milli-Q water.
50 ml in one 50-ml centrifuge tube/slide
800 ml in Beaker 1
800 ml in Beaker 2
800 ml in Beaker 3
Dispensed volume in each beaker can be used for two slides.

b. Prepare Eosin Mix. DO NOT add pure Eosin to tissue sections.

Eosin Mix<br>*Prepare Fresh. Vortex, centrifuge briefly.*	Volume/slide (µl)
Eosin	100
Tris-Acetic Acid Buffer (0.45 M, pH 6.0)	900
**Total**	**1,000**

c. Remove the slide from methanol and wipe excess liquid from the back of the slide, without touching the tissue sections. Place on a flat, clean work surface. Some residual droplets may remain.

TIPS

d. Add **500 µl** isopropanol to uniformly cover all tissue sections. See Tips & Best Practices.

e. Incubate **1 min** at **room temperature**.
When incubating the slide with reagents, ensure that the slide is not in contact with any absorbent surface, like laboratory wipes, which may absorb the reagents.

Incubate with Reagent

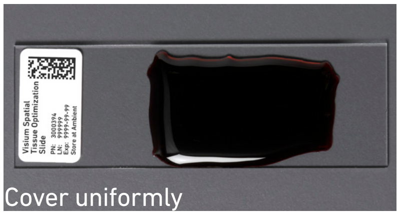

f. Discard reagent by draining and/or holding the slide at an angle with the bottom edge in contact with a laboratory wipe.

g. Wipe excess liquid from the back of the slide, without touching the tissue sections. Place on a flat, clean work surface.

h. Air dry the slide. To prevent tissue from over drying, DO NOT exceed **10 min**.

i. Add **1 ml** Hematoxylin to uniformly cover all tissue sections on the slide.

j. Incubate **7 min** at **room temperature**.

k. Discard reagent by draining and/or holding the slide at an angle with the bottom edge in contact with a laboratory wipe.

Discard Reagent

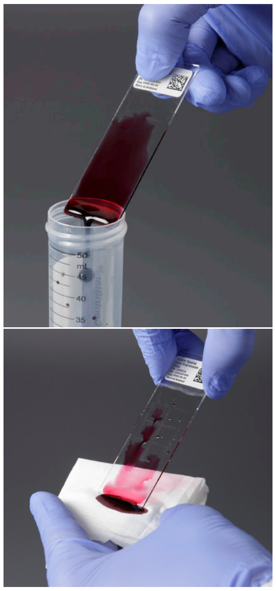

Slides in image are representative.

l. Immerse the slide 5x in the water in the 50-ml centrifuge tube.

m. Immerse the slide 15x in the water in Beaker 1.

n. Immerse the slide 15x in the water in Beaker 2.

o. Wipe excess liquid from the back of the slide without touching the tissue sections. Place on a flat, clean work surface. Some droplets may remain.

Each immersion is ~1 sec

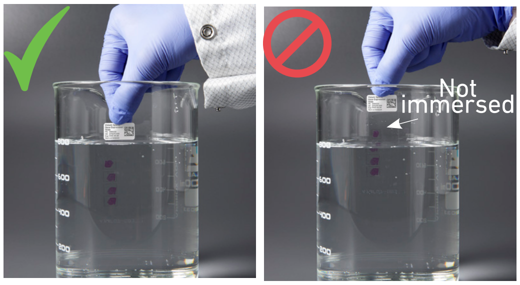

p. Add **1 ml** Bluing Buffer to uniformly cover all tissue sections.

q. Incubate **2 min** at **room temperature**.

r. Discard reagent by draining and/or holding the slide at an angle with the bottom edge in contact with a laboratory wipe.

s. Immerse the slide 5x in the water in Beaker 2.

t. Wipe excess liquid from the back of the slide without touching the tissue sections. Place on a flat, clean work surface. Some droplets may remain.

u. Add **1 ml** Eosin Mix to uniformly cover all tissue sections.

v. Incubate **1 min** at **room temperature**.

w. Discard reagent by draining and/or holding the slide at an angle with the bottom edge in contact with a laboratory wipe.

x. Immerse the slide 15x in the water in Beaker 3.

y. Wipe the back of the slide with a laboratory wipe. Place on a flat, clean work surface and air dry until tissue is opaque.

z. Incubate the slide on the Thermocycler Adaptor with the thermal cycler lid open for **5 min** at **37°C**.

Incubate Slide

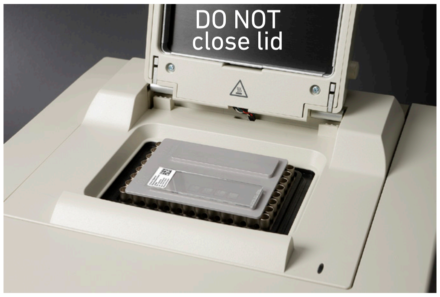

Proceed to imaging.
OPTIONAL: A coverslip may be mounted on the slide before imaging. See Appendix for Coverslip Application & Removal protocol.

1.3 Tissue Imaging

- Image all Capture Areas together at the desired magnification using brightfield imaging settings. See Imaging System Recommendations for more information.

Consult the Visium Spatial Gene Expression Imaging Guideline Technical Note (CG000241) for complete information.

Step 2

Permeabilization & cDNA Synthesis

2.1 Tissue Permeabilization

2.2 Fluorescent cDNA Synthesis

2

2.0 Permeabilization & cDNA Synthesis

CHECKLIST – GET STARTED!

Items	10x PN	Preparation & Handling	Storage
**Prepare & Equilibrate to 37°C**			
☐ **Permeabilization Enzyme**	2000214	**Immediately** before use, centrifuge briefly and resuspend in 1.2 ml HCl (0.1N), pipette mix, centrifuge briefly, verify no precipitate. Equilibrate to 37°C. ! Store unused resuspended enzyme at −20°C. DO NOT freeze-thaw more than 3x.	−20°C
**Equilibrate to room temperature**			
! ☐ **RT Reagent C** Minimize light exposure	2000215	Thaw, vortex, centrifuge briefly.	−20°C
☐ **Template Switch Oligo**	3000228	Centrifuge briefly, resuspend in 80 µl Low TE Buffer. Vortex 15 sec at maximum speed, centrifuge briefly, leave at room temperature for ≥ 30 min. After resuspension, store at −80°C.	−20°C
☐ **Reducing Agent B**	2000087	Thaw, vortex, verify no precipitate, centrifuge briefly.	−20°C
**Place on ice**			
☐ **RT Enzyme D**	2000216	Pipette mix, centrifuge briefly.	−20°C
**Obtain**			
☐ **Nuclease-free water**	-	-	Ambient
☐ **Slide Seals**	3000279	See Tip & Best Practices.	Ambient
☐ **20X SSC**	-	-	Ambient
☐ **Reference RNA**	-	Human or mouse RNA at 1µg/µl, RIN ≥ 7	−80°C
**Prepare**			
☐ **0.1X SSC** (can be prepared ahead of time)			

0.1X SSC<br>Store at room temperature	Stock	Final	50 ml
SSC	20X	0.1X	250 µl
Water	-	-	49.75 ml

Step Overview (Step 2.1)

Permeabilization Time Course

Tissue sections on the Visium Spatial Tissue Optimization Slide are incubated with the Permeabilization Enzyme for different lengths of time. This process identifies an optimal tissue permeabilization time for use in the Visium Spatial Gene Expression workflow. Optimal permeabilization ensures sufficient mRNA release and minimizes mRNA diffusion during library preparation.

A suggested time course is shown on the right. However, these times may be adjusted depending on the tissue type. The positive control well (A1) includes reference RNA without any tissue. The negative control well (D2) has a tissue section not exposed to permeabilization reagents. Permeabilization times refer to the length of time tissue sections are exposed to permeabilization reagent.

To prevent evaporation during permeabilization, the entire Slide Cassette is sealed with the Slide Seal in between reagent addition.

Tap cassette after each reagent addition to ensure uniform coverage. Confirm uniform coverage by checking the underside of the cassette.
Ensure that the Slide Seal is applied lightly to enable subsequent removal and re-application of the seal.

Suggested Permeabilization Times (min)

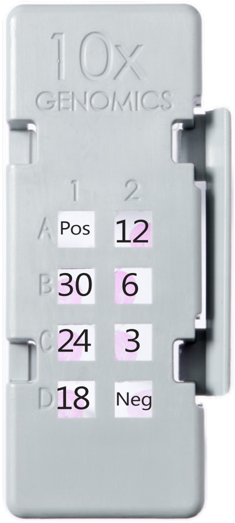

Slide Seal Application

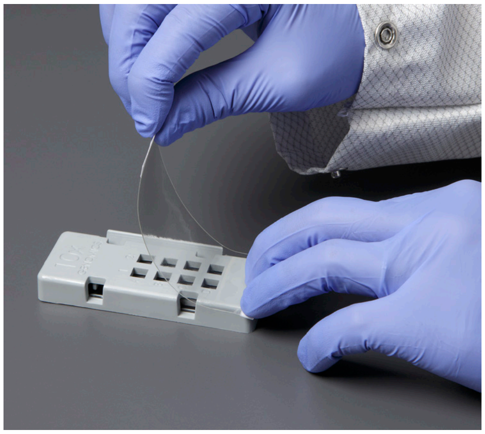

2.1 Tissue Permeabilization

如果一个盖玻片安装在切片上进行成像，请移除盖玻片。 见附录的封面应用和删除协议。 确保透化酶重悬和预热37 ° C。

a.预热适配器，设置PCR程序。

b. 将切片放在切片盒中。 见技巧和最佳做法。 用空白幻灯片练习。

TIPS

c.阳性对照(A1，无组织)：在孔中加入RNA。不加渗透酶。

d.阴性对照 (D2) :不加渗透化酶。

e. 透化时间设置：
i. 沿孔边缘向B1中加入70ul 透化酶。(30min)和抽头盒

二. 密封切片盒，并放置在37 ° C的热循环适配器上。 关闭盖子。

三. 在6分钟后，从Thermocycler适配器中取出切片盒，并拆下密封。

四. 在C1中加入70 μl透化酶，并在适配器在37 ° C，与盖子关闭。

v.重复过程，向后工作到最短的孵育时间(C2 ，3分钟)。

f.在时间过程完成后，从热循环适配器上拆下幻灯片盒。

g.使用吸管，在不接触组织切片的情况下，从每口井中去除渗透酶。 h.添加 除阳性对照(A1)外，所有孔加100μl 0.1X SSC。

If a coverslip was mounted on the slide for imaging, remove the coverslip. See Appendix for Coverslip Application & Removal protocol.
Ensure Permeabilization Enzyme is resuspended and is maintained at **37°C**.

a. Place a Thermocycler Adaptor in the thermal cycler. Prepare a thermal cycler with the following incubation protocol and start the program.

Lid Temperature		Run Time
37°C		*
**Step**	**Temperature**	**Time**
Pre-equilibrate	37°C	Hold
Permeabilization	37°C	Hold

b. Place the slide in the Slide Cassette. See Tips & Best Practices. Practice with a blank slide.

c. **Positive control (A1, no tissue):**
Add 2 µl of RNA (1 µg/µl) to the center of the well. DO NOT add Permeabilization Enzyme.

d. **Negative Control (D2):**
DO NOT add Permeabilization Enzyme.

e. **Permeabilization Time Course:**

i. Add **70 µl** Permeabilization Enzyme to **B1** along the side of the well (**30 min**) and tap cassette.

ii. Apply Slide Seal to the Slide Cassette and place on the Thermocycler Adaptor at **37°C.** Close lid.

iii. After **6 min**, remove the Slide Cassette from Thermocycler Adaptor and remove seal.

iv. Add **70 µl** Permeabilization Enzyme along the side of the well to the **C1** and incubate on the Thermocycler Adaptor at **37°C** with the lid closed.

v. Repeat process, working backwards to the shortest incubation time (C2, **3 min**).

f. Remove Slide Cassette from the Thermocycler Adaptor after time course completion.

g. Using a pipette, remove Permeabilization Enzyme from each well without touching the tissue sections.

h. Add **100 µl** 0.1X SSC to all wells except the positive control (A1).

Slide Cassette Assembly

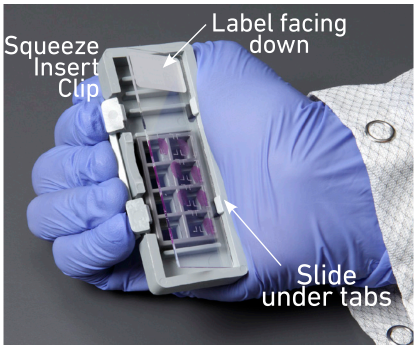

Permeabilization Times (min)

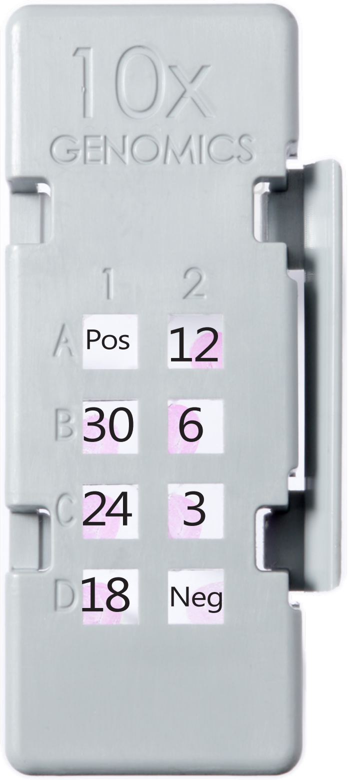

2.2 Fluorescent cDNA Synthesis

a. Place a Thermocycler Adaptor in the thermal cycler. Prepare a thermal cycler with the following incubation protocol and start the program.

Lid Temperature	Reaction Volume	Run Time
53°C	-	45 min
**Step**	**Temperature**	**Time**
Pre-equilibrate	53°C	Hold
cDNA Synthesis	53°C	00:45:00
Hold	4°C	-

b. Prepare Fluorescent RT Master Mix on ice. Pipette mix 10x and centrifuge briefly.

Fluorescent RT Master Mix<br>*Add reagents in the order listed*	PN	Volume/slide + 10% (µl)
**Nuclease-free water**	-	221.8
**RT Reagent C**<br>Minimize light exposure	2000215	110.0
**Template Switch Oligo**	3000228	30.8
**Reducing Agent B**	2000087	8.8
**RT Enzyme D**	20000216	68.6
**Total**	-	**440**

c. Using a pipette, remove 0.1X SSC from each well.

d. Add **50 µl** Fluorescent RT Master Mix to each well, ensuring that the well surface is uniformly covered with the Master Mix.

e. Apply Slide Seal on the Slide Cassette firmly and place on the Thermocycler Adaptor on the pre-heated thermal cycler. Close thermal cycler lid.

f. Skip Pre-equilibrate to initiate cDNA Synthesis.

Step 3

Tissue Removal

3.1 Tissue Removal
3.2 Slide Imaging

3

3.0
Tissue Removal

CHECKLIST – GET STARTED!

	Item	10x PN	Preparation & Handling	Storage
**Equilibrate to room temperature**				
☐	**Tissue Removal Buffer**	2000221	Pipette mix, verify no precipitate. If necessary, heat at 50°C for 5 min to remove precipitate.	Ambient
☐	**Tissue Removal Enzyme**	3000387	Vortex, centrifuge briefly.	Ambient
**Obtain**				
☐	**Thermocycler Adaptor**	3000380	-	Ambient
☐	**Slide Seals**	3000279	-	Ambient
**Prepare**				
☐	**2X SSC/0.1% SDS** (can be prepared ahead of time)		See table below	
☐	**0.2X SSC** (can be prepared ahead of time)		See table below	
☐	**0.1X SSC**		Prepared in step 2.0.	

2X SSC/0.1% SDS<br>Pre-warm to 50°C.<br>Store at room temperature.	Stock	Final	45 ml
SSC	20X	2X	4.5 ml
SDS	10%	0.1%	450 µl
Water	-	-	40.05 ml

0.2X SSC<br>Store at room temperature	Stock	Final	45 ml
SSC	20X	0.2X	450 µl
Water	-	-	44.55 ml

3.1 Tissue Removal and Slide Imaging

a. Place a Thermocycler Adaptor in the thermal cycler. Prepare a thermal cycler with the following incubation protocol and start the program.

Lid Temperature	Reaction Volume	Run Time
56°C	-	60 min
**Step**	**Temperature**	**Time**
Pre-equilibrate	56°C	Hold
Tissue Removal	56°C	00:60:00
Hold	22°C	up to 24:00:00

b. Prepare Tissue Removal Mix. Pipette mix 10x (pipette set to 600 µl), centrifuge briefly, and maintain at **room temperature**.

Tissue Removal Mix<br>*Prepare Fresh*<br>*Add reagents in the order listed*	PN	Volume/slide + 10% (µl)
Tissue Removal Buffer	2000221	539
Tissue Removal Enzyme	3000387	77
**Total**	-	**616**

c. Remove the Slide Cassette from the Thermocycler Adaptor and place on a flat, clean work surface.

d. Remove the Slide Seal and using a pipette, remove Fluorescent RT Master Mix from the wells.

e. Add **100 µl** 0.1X SSC to each well.

f. Using a pipette, remove 0.1X SSC from each well.

g. Add **70 µl** Tissue Removal Mix to each well without introducing bubbles. Ensure uniform tissue coverage.

h. Apply Slide Seal on the Slide Cassette and place on the Thermocycler Adaptor. Close the thermal cycler lid.

i. Skip Pre-equilibrate step to initiate Tissue Removal.

j. Dispense **45 ml** 2X SSC-0.1% SDS in a 50-ml centrifuge tube and pre-warm to **50°C** in a water bath or heat block.

k. Dispense **45 ml** 0.2X SSC, and **45 ml** 0.1X SSC buffers in two separate 50-ml centrifuge tubes and maintain at **room temperature**.

l. At the end of incubation, remove the Slide Seal and using a pipette, remove Tissue Removal Mix from wells.

TIPS

m. Remove the slide from the Slide Cassette. See Tips and Best Practices for more information.

n. Immerse the slide 15x in the pre-warmed 2X SSC-0.1% SDS.

o. Immerse the slide 15x in the 0.2X SSC.

p. Immerse the slide 15x in the 0.1X SSC. Wipe the back of the slide with a laboratory wipe.

q. Centrifuge for **30 sec** in a slide spinner. Alternatively, use a 50-ml centrifuge tube in a swinging bucket centrifuge and spin at **250 rcf** for **30 sec**.

DO NOT exceed 250 rcf, as slides may break at higher speeds.

r. Verify that there is no remaining tissue on the slide. If tissue remains, contact Support.

Proceed to imaging.
OPTIONAL: A coverslip may be mounted on the slide before imaging. See Appendix for Coverslip Application & Removal protocol.

3.2 Slide Imaging

- Image all Capture Areas together using the same fluorescence settings (see Imaging Guidelines).

4

Imaging Guidelines

Imaging Guidelines

- Consult the Visium Spatial Gene Expression Imaging Guidelines Technical Note (CG000241) for complete guidelines.
- Before imaging, verify microscope settings using the Visium Imaging Test Slide (PN-2000235). Refer to Imaging System Recommendations in the Introduction for settings.

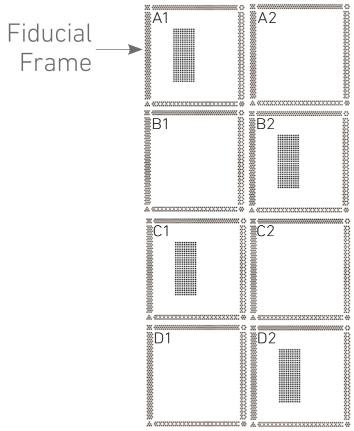

- The Visium Imaging Test Slide has eight areas surrounded by a fiducial frame. Four areas contain spots with fluorescent oligonucleotides (**A1, B2, C1, D2**)
- Fiducial frames should appear clear and in focus under brightfield settings. Fluorescent spots should appear clear and in focus under fluorescence settings.

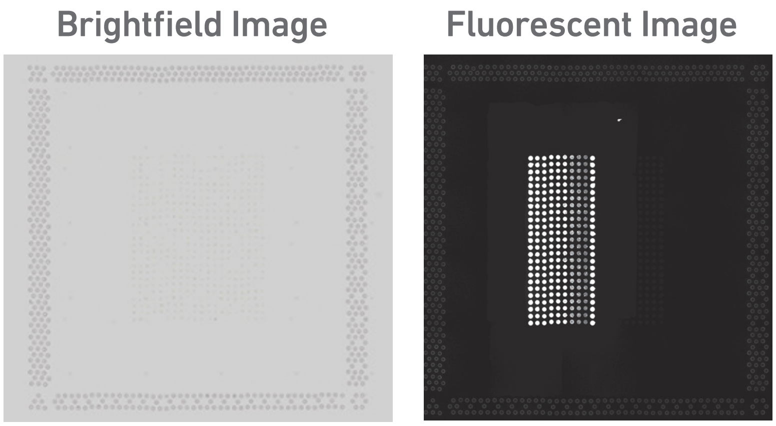

- The Visium Imaging Test Slide is also used to pre-define appropriate x and y-axis offsets before imaging Capture Areas.
- Once settings are verified, proceed with the Visium Spatial Tissue Optimization workflow.
- During brightfield and fluorescence imaging, image all eight Capture Areas at once without using autoexposure.
- A coverslip may be mounted on the slides to enhance optical quality. Although imaging without a coverslip is sufficient to visualize the tissue morphology, some imaging systems require the use of coverslips. See Appendix for Coverslip Application & Removal protocol.

- After fluorescence imaging, select the permeabilization time that results in the maximum fluorescence signal with the lowest signal diffusion. If the signal is the same at two time points, the longer permeabilization time is considered optimal. This assessment may be performed visually and does not require computational image analysis.
- Compare fluorescence images with brightfield images to ensure that a lack of fluorescence signal is due to insufficient permeabilization, not missing tissue.
- After selecting the optimum permeabilization condition, proceed to the Visium Spatial Gene Expression Reagent Kits User Guide (CG000239).

Imaging Examples

Example 1

- Panel A was imaged using a Nikon Eclipse Ti2 microscope with the following settings: TRITC filter cube, 200 ms exposure, 75% Sola pad.
- The negative control shows no fluorescence signal, while the positive control shows a strong signal. Low signal after **3-12 min** of permeabilization suggests insufficient permeabilization.
- **18 min** permeabilization time was chosen for the Visium Spatial Gene Expression workflow.

Mouse Brain

A

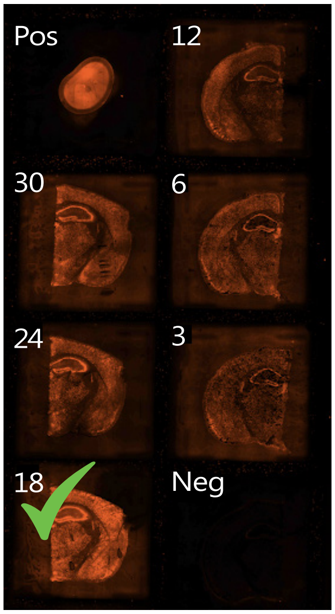

Example 2

- Panel B was imaged using a Nikon Eclipse Ti2 microscope with the following settings: TRITC filter cube, 300 ms exposure, 75% Sola pad.
- Although fluorescence signal is dim, this permeabilization time course is considered successful. Dim fluorescence signal is expected due to low RNA content.
- 12 min of permeabilization was selected for the Visium Spatial Gene Expression workflow.

Human Heart

B

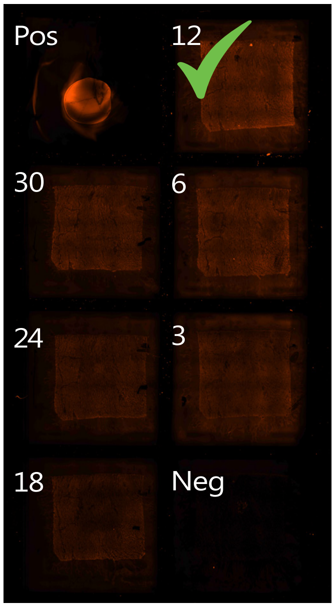

Troubleshooting

5

STEP	CORRECT	INCORRECT

2.1 After tissue permeabilization

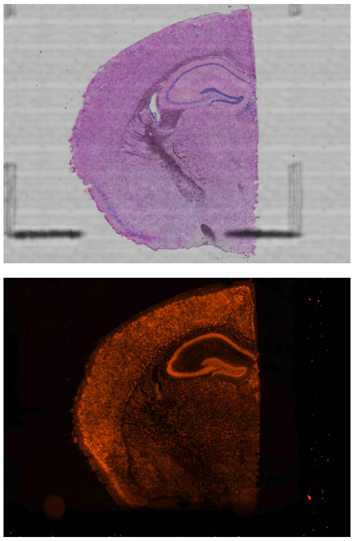

Tissue is fully permeabilized

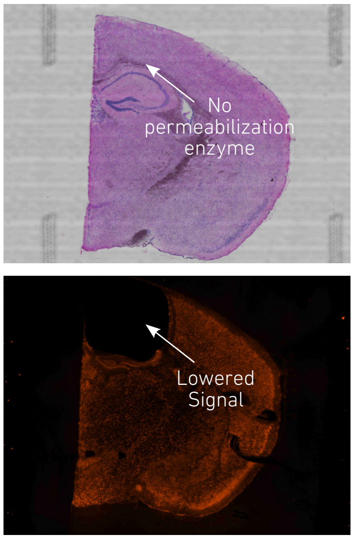

Tissue partially permeabilized

Uneven permeabilization may result in lowered fluorescence signal. Ensure that permeabilization reagents are applied to the tissue evenly at each step. A gentle tap may help spread the reagent more evenly.

3.1 Insufficient Slide Centrifugation after Tissue Removal

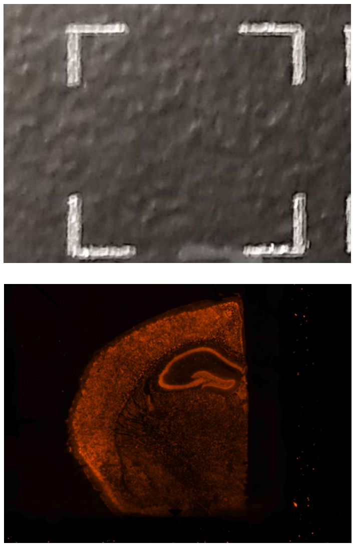

Sufficiently centrifuged slide

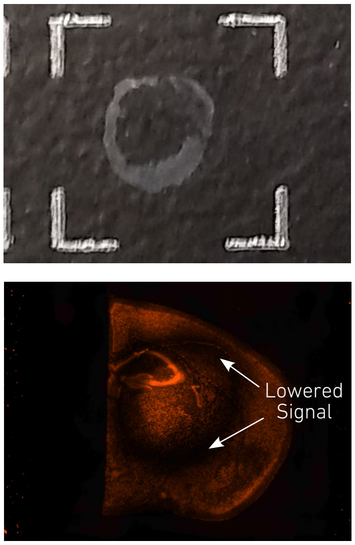

Insufficiently centrifuged slide

Insufficient slide centrifugation may result in deposits left on the slide, resulting in lowered fluorescence signal. Rinse slide 15x with 0.1X SSC and centrifuge for **30 sec** in a slide spinner or spin in a 50-ml centrifuge tube in a swinging bucket centrifuge at **250 rcf** for **30 sec**. Some deposits may cause permanent fluorescence signal loss and will not be recovered by additional rinsing and centrifuging.

STEP	NOTES
3.2<br>No Signal in RNA Positive Control	• Verify that RIN score of positive control ≥ 7. Low quality RNA will result in weak fluorescent signal.<br>• Verify that permeabilization enzyme was not added to the positive control well at step 2.1e.<br>• Verify that 0.1X SSC was not added to the positive control well at step 2.1h.<br>• Verify fluorescence imaging settings with the Visium Imaging Test Slide.
3.2<br>Tissue fluorescence cannot be distinguished from background	• Verify that RIN score of tissue block ≥ 7. Low quality RNA will result in weak fluorescent signal.<br>• Permeabilization time may have been insufficient. Repeat permeabilization time course with longer time increments.<br>• Tissue may not be compatible with methanol fixation.<br>• Verify fluorescence imaging settings with the Visium Spatial Test Slide.

Appendix

6

Coverslip Application & Removal

A coverslip may be mounted on the slides before imaging to enhance optical quality. Although imaging without a coverslip is sufficient to visualize the tissue morphology, some imaging systems require the use of coverslips.

If using a coverslip, follow this application and removal protocol to ensure that the tissue sections and the Capture Areas are not damaged.

Items
☐ **Large Coverslip** (Thermo Scientific 24 x 60 mm PN:22-050-233; Alternative, 24 x 50mm PN:22-050-232)
☐ **Milli-Q water (800 ml)**
☐ **80% Ethanol (50 ml)**
☐ **Laboratory Wipes**
☐ **Thermocycler Adaptor** (pre-equilibrated to **37°C** on a thermal cycler; may be used for drying)
☐ **Forceps**
☐ **85% Glycerol**<br>(prepare **30 ml** – add **25.5 ml** 100% glycerol and **4.5 ml** Milli-Q water to a 50-ml centrifuge tube and vortex. Wait for the bubbles to dissipate or centrifuge at **300 rcf** for **1 min** before use)

Application

Prior to mounting the coverslip, ensure that the sample and the slide with the tissue sections are dry. Moisture on the surface of the slide may dissolve the glycerol, resulting in faulty mounting.

If necessary, incubate the slide for **1 min** at **37°C** by placing on the pre-equilibrated Thermocycler Adaptor placed on a thermal cycler with the lid open.

i. Add **200 µl** 85% glycerol to cover the tissue sections on the slide uniformly. If necessary, hold the slide at an angle for uniform coverage.

ii. Apply the coverslip at an angle on one end of the slide. Slowly lower the coverslip, pressing down gently with forceps, without introducing bubbles.

iii. Remove excess glycerol by placing one long edge of the slide on a laboratory wipe, and gently tilt the slide back and forth. Repeat with the second long edge of the slide. Repeat the process until the coverslip is secured.

iv. After the coverslip is secured, **immediately** proceed with imaging.
DO NOT let the glycerol attached coverslip dry.
DO NOT use Cytoseal or nail polish for securing the coverslip.

Cover uniformly with glycerol

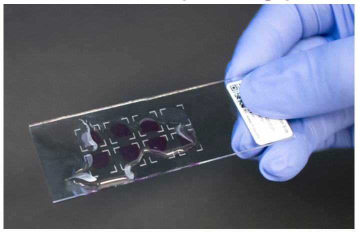

Apply coverslip

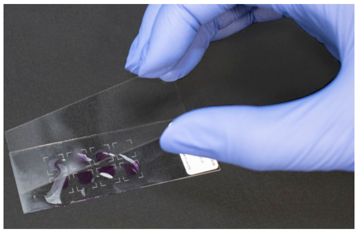

Press down

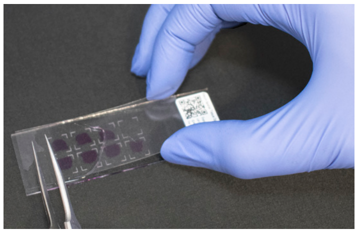

Remove excess glycerol

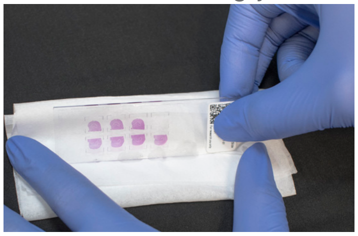

Coverslip Application & Removal

Removal

Remove the coverslip **immediately** after imaging is complete.

i. Dispense **800 ml** Milli-Q water in a beaker and **50 ml** 80% ethanol in a 50-ml centrifuge tube.

ii. Immerse the slide at ~45° angle in the water with the coverslipped surface fully submerged and facing down.

iii. Hold the slide in water until the coverslip slowly separates away from the slide. DO NOT move the slide up and down or shake forcibly to prevent damaging the tissue sections and the Capture Areas.

iv. Once the coverslip is detached, remove slide and immerse the slide at ~90° angle in the water 5x to remove any residual glycerol.

v. Immerse the slide in 80% ethanol dispensed in the centrifuge tube.

vi. Air dry slide at **room temperature**. If necessary, incubate the slide for **1 min** at **37°C** by placing on a pre-equilibrated Thermocycler Adaptor.

Proceed to the next workflow protocol step.

Immerse in water

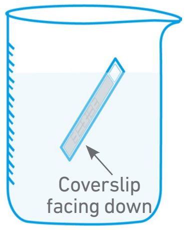

Hold in water

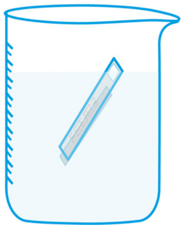

Coverslip detaches

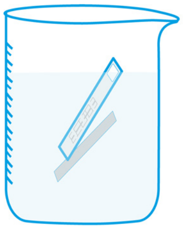

Supplement: Supplementary file 1 [file DataSheet_1.pdf]
