# Supplementary material for: MIF promotes cell invasion by the LRP1-uPAR interaction in pancreatic cancer cells
Source: Front Oncol. 2023 Jan 10;12:1028070. doi: 10.3389/fonc.2022.1028070 (PMC9871987; doi:10.3389/fonc.2022.1028070)
Supplement: Supplementary file 2 [file DataSheet_2.pdf]

CG000239 Rev A

USER GUIDE

Visium Spatial Gene Expression Reagent Kits

FOR USE WITH

Visium Spatial Gene Expression Slide & Reagent Kit, 16 rxns PN-1000184
Visium Spatial Gene Expression Slide & Reagent Kit, 4 rxns PN-1000187
Visium Accessory Kit, PN-1000194
Dual Index Kit TT Set A, 96 rxns PN-1000215

10x GENOMICS

Notices

Document Number

CG000239 • Rev A

Legal Notices

© 2019 10X Genomics, Inc (10x Genomics). All rights reserved. Duplication and/or reproduction of all or any portion of this document without the express written consent of 10x Genomics, is strictly forbidden. Nothing contained herein shall constitute any warranty, express or implied, as to the performance of any products described herein. Any and all warranties applicable to any products are set forth in the applicable terms and conditions of sale accompanying the purchase of such product. 10x Genomics provides no warranty and hereby disclaims any and all warranties as to the use of any third-party products or protocols described herein. The use of products described herein is subject to certain restrictions as set forth in the applicable terms and conditions of sale accompanying the purchase of such product. A non-exhaustive list of 10x Genomics' marks, many of which are registered in the United States and other countries can be viewed at: www.10xgenomics.com/trademarks. 10x Genomics may refer to the products or services offered by other companies by their brand name or company name solely for clarity, and does not claim any rights in those third party marks or names. 10x Genomics products may be covered by one or more of the patents as indicated at: www.10xgenomics.com/patents. The use of products described herein is subject to 10x Genomics Terms and Conditions of Sale, available at www.10xgenomics.com/legal-notices, or such other terms that have been agreed to in writing between 10x Genomics and user. All products and services described herein are intended FOR RESEARCH USE ONLY and NOT FOR USE IN DIAGNOSTIC PROCEDURES.

Licensed Software Updates Warranties

Updates to existing Licensed Software may be required to enable customers to use new or existing products.

Support

Email: support@10xgenomics.com

10x Genomics

6230 Stoneridge Mall Road

Pleasanton, CA 94588 USA

Document Revision Summary

**Document Number**	CG000239
**Title**	Visium Spatial Gene Expression Reagent Kits - User Guide
**Revision**	Rev A
**Revision Date**	November 2019

- This is Rev A of the User Guide.

Table of Contents

Introduction 6
Visium Spatial Gene Expression Reagent Kits 7
Visium Accessories 9
Recommended Thermal Cyclers 9
Recommended Real Time qPCR Systems 9
Imaging System Recommendations 10
Additional Kits, Reagents & Equipment 11
Protocol Steps & Timing 13
Stepwise Objectives 14

Tips & Best Practices 17

Sample Preparation Guidelines 29

Tissue Optimization Guidelines 31

Step 1 33
Tissue Staining & Imaging 34
1.1 Tissue Fixation 35
1.2 Tissue Staining 36
1.3 Imaging 37

Step 2 38
Permeabilization & Reverse Transcription 39
2.1 Tissue Permeabilization 40
2.2 Reverse Transcription 41

Step 3 42
Second Strand Synthesis 43
3.1 Second Strand Synthesis 44
3.2 Denaturation 45

Step 4 46
cDNA Amplification & QC 47
4.1 Cycle Number Determination – qPCR 48
4.2 cDNA Amplification 49
4.3 cDNA Cleanup – SPRIselect 50
4.4 cDNA QC & Quantification 51

Step 5 52
Visium Spatial Gene Expression Library Construction 53
5.1 Fragmentation, End Repair & A-tailing 55
5.2 Post Fragmentation, End Repair & A-tailing Double Sided Size Selection – SPRIselect 56
5.3 Adaptor Ligation 57
5.4 Post Ligation Cleanup – SPRIselect 58
5.5 Sample Index PCR 59
5.6 Post Sample Index PCR Double Sided Size Selection – SPRIselect 60
5.7 Post Library Construction QC 61
Sequencing 62
Troubleshooting 65
Appendix 69
Post Library Construction Quantification 70
Agilent TapeStation Traces 71
LabChip Traces 72
Coverslip Application & Removal 73
Oligonucleotide Sequences 74

Introduction

Visium Spatial Gene Expression Reagent Kits
Visium Accessories
Recommended Thermal Cyclers
Recommended Real Time qPCR Systems
Imaging System Recommendations
Additional Kits, Reagents & Equipment
Protocol Steps & Timing
Stepwise Objectives

Visium Spatial Gene Expression Reagent Kits

Visium Spatial Gene Expression Slide & Reagent Kit, 16 rxns PN-1000184

Visium Spatial Gene Expression Reagent Kit, 16 rxns PN-1000186 (store at –20°C)

Visium
Spatial Gene Expression
Reagent Kit

	#	PN
Permeabilization Enzyme	1	2000214
RT Reagent	1	2000086
RT Enzyme D	1	2000216
Template Switch Oligo	2	3000228
Reducing Agent B	1	2000087
Second Strand Primer	1	2000217
Second Strand Enzyme	1	2000218
Second Strand Reagent	1	2000219
cDNA Primers	1	2000089
Amp Mix	1	2000047

10xGenomics.com 10x GENOMICS

Library Construction Kit, 16 rxns PN-1000190 (store at –20°C)

Library Construction Kit

	#	PN
Fragmentation Buffer	1	2000091
Fragmentation Enzyme	1	2000090
Ligation Buffer	1	2000092
DNA Ligase	1	220110
Adaptor Oligos	1	2000094
Amp Mix	1	2000047

10xGenomics.com 10x GENOMICS

Visium Spatial Gene Expression Slide Kit, 16 rxns PN-1000185 (store at ambient temperature)

Visium
Spatial Gene Expression Slide Kit

	#	PN
Visium Spatial Gene Expression Slide	4	2000233
Slide Seal	20	3000279
Slide Cassette	4	3000406
Slide Gasket	4	3000426

10xGenomics.com 10x GENOMICS

Visium Spatial Gene Expression Reagent Kits

Visium Spatial Gene Expression Slide & Reagent Kit, 4 rxns PN-1000187

Visium Spatial Gene Expression Reagent Kit, 4 rxns PN-1000189 (store at –20°C)

Visium
Spatial Gene Expression Reagent Kit

	#	PN
Permeabilization Enzyme	1	2000214
RT Reagent	1	2000086
RT Enzyme D	1	2000227
Template Switch Oligo	1	3000228
Reducing Agent B	1	2000087
Second Strand Primer	1	2000217
Second Strand Enzyme	1	2000183
Second Strand Reagent	1	2000219
cDNA Primers	1	2000089
Amp Mix	1	2000103

10xGenomics.com

Library Construction Kit, 4 rxns PN-1000196 (store at –20°C)

Library Construction Kit

	#	PN
Fragmentation Buffer	1	2000091
Fragmentation Enzyme	1	2000104
Ligation Buffer	1	2000092
DNA Ligase	1	220131
Adaptor Oligos	1	2000094

10xGenomics.com

Visium Spatial Gene Expression Slide Kit, 4 rxns PN-1000188 (store at ambient temperature)

Visium
Spatial Gene Expression Slide Kit

	#	PN
Visium Spatial Gene Expression Slide	1	2000233
Slide Seals	5	3000279
Slide Cassette	1	3000406
Slide Gasket	1	3000426

10xGenomics.com

Dual Index Kit TT Set A, 96 rxns PN-1000215 (store at –20°C)

Dual Index Kit TT Set A

	#	PN
Dual Index Plate TT Set A	1	3000431

Visium Accessories

Product	Part Number (Kit)	Part Number (Item)
Thermocycler Adaptor	1000194	3000380
Visium Spatial Imaging Test Slide		2000235
10x Magnetic Separator		230003
Slide Alignment Tool		3000433

Recommended Thermal Cyclers

Supplier	Description	Part Number
Bio-Rad	C1000 Touch Thermal Cycler with 96-Deep Well Reaction Module	1851197
Eppendorf	MasterCycler Pro	North America 950030010<br>International 6321 000.019
Thermo Fisher Scientific	Veriti 96-Well Thermal Cycler	4375786

Recommended Real Time qPCR Systems

Supplier	Description	Part Number
Applied Biosystems	QuantStudio 12K Flex system	4471087
Bio-Rad	CFX96 Real-time System	1855096

Imaging System Recommendations

The imaging systems listed below were used by 10x Genomics. Any equivalent system with the listed features may be used for imaging. Hardware compatibility may be tested by using the Visium Spatial Imaging Test Slide.

Imaging Systems & Specifications	
**Microscopes** (Any equivalent system with the listed features may be used for imaging)	
Nikon	Nikon Eclipse Ti2 with brightfield and fluorescence capacity (TRITC)
Molecular Devices	ImageXpress Nano Automated Slide Imaging System
**Microscope Features**	
Objectives	• 4X (Plan APO λ; NA 0.20)<br>• 10X (Plan APO λ; NA 0.45)<br>• 20X (Plan APO λ; NA 0.75)
Scanning Stage	Microscope tile scanning functionality is required for imaging tissue sections placed on a Capture Area of a Visium Spatial slide.
Brightfield Features	• Color camera (3 x 8 bit, 2424 x 2424 pixel resolution)<br>• White balancing functionality<br>• Minimum Capture Resolution 2.18 µm/pixel<br>• Exposure times 2-10 milli sec
Fluorescence Features*	• Light source (or equivalent) with a wavelength range of 380-680 nm<br>• Monochrome camera (14 bit, 2,424 x 2,424 pixel resolution)<br>• Minimum Capture Resolution 2.18 µm/pixel<br>• Exposure times 100 milli sec-2 sec

* Only required for Visium Spatial Tissue Optimization protocol & Visium Spatial Imaging Test Slide verification

Additional Specifications	
Image Format	Save image in tiff (preferred) or jpeg format.
Computer	Computer with sufficient power to handle large images (0.5-5 GB)
Software	Image stitching software<br>(microscope's software or equivalent, like Image J)

Image Capture Guidelines:

The 8 mm x 8 mm area that includes the fiducial frame and the Capture Area with the tissue section should be represented by ≥2,000 x 2,000 pixel portion of the image. When setting the microscope for imaging individual Capture Area, the imaging area should be ~1-2 mm beyond the fiducial frame for optimal imaging alignment. Minimize imaging of any adjacent Capture Area/s when taking images of a specific Capture Area with a tissue section. For lossy compression, such as jpeg, the quality level should be kept high enough to represent the fiducial frame crisply and without artifact.

Additional Kits, Reagents & Equipment

The items in the table below have been validated by 10x Genomics and are highly recommended for the Visium Spatial Reagent Kits protocol. Substituting materials may adversely affect system performance. This list does not include standard laboratory equipment such as water baths, centrifuges, vortex mixers, pH meters, freezers etc.

Supplier	Description	Part Number (US)
**Plastics**		
Eppendorf	PCR Tubes 0.2 ml 8-tube strips<br>DNA LoBind Tubes, 1.5 ml<br>DNA LoBind Tubes, 2.0 ml (when processing more than 2 slides)	951010022<br>022431021<br>022431048
USA Scientific	TempAssure PCR 8-tube strip	1402-4700
Thermo Fisher Scientific	MicroAmp 8-Tube Strip, 0.2 ml<br>MicroAmp 8 -Cap Strip, clear<br>Simport Scientific LockMailer Tamper Evidence Slide Mailer<br>(alternatively, use a 50-ml centrifuge tube)	N8010580<br>N8010535<br>22-038-399
Corning	Self-Standing Polypropylene Centrifuge Tubes (50 ml), sterile<br>Corning 250 mL Vacuum System, 0.2 µm Pore 19.6cm^2 NY Membrane	430921<br>430771
Bio-Rad	Hard-shell PCR Plates 96-well, thin wall (pkg of 50)<br>(alternatively, use any compatible PCR Plate)<br>Microseal 'B' PCR Plate Sealing Film, adhesive<br>(alternatively, use any PCR Plate sealing adhesive)	HSP9665<br><br>MSB1001
Rainin	Tips LTS 200UL Filter RT-L200FLR<br>Tips LTS 1ML Filter RT-L1000FLR<br>Tips LTS 20UL Filter RT-L10FLR	30389240<br>30389213<br>30389226
VWR	Divided Polystyrene Reservoirs	41428-958
**Kits & Reagents**		
Agilent	Hematoxylin, Mayer's (Lillie's Modification)<br>(alternatively, Mayer's Hematoxylin from Electron Microscopy Services, 2638102 may be used)<br>Bluing Buffer, Dako	S330930-2<br><br>CS70230-2
Thermo Fisher Scientific	Nuclease-free Water<br>Low TE Buffer (10 mM Tris-HCl pH 8.0, 0.1 mM EDTA)<br>Tris Base (White Crystals or Crystalline Powder/Molecular Biology)<br>Tris 1M, pH 7.0, RNase-free	AM9937<br>12090-015<br>BP152-500<br>AM9850G
Fisher Chemical	Hydrochloric Acid Solution, 0.1N	SA54-1
KAPA Biosystems	KAPA SYBR FAST qPCR Master Mix (2X)	KK4600
Millipore Sigma	Ethanol, Pure (200 Proof, anhydrous)<br>Potassium Hydroxide Solution, 8M<br>Methanol, for HPLC, ≥ 99.9%<br>2-Propanol (Isopropanol), ≥ 99.5%<br>SSC Buffer 20X Concentrate<br>Eosin Y solution, aqueous, 0.5% (w/v) in water<br>Acetic acid, ≥ 99.9%	E7023-500ML<br>P4494-50ML<br>34860<br>I9516-25ML<br>S66391L<br>HT110216-500ML<br>A6283
Beckman Coulter	SPRIselect Reagent Kit	B23318
Qiagen	Qiagen Buffer EB	19086
-	Ultrapure/Milli-Q water (from Milli-Q Integral Ultrapure Water System or equivalent)	

Choose either Eppendorf, USA Scientific or Thermo Fisher Scientific PCR 8-tube strips.

Additional Kits, Reagents & Equipment

The items in the table below have been validated by 10x Genomics and are highly recommended for the Visium Spatial Reagent Kits protocol. Substituting materials may adversely affect system performance. This list does not include standard laboratory equipment such as water baths, centrifuges, vortex mixers, pH meters, freezers etc.

Supplier	Description	Part Number (US)
**Equipment**		
Rainin	Pipet-Lite Multi Pipette L8-200XLS+<br>Pipet-Lite LTS Pipette L-2XLS+<br>Pipet-Lite LTS Pipette L-10XLS+<br>Pipet-Lite LTS Pipette L-20XLS+<br>Pipet-Lite LTS Pipette L-100XLS+<br>Pipet-Lite LTS Pipette L-200XLS+<br>Pipet-Lite LTS Pipette L-1000XLS+	17013805<br>17014393<br>17014388<br>17014392<br>17014384<br>17014391<br>17014382
Thermo Fisher Scientific	MYFUGE 12 Mini Centrifuge<br>(alternatively, use any equivalent mini centrifuge)	C1012
**Quantification & Quality Control**		
Agilent	2100 Bioanalyzer Laptop Bundle<br>High Sensitivity DNA Kit<br>4200 TapeStation<br>High Sensitivity D1000 ScreenTape/Reagents<br>High Sensitivity D5000 ScreenTape/Reagents	G2943CA<br>5067-4626<br>G2991AA<br>5067-5592/ 5067-5593<br>5067-5584/ 5067-5585
PerkinElmer	LabChip GX Touch HT Nucleic Acid Analyzer<br>DNA High Sensitivity Reagent Kit	CLS137031<br>CLS760672
Advanced Analytical	Fragment Analyzer Automated CE System - 12 cap<br>Fragment Analyzer Automated CE System - 48/96 cap<br>High Sensitivity NGS Fragment Analysis Kit	FSv2-CE2F<br>FSv2-CE10F<br>DNF-474
KAPA Biosystems	KAPA Library Quantification Kit for Illumina Platforms	KK4824

Choose Bioanalyzer, TapeStation, Lab Chip or Fragment Analyzer based on availability & preference.

Protocol Steps & Timing

8 h*

Steps		Timing	Stop & Store
**Step 1 – Tissue Staining & Imaging**			
1.1	Tissue Fixation	35 min	
1.2	Tissue Staining	30 min	
1.3	Tissue Imaging*	Variable	
**Step 2 – cDNA Synthesis**			
2.1	Tissue Permeabilization	Variable	
2.2	Reverse Transcription	65 min	
**Step 3 – Second Strand Synthesis & Denaturation**			
3.1	Second Strand Synthesis	25 min	
3.2	cDNA Denaturation	15 min	
**Step 4 – cDNA Amplification & QC**			
4.1	Cycle Number Determination – qPCR	45 min	
4.2	cDNA Amplification	45-60 min	STOP 4°C ≤72 h or –20°C ≤1 week
4.3	cDNA Cleanup – SPRIselect	20 min	STOP 4°C ≤72 h –20°C ≤4 weeks
4.4	cDNA QC & Quantification*	50 min	
**Step 5 – Visium Spatial Gene Expression Library Construction**			
5.1	Fragmentation, End Repair & A-tailing	50 min	
5.2	Post Fragmentation, End Repair & A-tailing Double Sided Size Selection – SPRIselect	30 min	
5.3	Adaptor Ligation	25 min	
5.4	Post Ligation Cleanup- SPRIselect	20 min	
5.5	Sample Index PCR	40 min	STOP 4°C ≤72 h
5.6	Post Sample Index PCR Double Sided Size Selection- SPRIselect	30 min	STOP 4°C ≤72 h or -20°C long term
5.7	Post Library Construction QC*	50 min	

*~8 h workflow, excluding imaging & QC steps

Stepwise Objectives

The Visium Spatial Gene Expression Solution measures total mRNA in intact tissue sections and maps the location(s) where gene activity is occurring. Each Visium Spatial Gene Expression Slide contains Capture Areas with gene expression spots that include primers required for capture and priming of poly-adenylated mRNA. Tissue sections placed on these Capture Areas are permeabilized and cellular mRNA is captured by the primers on the gene expression spots. All the cDNA generated from mRNA captured by primers on a specific spot share a common Spatial Barcode. Libraries are generated from the cDNA and sequenced and the Spatial Barcodes are used to associate the reads back to the tissue section images for spatial gene expression mapping.

This document outlines the protocol for generating Visium Spatial Single Cell 3' Gene Expression libraries from tissue sections placed on the Capture Areas of a Visium Spatial Gene Expression Slide.

Visium Spatial Gene Expression Slide

The Visium Spatial Gene Expression Slide includes 4 Capture Areas (6.5 x 6.5 mm), each defined by a fiducial frame (fiducial frame + Capture Area is 8 x 8 mm). The Capture Area has ~5,000 gene expression spots, each spot with primers that include:

- Illumina TruSeq Read 1 (partial read 1 sequencing primer)
- 16 nt Spatial Barcode (all primers in a specific spot share the same Spatial Barcode)
- 12 nt unique molecular identifier (UMI)
- 30 nt poly(dT) sequence (captures poly-adenylated mRNA for cDNA synthesis).

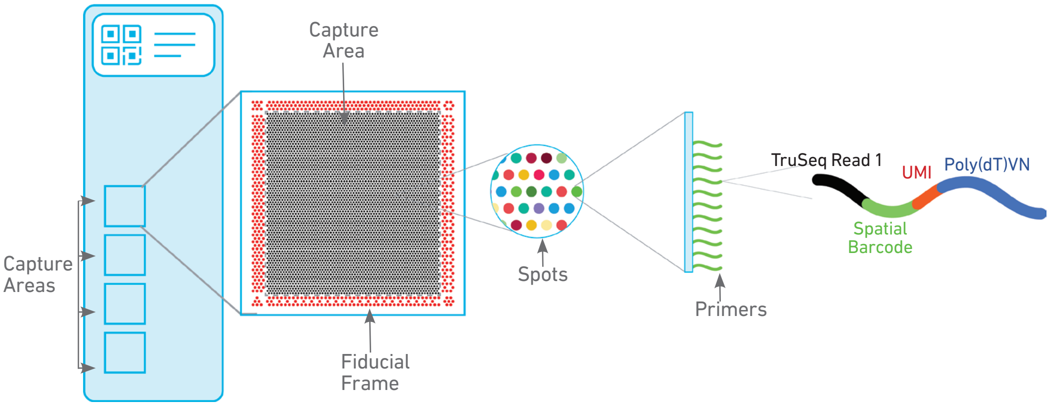

Step 1
Tissue Staining & Imaging

Tissue sections on the Capture Areas of the Visium Spatial Gene Expression are fixed using methanol. Hematoxylin is used to stain the nuclei, followed by eosin staining for the extracellular matrix and cytoplasm. The stained tissue sections are imaged. The images will be used downstream to map the gene expression patterns back to the tissue sections.

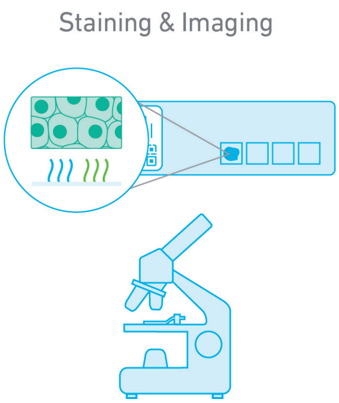

Step 2
Permeabilization & Reverse Transcription

A Permeabilization Enzyme is used to permeabilize the tissue sections on the slide. The poly-adenylated mRNA released from the overlying cells is captured by the primers on the spots. RT Master Mix containing reverse transcription reagents is added to the permeabilized tissue sections. Incubation with the reagents produces spatially barcoded, full-length cDNA from poly-adenylated mRNA on the slide.

Permeabilization

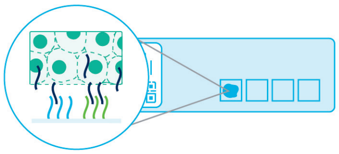

Reactions on slide Capture Areas

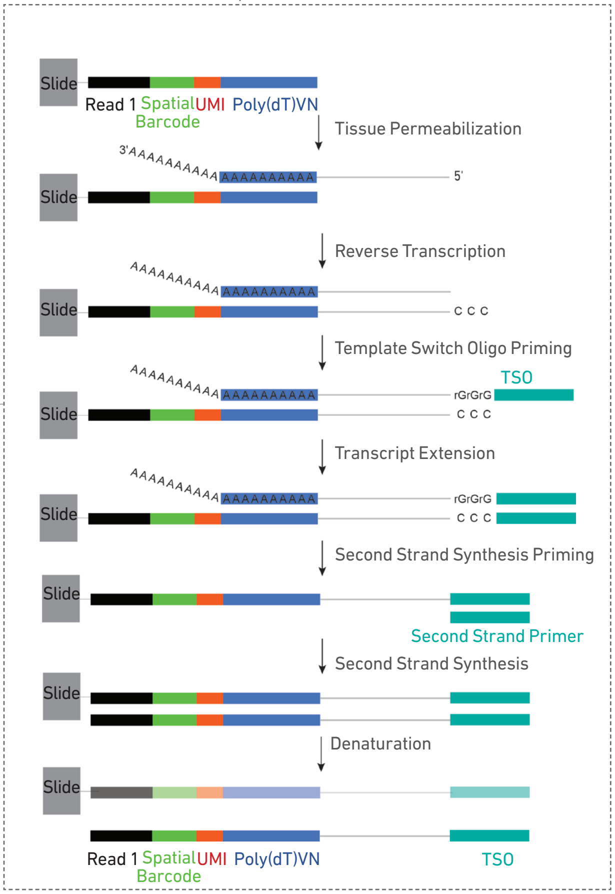

Step 3
Second Strand Synthesis & Denaturation

Second Strand Mix is added to the tissue sections on the slide to initiate second strand synthesis. This is followed by denaturation and transfer of the cDNA from each Capture Area to a corresponding tube for amplification and library construction.

Step 4
cDNA Amplification & QC

After transfer of cDNA from the slide, spatially barcoded, full-length cDNA is amplified via PCR to generate sufficient mass for library construction.

cDNA amplification

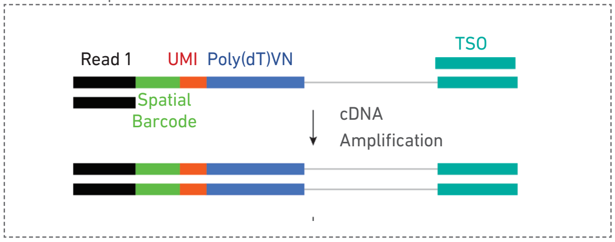

Step 5 Visium Spatial Gene Expression Library Construction

Enzymatic fragmentation and size selection are used to optimize the cDNA amplicon size. P5, P7, i7 and i5 sample indexes, and TruSeq Read 2 (read 2 primer sequence) are added via End Repair, A-tailing, Adaptor Ligation, and PCR. The final libraries contain the P5 and P7 primers used in Illumina amplification.

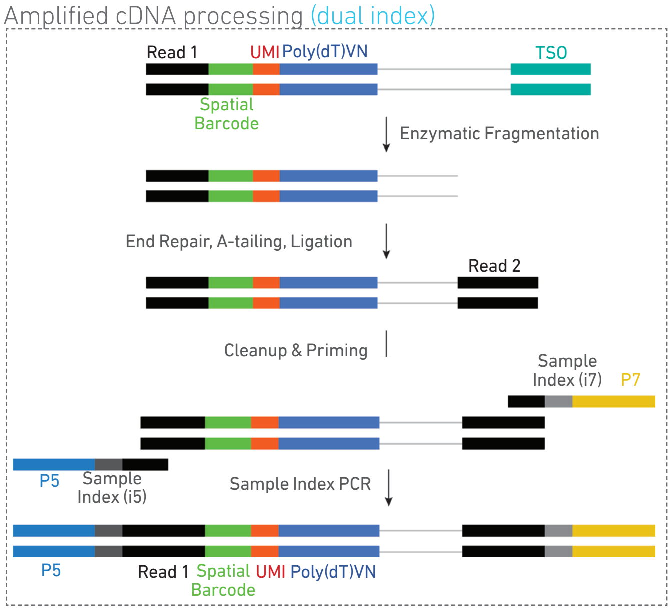

Step 6 Sequencing

A Visium Spatial Gene Expression library comprises standard Illumina paired-end constructs which begin and end with P5 and P7. The 16 bp Spatial Barcode and 12 bp UMI are encoded in Read 1, while Read 2 is used to sequence the cDNA fragment. i7 and i5 sample index sequences are incorporated. TruSeq Read 1 and TruSeq Read 2 are standard Illumina sequencing primer sites used in paired-end sequencing.

Illumina sequencer compatibility, sample indices, library loading and pooling for sequencing are summarized in step 6.

Visium Spatial Gene Expression Library

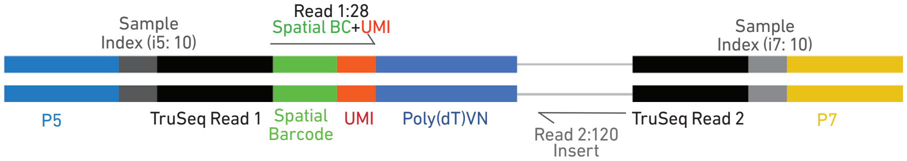

See Appendix for Oligonucleotide Sequences

Tips & Best Practices

TIPS

Icons

Tips & Best Practices section includes additional guidance	Signifies critical step requiring accurate execution	Troubleshooting section includes additional guidance

General Reagent Handling

- Fully thaw and thoroughly mix reagents before use.
- Keep all enzymes and Master Mixes on ice during setup and use. Promptly move reagents back to the recommended storage.
- Use a pH meter to adjust pH as necessary during buffer preparation.

Pipette Calibration

- Follow manufacturer's calibration and maintenance schedules.
- Pipette accuracy is particularly important when using SPRIselect reagents.

Visium Spatial Gene Expression Slide

- Includes 4 Capture Areas (6.5 x 6.5 mm), each with ~5,000 unique gene expression spots.
- Each gene expression spot includes primers with a unique Spatial Barcode (see Stepwise Objectives for additional information).
- The active surface of the slide is defined by a readable label that includes the serial number.
- The tissue sections are always placed on the active surface of the Capture Areas. For more information, consult the Visium Spatial Protocols – Tissue Preparation Guide (Demonstrated Protocol CG000240).

Visium Spatial Gene Expression Slide

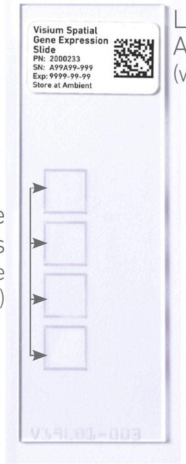

Note the serial number on the slide label; will be required for downstream analysis.

Slide Storage

- Always store slides in a cool, dry environment.
- Store unused slides in original packaging and keep sealed. DO NOT remove dessicant. If necessary, place the sealed container in a secondary container, such as a resealable bag.
- After tissue placement, store the slides at **–80°C** in a sealed container.

Slide Storage

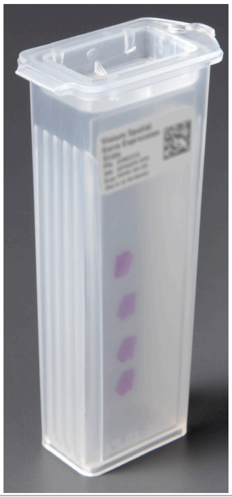

Slide Handling

- Always wear gloves when handling slides.
- Ensure that the active surface of a slide faces up and is never touched. The orientation of the label on the slide defines the active surface.
- The tissue sections should always be on the active surface of the slide. DO NOT touch the tissue sections.
- Minimize exposure of the slides to sources of particles and fibers.
- When immersing slides in water, ensure that the tissue sections are completely submerged.
- Keep the slide flat on a clean work surface when adding reagents to the active surface.
- Ensure that no absorbent surface is in contact with the reagents on the slide during incubation.

Active Surface with Tissue Sections

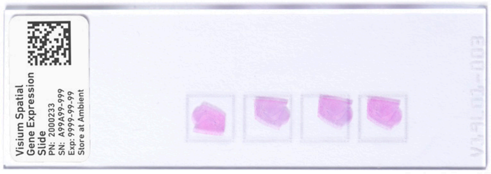

Immersing Slide

Correct Incorrect

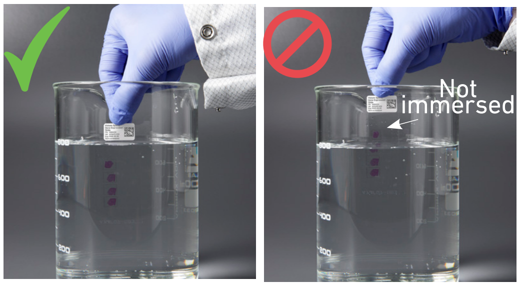

Reagent on Slide

Correct Incorrect

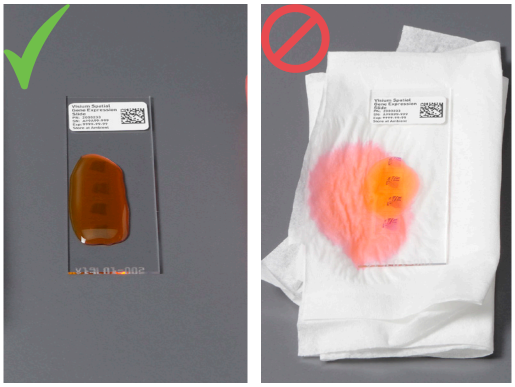

Slide Cassette

- The Slide Cassette encases the slide and creates leakproof wells for adding reagents.
- Place the slides in the Slide Cassette only when specified.
- The Slide Cassette is disposable and intended for one-time use.
- An Insert Clip and four tabs at the back of the Slide Cassette are used for holding the slide in the cassette, as shown.
- The cassette includes a removable gasket (disposable; one-time use) corresponding to the Capture Areas on the slides.
- The Slide Cassette may be assembled using the Slide Alignment Tool or manually. Instructions for both are provided in the following section.
- See Slide Cassette Assembly & Removal instructions for details.
- Ensure that the back of the Slide Cassette is facing the user prior to assembly. The active surface of the slide with tissue sections will face down such that the slide label is no longer readable.
- Practice assembly with a plain glass slide.

Slide Cassette

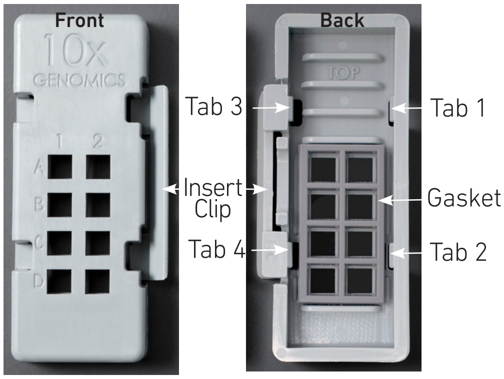

Slide Alignment Tool

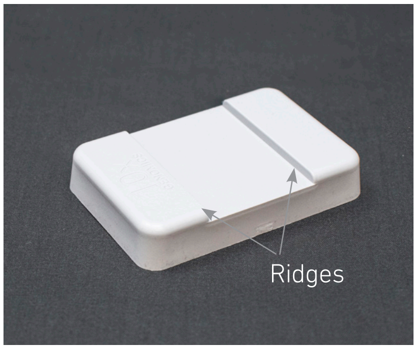

Slide Cassette Assembly

Position Slide Cassette along alignment tool ridges

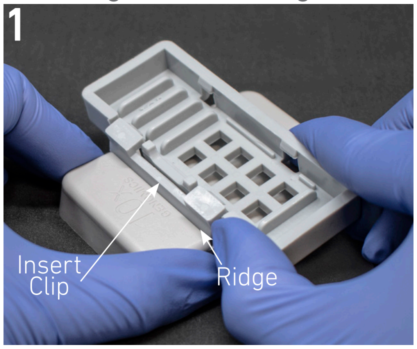

Push Insert Clip along the ridge & press Slide Cassette down

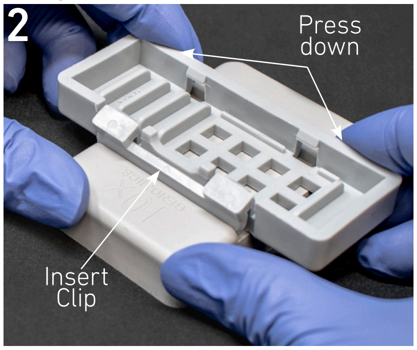

Slide Cassette secured on alignment tool

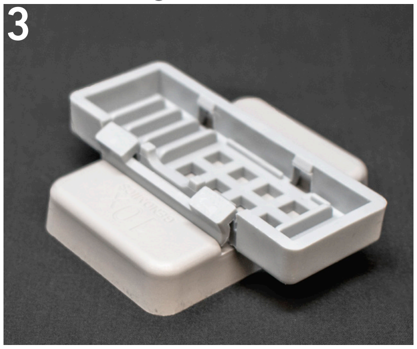

Position Gasket to align with Slide Cassette cutouts

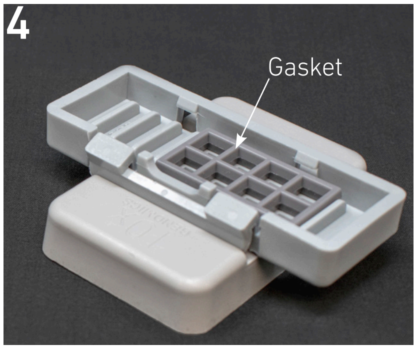

Slide insertion may push gasket out of alignment with slide cutouts. Adjust if necessary.

Insert long edge of slide under tabs 1 & 2; ensure slide is flush

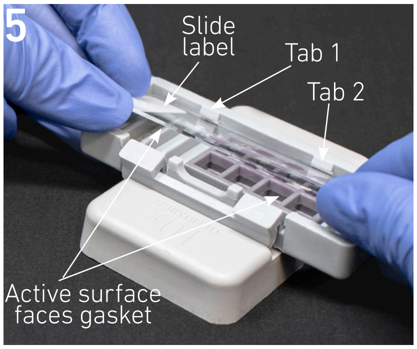

Press slide down until it is flush with the gasket and under tabs 3 & 4

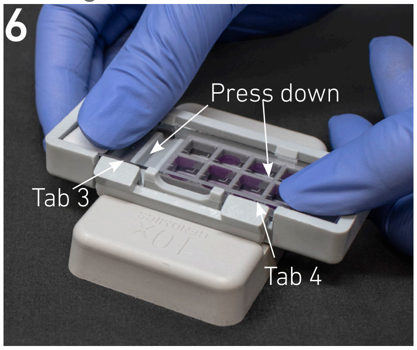

Remove Slide Cassette while pressing slide against the gasket

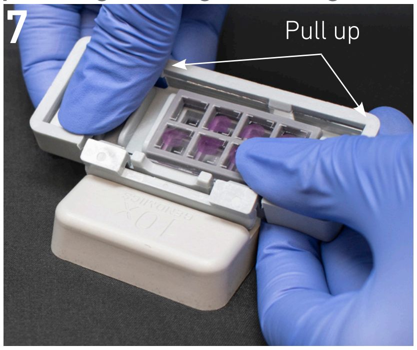

Slide Cassette Removal*

Position Slide Cassette along alignment tool ridges

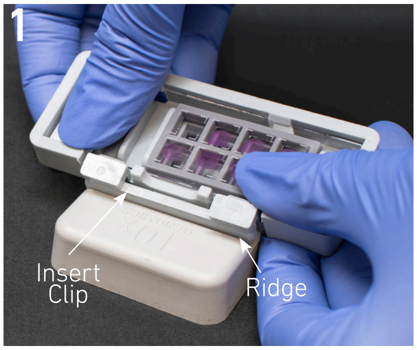

Push Insert Clip along the ridge & press down

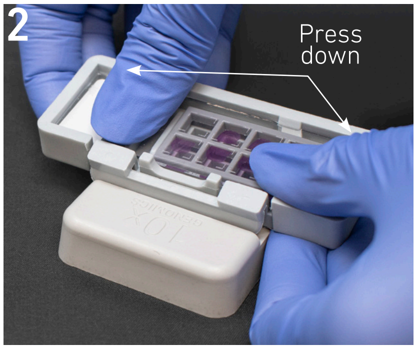

Slide Cassette Sits securely on alignment tool

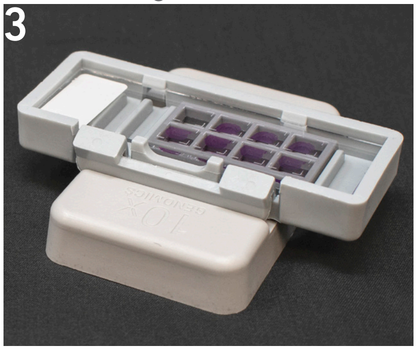

Lift slide at Slide Cassette groove

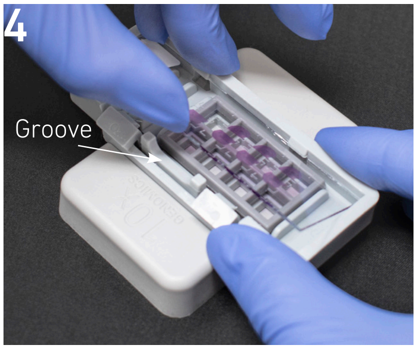

*Slide removal not needed for the Visium Spatial Gene Expression protocol.

Manual Slide Cassette Assembly & Removal

Assembly

i. Insert the gasket and align the gasket and Slide Cassette cutouts.

ii. Align the label on top of the slide to the top of the Slide Cassette, as shown.

iii. Insert the slide under tabs 1 and 2. Ensure that the long edge of the slide is flush with the side of the Slide Cassette.

iv. Press the insert clip **very firmly** by applying even force on the lower part of the insert clip.

v. Press down on the slide with a finger in between tabs 3 and 4 until the slide is under each tab and release the insert clip.

Removal*

i. Press the insert clip **very firmly** to release the slide from the cassette.

ii. Lift slide at Slide Cassette groove between tabs 3 and 4 until the slide can be removed.

*Slide removal not needed for the Visium Spatial Gene Expression protocol.

Slide Cassette

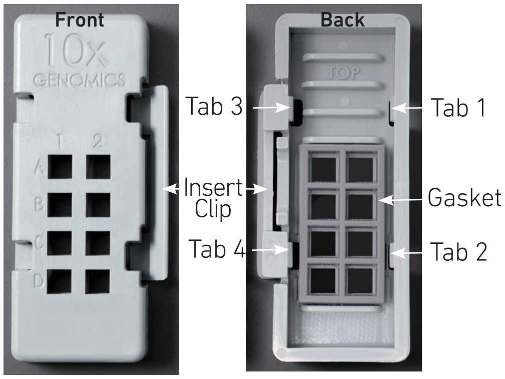

Slide Cassette Assembly

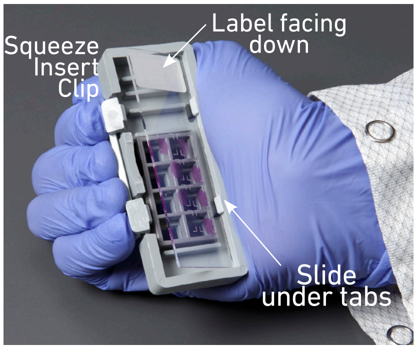

Insert Clip - Press Firmly

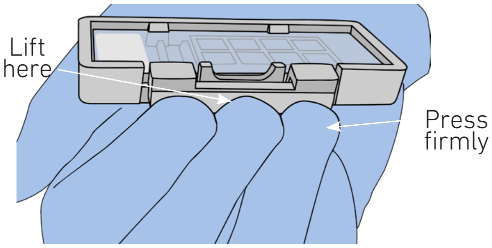

Reagent Addition & Removal from Wells

- Place the assembled slide in the Slide Cassette flat on a clean work surface.
- Dispense and remove reagents along the side of the wells without touching the tissue sections and without introducing bubbles.
- Always cover the tissue section completely when adding reagents to the well. A gentle tap may help spread the reagent more evenly.
- Ensure that no bubbles are introduced in the process.

Reagent Addition/Removal

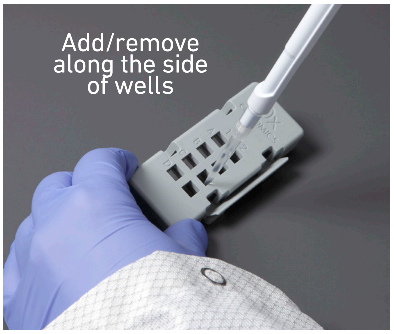

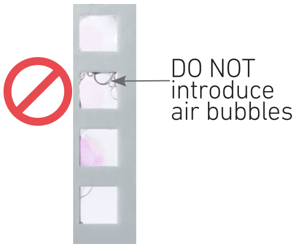

Slide Seal Application & Removal

Application

- Place the Slide Cassette flat on a clean work surface.
- Remove the back of the adhesive Slide Seal.
- Align the Slide Seal with the surface of the Slide Cassette and apply while firmly holding the Slide Cassette with one hand.
- Press on the Slide Seal to ensure uniform adhesion.

Removal

- Place the Slide Cassette flat on a clean work surface.
- Pull on the Slide Seal from the edge while firmly holding the Slide Cassette. Ensure that no liquid splashes out of the wells.

Slide Seal Application

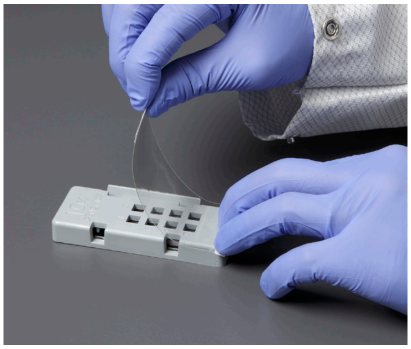

Slide Incubation Guidance

Incubation at a specified temperature

- Position a Thermocycler Adaptor on a thermal cycler that is set at the incubation temperature.
- Ensure that the Thermocycler Adaptor is in contact with the thermal cycler surface uniformly.
- When incubating a slide, position the slide on the Thermocycler Adaptor with the active surface facing up.
- Ensure that the entire bottom surface of the slide is in contact with Thermocycler Adaptor.
- When incubating a slide encased in a Slide Cassette, place the assembled unit on the Thermocycler Adaptor with the wells facing up. The Slide Cassette should always be sealed when on the Thermocycler Adaptor.

Place Thermocycler Adaptor

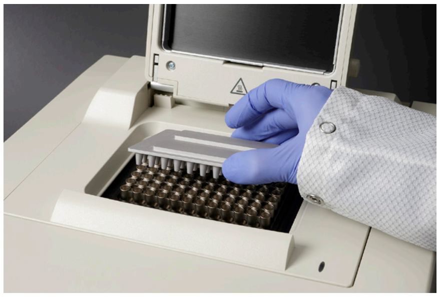

Incubate Slide

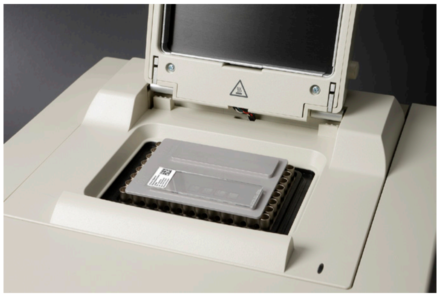

Incubate Assembled Slide Cassette

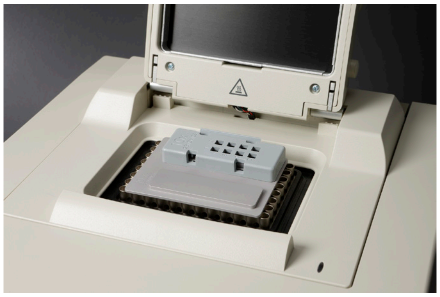

Incubation at room temperature

- Place the slide/Slide Cassette on a flat, clean work surface.
- Ensure that no absorbent surface is in contact with the reagents on the slide during incubation.

Slide Incubation

Correct Incorrect

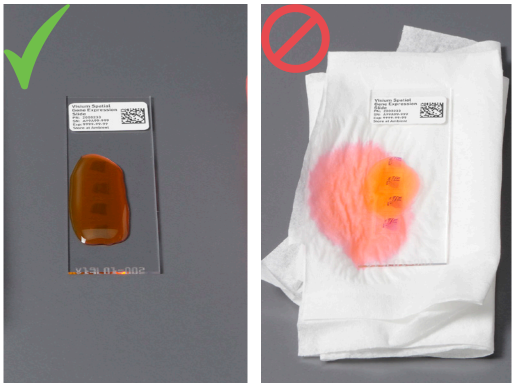

10x Magnetic Separator

- Offers two positions of the magnets (high and low) relative to a tube, depending on its orientation. Flip the magnetic separator over to switch between high (magnet•**High**) or low (magnet•**Low**) positions.
- If using MicroAmp 8-Tube Strips, use the high position (magnet•**High**) only throughout the protocol.

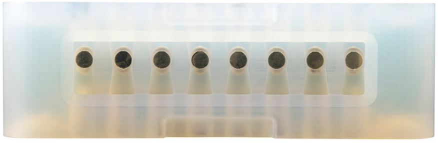

Magnetic Bead Cleanup Steps

- During magnetic bead based cleanup steps that specify waiting "until the solution clears", visually confirm clearing of solution before proceeding to the next step. See adjacent panel for an example.
- The time needed for the solution to clear may vary based on specific step, reagents, volume of reagents etc.

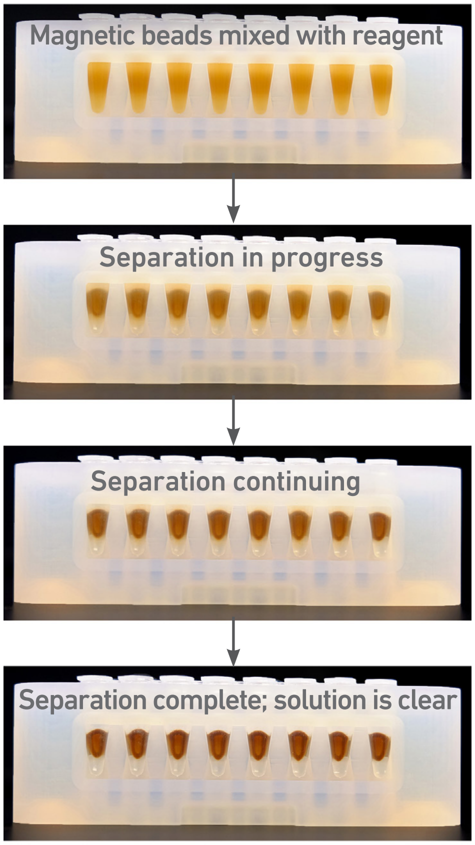

SPRIselect Cleanup & Size Selection

- After aspirating the desired volume of SPRIselect reagent, examine the pipette tips before dispensing to ensure the correct volume is transferred.
- Pipette mix thoroughly as insufficient mixing of sample and SPRIselect reagent will lead to inconsistent results.
- Use fresh preparations of 80% Ethanol.

Tutorial — SPRIselect Reagent:DNA Sample Ratios

SPRI beads selectively bind DNA according to the ratio of SPRIselect reagent (beads).

$$\text{Example: Ratio} = \frac{\text{Volume of SPRIselect reagent added to the sample}}{\text{Volume of DNA sample}} = \frac{50\ \mu l}{100\ \mu l} = \mathbf{0.5X}$$

Schematic of Double Sided Size Selection

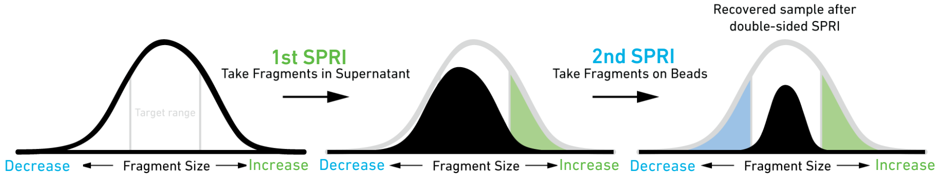

After the first SPRI, supernatant is transferred for a second SPRI while larger fragments are discarded (green). After the second SPRI, fragments on beads are eluted and kept while smaller fragments are discarded (blue). Final sample has a tight fragment size distribution with reduced overall amount (black).

Tutorial — Double Sided Size Selection

Step a – First SPRIselect: Add **50 µl** SPRIselect reagent to **100 µl** sample (**0.5X**).

$$\text{Ratio} = \frac{\text{Volume of SPRIselect reagent added to the sample}}{\text{Volume of DNA sample}} = \frac{50\ \mu l}{100\ \mu l} = \mathbf{0.5X}$$

Step b – Second SPRIselect: Add **30 µl** SPRIselect reagent to supernatant from step a (**0.8X**).

$$\text{Ratio} = \frac{\text{Total Volume of SPRIselect reagent added to the sample (step a + b)}}{\text{Original Volume of DNA sample}} = \frac{50\ \mu l + 30\ \mu l}{100\ \mu l} = \mathbf{0.8X}$$

Enzymatic Fragmentation

- Ensure enzymatic fragmentation reactions are prepared on ice and then loaded into a thermal cycler pre-cooled to **4°C** prior to initiating the Fragmentation, End Repair, and A-tailing incubation steps.

Sample Indices (i5/i7) in Sample Index PCR

- Choose the appropriate sample index sets to ensure that no sample indices overlap in a multiplexed sequencing run.
- Each well in the Dual Index Plate TT Set A contains a unique i7 and a unique i5 oligonucleotide.

Index Hopping Mitigation

Index hopping can impact pooled samples sequenced on Illumina sequencing platforms that utilize patterned flow cells and exclusion amplification chemistry. To minimize index hopping, follow the guidelines listed below.

- Remove adapters during cleanup steps.
- Ensure no leftover primers and/or adapters are present when performing post-Library Construction QC.
- Store each library individually at **4°C** for up to **72 h** or at **–20°C** for **long-term** storage. DO NOT pool libraries during storage.
- Pool libraries prior to sequencing. An additional 1.0X SPRI may be performed for the pooled libraries to remove any free adapters before sequencing.
- Hopped indices can be computationally removed from the data generated from Visium Spatial Gene Expression dual index libraries.

Sample Preparation Guidelines

Sample Preparation Guidelines

Proper tissue handling and preparation techniques are critical in preserving the morphological quality of the tissue sections and subsequent transcript profiling using Visium Spatial protocols.

Listed below are some key considerations for preparing samples that are compatible with the Visium Spatial protocols.
Consult the Visium Spatial Protocols – Tissue Preparation Guide for complete information (Demonstrated Protocol CG000240).

Key Considerations

Slide Handling (before sectioning)

☐ Equilibrate Visium slides to cryostat temperature before cryosectioning.

☐ Store unused slides in original packaging and keep sealed. DO NOT remove desiccant. If necessary, store original packaging in a secondary container such as a resealable bag.

Freezing and Embedding

☐ Snap freeze samples in a bath of isopentane and liquid nitrogen.

☐ Store frozen samples at **-80°C** in a sealed container for **long-term** storage prior to embedding.

Cryosectioning

☐ Equilibrate OCT tissue block to the cryostat chamber temperature for **30 min**.

☐ Place tissue sections on the Capture Area within the fiducial frame on the slide.

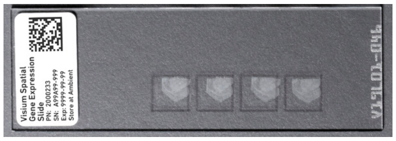

Slide Handling (after sectioning)

☐ Maintain slides containing sections in a low moisture environment.

☐ Keep slides cold and transport slides on dry ice.

☐ DO NOT leave slides at room temperature.

Sample Storage

☐ Store slides individually in a sealed container at **-80°C** for up to **a week** to avoid multiple freeze thaw cycles. If necessary, place the sealed container in a secondary container, such as a resealable bag.

Tissue Optimization Guidelines

Tissue Optimization Guidelines

Prior to using a new tissue type for generating Visium Spatial Gene Expression libraries, the permeabilization time should be optimized. Failure to optimize the permeabilization time can diminish the efficiency of the assay significantly.

Refer to the Visium Spatial Gene Expression Reagent Kits – Tissue Optimization User Guide (CG000238) for the complete protocol for optimizing permeabilization time for any tissue of interest.

Briefly, the Visium Spatial Tissue Optimization workflow includes placing tissue sections on 7 Capture Areas on a Visium Tissue Optimization slide. The sections are fixed, stained, and then permeabilized for different times. mRNA released during permeabilization binds to oligonucleotides on the Capture Areas. Fluorescent cDNA is synthesized on the slide and imaged. The permeabilization time that results in maximum fluorescence signal with the lowest signal diffusion is optimal. If the signal is the same at two time points, the longer permeabilization time is considered optimal.

Tissue Optimization Workflow

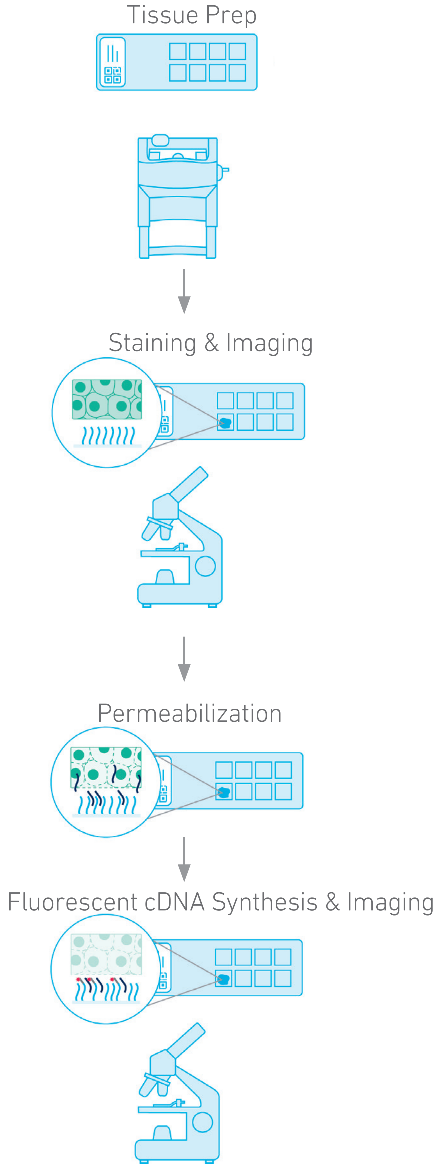

Choose the permeabilization time that results in maximum fluorescence signal with the lowest signal diffusion. If the signal is the same at two time points, choose the longer permeabilization time.

Example: Tissue Permeabilization Time Course

Mouse brain sections were imaged after permeabilization and fluorescent cDNA synthesis, using a Nikon Eclipse Ti2 microscope.

- Positive control: Strong fluorescent signal.
- Negative Control: No fluorescent signal.
- Optimal signal: **18 min**.
  Use for Visium Spatial Gene Expression protocol.

Permeabilization Time Course (min)

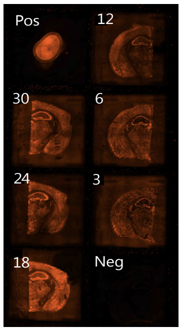

Step 1

Tissue Staining & Imaging

1.1 Tissue Fixation

1.2 Tissue Staining

1.3 Tissue Imaging

1

1.0 Tissue Staining & Imaging

Ensure that microscope settings have been optimized to capture brightfield images.

Consult the Visium Spatial Gene Expression Imaging Guidelines Technical Note (CG000241) for more information.

CHECKLIST – GET STARTED!

	Items	10x PN	Preparation & Handling	Storage
**Place at -20°C**				
☐	**Methanol**<br>Dispense 40 ml/slide* in a 50-ml centrifuge tube	-	Chill to **-20°C** before use.	Ambient
**Obtain**				
☐	**Visium Spatial Gene Expression Slide**<br>(with tissue sections)	2000233	Note the serial number on the slide label; will be required for downstream analysis.<br><br>For sample preparation, consult the Visium Spatial Protocols – Tissue Preparation Guide (CG000240).	-80°C
☐	**Slide Cassette**	3000406	See Tips & Best Practices.	Ambient
☐	**Isopropanol**	-	Manufacturer's recommendations.	Ambient
☐	**Hematoxylin, Mayer's (Lillie's Modification)**	-	Manufacturer's recommendations.	Ambient
☐	**Eosin Y Solution**	-	Manufacturer's recommendations.	Ambient
☐	**Bluing Buffer**	-	Manufacturer's recommendations.	Ambient
☐	**Milli-Q Water**	-	-	-
☐	**250 ml Vacuum Filter/ Storage Bottle System**<br>(0.2 µm Filter Nylon membrane)	-	-	-
☐	**Tris Base**	-	Manufacturer's recommendations.	-
☐	**Acetic Acid**	-	Manufacturer's recommendations.	-
**Prepare**				
☐	**Tris-Acetic Acid Buffer**<br>(0.45 M, pH 6.0)<br>pH meter will be required	Prepare **200 ml**, store at **room temperature**.<br>• Dissolve **11 g** Tris base in **100 ml** nuclease-free water.<br>• Adjust pH to 6.0 using 100% Acetic Acid.<br>• Bring volume to **200 ml** with nuclease-free water.<br>• Filter through **0.2 µm** nylon membrane filter system.		

*If using a Simport Scientific LockMailer Slide Mailer, dispense 10 ml/slide methanol.

1.1
Tissue Fixation

固定
！注意幻灯片标签上的序列号；下游分析将需要。

确保在50毫升离心管中分配的甲醇（40毫升/幻灯片）预冷至-20°C。

a．放置在热循环器上设置在37°C，平衡5分钟。 加热热循环器盖子不需要盖上的。

b．从-80°C中取出切片，置于密封容器中的干冰上
延迟将幻灯片转移到干冰可能导致凝结，这可能导致组织损伤和/或切片上组织切片的移动。

c.放置在PCR仪上，活性表面朝上，在37°C孵育1分钟。 不要关闭RCR仪盖子。 保持热循环器在37°C步骤1.2.
d.如有必要，从sl的背面擦拭多余的液体，不碰组织切片。

Note the serial number on the slide label; will be required for downstream analysis.

Ensure that the methanol (40 ml/slide) dispensed in a 50-ml centrifuge tube is chilled to **-20°C**.

a. Place a Thermocycler Adaptor on a thermal cycler set at **37°C** and equilibrate for **5 min**. Heating the thermal cycler lid is not required.

b. Remove slide from **-80°C** and place on dry ice in a sealed container.

Delay in transferring slides to dry ice may result in condensation, which may cause tissue damage and/or shifting of tissue sections on the slide.

c. Place on the Thermocyler Adaptor with the active surface facing up and incubate **1 min** at **37°C**. DO NOT close the thermal cycler lid. Maintain thermal cycler at **37°C** for step 1.2.

d. If necessary, wipe excess liquid from the back of the slide, without touching the tissue sections.

e. Completely immerse the slide in the pre-chilled methanol. Secure the tube cap to prevent methanol loss.

f. Incubate **30 min** at **-20°C**.

Place Thermocycler Adaptor

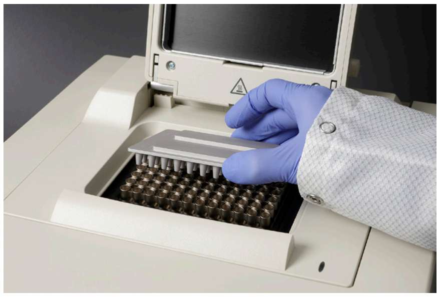

Incubate Slide for 1 min at 37°C

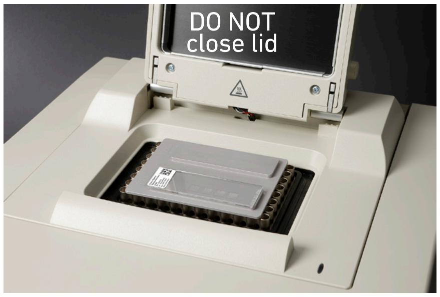

Incubate in Methanol for 30 min at –20°C

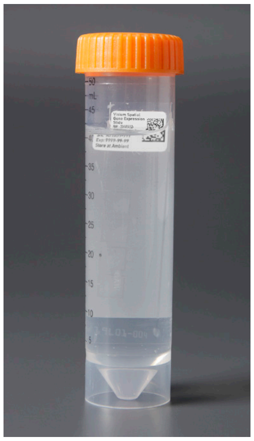

1.2 Tissue Staining

染色

a.分散以下体积的Q水
可以用于2张切片染色

b.配制伊红染液1ml

c. 从甲醇中取出载玻片，擦拭载玻片背面多余液体，不接触组织。放置在平整，洁净的作业面上。一些残留的液滴可能会残留。

d.加500μl异丙醇均匀覆盖玻片上所有组织（ 见贴士和最佳实践）。

TIPS

e.室温下孵育1分钟。用试剂孵育玻片时，要确保玻片不接触任何吸收表面，如实验室擦拭，可能吸收试剂。

f.丢弃试剂，通过排水和/或保持切片与底部边缘接触与实验室擦拭。

g.擦拭切片背面多余的液体，注意不接触组织切片。 放置在平整，洁净的作业面上。

h.空气干燥切片。 为了防止组织过度干燥，不要超过10分钟。

i. 加入1毫升苏木精染液，均匀覆盖所有组织。

j.室温孵育7分钟。

k.丢弃试剂，通过排水和/或保持切片与底部边缘接触与实验室擦拭。

a. Dispense the following volumes of Milli-Q water.
50 ml in one 50-ml centrifuge tube/slide
800 ml in Beaker 1
800 ml in Beaker 2
800 ml in Beaker 3
Dispensed volume in each beaker can be used for two slides.

b. Prepare Eosin Mix. DO NOT add pure eosin to tissue sections.

**Eosin Mix** *Prepare fresh. Vortex, centrifuge briefly.*	**Volume/slide (μl)**
Eosin Y Solution	100
Tris-Acetic Acid Buffer (0.45 M, pH 6.0)	900
**Total**	**1,000**

c. Remove slide from methanol and wipe excess liquid from the back of the slide, without touching the tissue sections. Place on a flat, clean work surface. Some residual droplets may remain.

d. Add **500 μl** isopropanol to uniformly cover all tissue sections on the slide. See Tips & Best Practices.

e. Incubate **1 min** at **room temperature**.
When incubating the slide with reagents, ensure that the slide is not in contact with any absorbent surface, like laboratory wipes, which may absorb the reagents.

f. Discard reagent by draining and/or holding the slide at an angle with the bottom edge in contact with a laboratory wipe.

g. Wipe excess liquid from the back of the slide, without touching the tissue sections. Place on a flat, clean work surface.

h. Air dry the slide. To prevent tissue section from over drying, DO NOT exceed **10 min**.

i. Add **1 ml** Hematoxylin to uniformly cover all tissue sections on the slide.

j. Incubate **7 min** at **room temperature**.

k. Discard reagent by draining and/or holding the slide at an angle with the bottom edge in contact with a laboratory wipe.

Incubate with Reagent

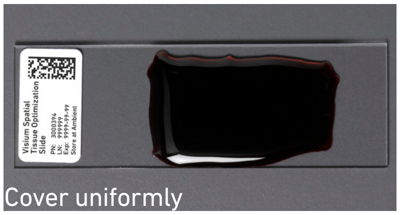

Discard Reagent

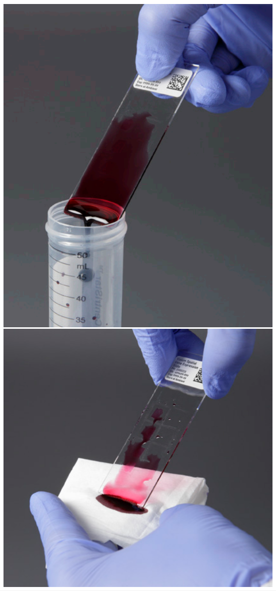

Slides in images are representative.

l.将幻灯片5x浸入离心管中的水中。

l. Immerse the slide 5x in the water in centrifuge tube.

m.将浸入烧杯1的水中15次。

m. Immerse the slide 15x in the water in Beaker 1.

n.将浸入烧杯2的水中15次。

n. Immerse the slide 15x in the water in Beaker 2.

Each immersion is ~1 sec

Correct Incorrect

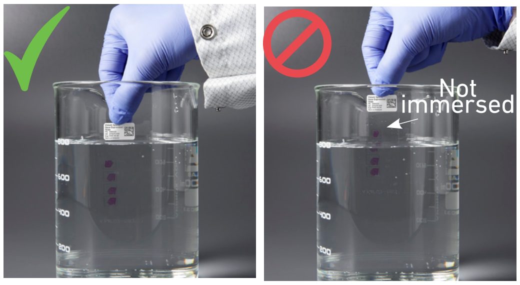

o.擦去切片背面多余的液体，不要接触组织。放置在平整、洁净的作业面上。一些液滴可能会残留。

o. Wipe excess liquid from the back of the slide without touching the tissue section. Place on a flat, clean work surface. Some droplets may remain.

p.加入1毫升Bluing缓冲液，均匀覆盖所有组织切片。

p. Add **1 ml** Bluing Buffer to uniformly cover all tissue sections.

q.室温孵育2分钟。

q. Incubate **2 min** at **room temperature**.

r.丢弃试剂，通过排水和/或保持切片与底部边缘接触与实验室擦拭。

r. Discard reagent by draining and/or holding the slide at an angle with the bottom edge in contact with a laboratory wipe.

s.将切片5x浸入烧杯2的水中。

s. Immerse the slide 5x in the water in Beaker 2.

t.擦去切片背面多余的液体，不要接触组织。

t. Wipe excess liquid from the back of the slide without touching the tissue section. Place on a flat, clean work surface. Some droplets may remain.

u.加入1毫升伊红溶液，覆盖组织。

u. Add **1 ml** Eosin Mix to uniformly cover all tissue sections.

v.室温孵育1分钟。

v. Incubate **1 min** at **room temperature**.

w.丢弃试剂，通过排水和/或保持切片与底部边缘接触与实验室擦拭。

w. Discard reagent by draining and/or holding the slide at an angle with the bottom edge in contact with a laboratory wipe.

x.将切片15x浸入烧杯3的水中

x. Immerse the slide 15x in the water in Beaker 3.

y. 擦去切片背面多余的液体.空气干燥，直到组织不透明。

y. Wipe the back of the slide with a laboratory wipe. Place on a flat, clean work surface. and air dry until tissue is opaque.

Incubate Slide

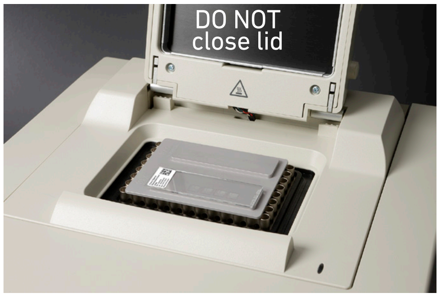

z.PCR仪37°C孵育5分钟。不关闭盖子。

z. Incubate slide on the Thermocycler Adaptor with the thermal cycler lid open for **5 min** at **37°C**.

开始成像。 可选的：在成像之前，可以在幻灯片上安装一个盖子。 见附录的封面应用和删除协议。

Proceed to imaging.
OPTIONAL: A coverslip may be mounted on the slide before imaging. See Appendix for Coverslip Application & Removal protocol.

1.3 Imaging

- Image all four Capture Areas individually at the desired magnification using brightfield imaging settings. See Imaging System Recommendations for more information.

Consult the Visium Spatial Gene Expression Imaging Guidelines Technical Note (CG000241) for complete information.

Step 2

Permeabilization & Reverse Transcription

2.1 Tissue Permeabilization

2.2 Reverse Transcription

2.0
Permeabilization & Reverse Transcription

在使用前，立即短暂离心并重悬在1.2mlHCl(0.1N)中，移液管混合，短暂离心，确认无沉淀。平衡到37°C。
储存未使用的再悬浮酶在-20°C。不要冻融超过3次。

CHECKLIST – GET STARTED!

Items	10x PN	Preparation & Handling	Storage
**Prepare & equilibrate to 37°C**			
☐ Permeabilization Enzyme	2000214	**Immediately** before use, centrifuge briefly and resuspend in 1.2 ml HCl (0.1N), pipette mix, centrifuge briefly, verify no precipitate. Equilibrate to 37°C.<br>! Store unused resuspended enzyme at −20°C. DO NOT freeze-thaw more than 3x.	−20°C
**Equilibrate to room temperature**			
☐ RT Reagent	2000086	Thaw, vortex, verify no precipitate, centrifuge briefly.	−20°C
☐ Template Switch Oligo	3000228	Centrifuge briefly, resuspend in 80 µl Low TE Buffer. Vortex 15 sec at maximum speed, centrifuge briefly, leave at room temperature for ≥ 30 min. After resuspension, store at −80°C.	−20°C
☐ Reducing Agent B	2000087	Thaw, vortex, verify no precipitate, centrifuge briefly.	−20°C
**Place on ice**			
☐ RT Enzyme D	2000216/ 2000227	Pipette mix, centrifuge briefly.	−20°C
**Obtain**			
☐ Nuclease-free Water	-	-	Ambient
☐ 20X SSC	-	-	Ambient
☐ Slide Seals	3000279	See Tip & Best Practices	Ambient
**Prepare**			

☐ **0.1X SSC** (can be prepared ahead of time)

**0.1X SSC** Store at room temperature	Stock	Final	50 ml
SSC	20X	0.1X	250 µl
Water	-	-	49.75 ml

2.1
Tissue Permeabilization

If a coverslip was mounted on the slide for imaging, remove the coverslip. See Appendix for Coverslip Application & Removal protocol.
Ensure that the Permeabilization Enzyme is resuspended and equilibrated to **37°C**.

a. Place a Thermocycler Adaptor in the thermal cycler. Prepare the thermal cycler with the following incubation protocol and start the program.

Lid Temperature	Reaction Volume	Run Time
37°C	-	*

Step	Temperature	Time
Pre-equilibrate	37°C	Hold
Permeabilization	37°C	*Determined by Tissue Optimization protocol.

b. Place the slide in the Slide Cassette.
See Tips & Best Practices for assembly instructions. Practice assembly with a blank slide.

c. Add **70 µl** Permeabilization Enzyme along the side of the wells to uniformly cover the tissue sections, without introducing bubbles.
Tap Slide Cassette gently to ensure uniform coverage.

d. Apply Slide Seal on the Slide Cassette and place the Slide Cassette on the Thermocycler Adaptor at **37°C**.

e. Close the thermal cycler lid and incubate for the pre-determined permeabilization time (tissue type specific).

Consult the Visium Spatial Gene Expression Reagent Kits – Tissue Optimization User Guide (CG000238) for the complete protocol for optimizing permeabilization time for any tissue of interest.

f. Remove the Slide Cassette from the Thermocycler Adaptor and place on a flat, clean work surface.

g. Using a pipette, remove Permeabilization Enzyme from the well corners.

h. Add **100 µl** 0.1X SSC to the wells.

Slide Cassette Assembly

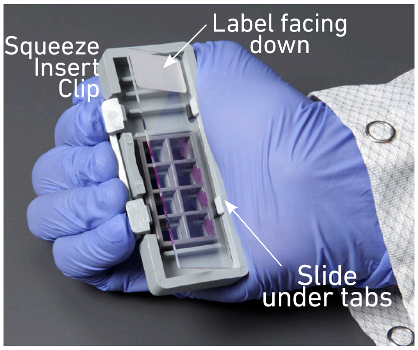

Add Reagent

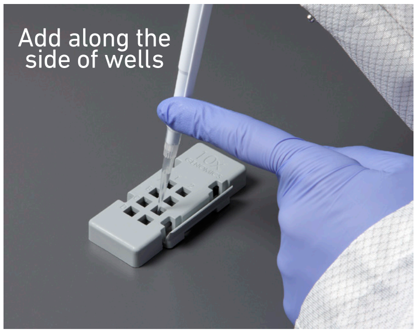

Apply Slide Seal

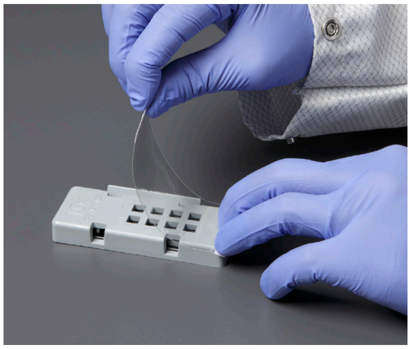

2.1组织透化

如果一个盖玻片安装在切片上进行成像，请移除盖玻片。 见附录的封面应用和删除协议。
确保透化酶重悬和预热37°C。

a. 准备PCR仪，并启动程序。

b. 把切片放在切片盒中。 见技巧和最佳做法的装配说明。用空白切片练习装配.

c.沿焊缝一侧加入70μl渗透酶，均匀覆盖组织切片，而不引入气泡。 轻轻敲击切片盒，以确保均匀覆盖。

d.在切片盒上贴上封条，并放置切片盒在37°C的PCR仪上。

e.关闭PCR仪盖子，并孵育预先确定的渗透时间（组织类型特定）。 查阅Visium空间基因表达，试剂盒-组织优化用户指南(C G000238)为任何感兴趣的组织优化渗透时间的完整协议。

f.从PCR仪上拆下切片盒，放置在一个平坦干净的工作表面。

g.使用移液管，从孔角处去除透化酶。

h.在孔中加入100μl0.1xSSC。

2.2
Reverse Transcription

2.2 反转录

a. 准备PCR仪，并启动程序

。

a. Place a Thermocycler Adaptor in the thermal cycler. Prepare a thermal cycler with the following incubation protocol and start the program.

Lid Temperature	Reaction Volume	Run Time
53°C	-	45 min
**Step**	**Temperature**	**Time**
Pre-equilibrate	53°C	Hold
Reverse Transcription	53°C	00:45:00
Hold	4°C	-

b.冰上配制RTmaster Mix混合物，吹打10x并离心。

b. Prepare RT Master Mix on ice. Pipette mix 10x and centrifuge briefly.

RT Master Mix *Add reagents in the order listed.*	PN	Volume/slide + 10% (µl)	Volume/2 slides + 10% (µl)
**Nuclease-free Water**	-	166.3	332.6
**RT Reagent**	2000086	82.7	165.4
**Template Switch Oligo**	3000228	22.9	45.8
**Reducing Agent B**	2000087	6.6	13.2
**RT Enzyme D**	2000085/ 2000227	51.5	103.0
**Total**	-	**330**	**660**

c.从孔中移除0.1xSSC。

d.在每孔中加入75µl RTmaster Mix混合物。

e.在切片盒上放置密封膜，并将其放置在预热的PCR仪上。 关闭盖子。

f.跳过预平衡步骤，启动反向转录。

c. Remove 0.1X SSC from the wells.

d. Add **75 µl** RT Master Mix to each well.

e. Apply Slide Seal on the Slide Cassette and place on the Thermocycler Adaptor on the pre-heated thermal cycler. Close the thermal cycler lid.

f. Skip Pre-equilibrate step to initiate Reverse Transcription.

Step 3

Second Strand Synthesis & Denaturation

3.1 Second Strand Synthesis 第二链合成

3.2 Denaturation 变性

3

3.0
Second Strand Synthesis

CHECKLIST – GET STARTED!

	Items	10x PN	Preparation & Handling	Storage
**Equilibrate to room temperature**				
☐	**Second Strand Reagent**	2000219	Thaw, vortex, centrifuge briefly.	–20°C
☐	**Second Strand Primer**	2000217	Thaw, vortex, centrifuge briefly.	–20°C
**Place on ice**				
☐	**Second Strand Enzyme**	2000218/ 2000183	Pipette mix, centrifuge briefly.	–20°C
**Obtain**				
☐	**Nuclease-free Water**	-		Ambient
☐	**0.08 M KOH** Dilute fresh from stock; prepare 500 µl/slide	-	Manufacturer's recommendations.	Ambient
☐	**Qiagen Buffer EB**	-	Manufacturer's recommendations.	Ambient
☐	**Tris-HCl** (1 M, pH 7.0)	-	Manufacturer's recommendations.	Ambient
☐	**Slide Seals**	3000279	See Tip & Best Practices.	Ambient

3.1
Second Strand Synthesis

a．在步骤2.2f的末尾，在热循环适配器平衡到4°C后，从PCR仪中拆下滑动盒，放置在平坦、干净的工作表面上。

b．将适配器放在PCR仪。准备PCR仪，并启动程序。

c.从孔中吸出RT主混合物。

d.每孔加入75μl0.08MKOH（从母液中稀释；确保准确稀释）。

e.室温下孵育5分钟。

f.从孔中吸出KOH。

g.每孔加入100μl EB。

h.在冰上准备第二链混合物。旋涡并轻轻离心。

i.从孔中吸出EB。

j．在每孔中加入75μl第二链混合物。

k.在滑动盒上涂上滑动密封，并放置在PCR仪中预热的热循环器适配器上。关闭热循PCR仪盖子。

l.跳过预平衡步骤，启动第二次Strand合成。

a. At the end of step 2.2f, after the Thermocycler Adaptor has equilibrated to **4°C**, remove the Slide Cassette from the thermal cycler and place on a flat, clean work surface.

b. Leave the Thermocycler Adaptor on the thermal cycler. Prepare the thermal cycler with the following incubation protocol and start the program.

Lid Temperature	Reaction Volume	Run Time
65°C	-	15 min
**Step**	**Temperature**	**Time**
Pre-equilibrate	65°C	Hold
Second Strand Synthesis	65°C	00:15:00
Hold	4°C	-

c. Remove RT Master Mix from the wells.

d. Add **75 µl** 0.08 M KOH (diluted from stock; ensure accurate dilution) to each well.

e. Incubate **5 min** at **room temperature**.

f. Using a pipette, remove KOH from the wells.

g. Add **100 µl** EB to each well.

h. Prepare Second Strand Mix on ice. Vortex and centrifuge briefly.

Second Strand Mix<br>*Add reagents in the order listed*	PN	Volume/slide + 10% (µl)	Volume/2 slides + 10% (µl)
**Second Strand Reagent**	2000219	305.8	611.6
**Second Strand Primer**	2000217	17.6	35.2
**Second Strand Enzyme**	2000218/<br>2000183	6.6	13.2
**Total**	-	**330**	**660**

i. Using a pipette, remove Buffer EB from the wells.

j. Add **75 µl** Second Strand Mix to each well.

k. Apply Slide Seal on the Slide Cassette and place on the Thermocycler Adaptor on the pre-heated thermal cycler. Close the thermal cycler lid.

l. Skip Pre-equilibrate step to initiate Second Strand Synthesis.

3.2 Denaturation

a．在孵育结束时，使用吸管，从孔中吸出试剂。

b．每孔加100μl缓冲EB。

c．从孔中吸出EB。

d．加入35μl 0.08M KOH。

e．室温下孵育10分钟。

f.在一个8连管中的4管中加入5μl Tris-HCl(1M，pH7.0），每个切片使用4管。

g.吸取35μl样本到对应的添加Tris-HCl管中。不要丢弃样品。 ~1-2μl体积变化是预期的。

h.涡旋，短暂离心，置于冰上。这时切片盒和切片可丢弃。

a. At the end of incubation, using a pipette, remove reagents from the wells.

b. Add **100 μl** Buffer EB to each well.

c. Using a pipette, remove Buffer EB from the wells.

d. Add **35 μl** 0.08 M KOH (diluted from stock) to each well.

e. Incubate **10 min** at **room temperature**.

f. Add **5 μl** Tris-HCl (1 M, pH 7.0) to 4 tubes in an 8-tube strip (4 tubes will be used for each slide).

g. Transfer **35 μl** sample from each well to a corresponding tube containing Tris-HCl in the 8-tube strip.
DO NOT discard sample. ~**1-2 μl** volume variation is expected.

h. Vortex, centrifuge briefly, and place on ice.

The Slide Cassette and slide may be discarded.

Step 4

cDNA Amplification & QC

4.1 Cycle Number Determination – qPCR

4.2 cDNA Amplification

4.3 cDNA Cleanup – SPRIselect

4.4 cDNA QC & Quantification

4.0
cDNA Amplification & QC

CHECKLIST – GET STARTED!

Item	10x PN	Preparation & Handling	Storage
**Equilibrate to room temperature**			
☐ ● **cDNA Primers**	2000089	Thaw, vortex, centrifuge briefly.	−20°C
☐ **Beckman Coulter SPRIselect Reagent**	-	Manufacturer's recommendations.	-
☐ **Agilent TapeStation Screen Tape and Reagents** If used for QC	-	Manufacturer's recommendations.	-
☐ **Agilent Bioanalyzer High Sensitivity kit** If used for QC	-	Manufacturer's recommendations.	-
☐ **DNA High Sensitivity Reagent Kit** If LabChip used for QC	-	Manufacturer's recommendations.	-
**Place on ice**			
☐ **KAPA SYBR FAST qPCR Master Mix** Minimize light exposure	-	Vortex, centrifuge briefly.	−20°C
☐ ○ **Amp Mix**	2000047/ 2000103	Vortex, centrifuge briefly.	−20°C
**Obtain**			
☐ **Qiagen Buffer EB**	-	-	Ambient
☐ **Nuclease-free Water**	-	-	-
☐ **qPCR Plate**	-	-	-
☐ **10x Magnetic Separator**	230003	See Tips & Best Practices.	Ambient
☐ **Prepare 80% Ethanol** Prepare 15 ml for 4 reactions (1 slide)	-	Prepare fresh.	Ambient
**Special Equipment**			
☐ **Real Time qPCR System**			

4.1
Cycle Number Determination – qPCR

a.在冰上准备qPCR混合物，见表。旋涡，并简短离心。

a. Prepare qPCR Mix on ice. Vortex and centrifuge briefly.

**qPCR Mix**<br>*Add reagents in the order listed.*<br>*Maintain on ice*	**PN**	**5X* + 10% (µl)**	**9X* + 10% (µl)**
		*Includes 1 negative control	
**Nuclease-free Water**	-	20.4	36.7
**KAPA SYBR FAST qPCR Master Mix**<br>Minimize light exposure	-	27.5	49.5
**cDNA Primers**	2000089	1.7	3.1
**Total**	-	**49.6**	**89.3**

b.每孔加入9µl qPCR混合物，包括阴性对照的孔。

b. Add **9 µl** qPCR Mix to each well in a qPCR plate (a well for negative control may be included).

c.将从步骤3.2h收获的1µl样品加入含有qPCR混合的qPCR板上。吸管吹打混匀，短暂离心。阴性对照孔中加入1µl无核酸酶水。

c. Transfer **1 µl** sample from **step 3.2h** to the qPCR plate well containing the qPCR Mix. Pipette mix, centrifuge briefly (if using a negative control, add **1 µl** nuclease-free water to the corresponding well).

d.准备一个带有以下协议的qPCR系统，放置板，并启动程序。

d. Prepare a qPCR system with the following protocol, place the plate, and start the program.

**Lid Temperature**	**Reaction Volume**	**Run Time**
-	10 µl	35 min
**Step**	**Temperature**	**Time**
**1**	98°C	00:03:00
**2**	98°C	00:00:05
**3**	63°C	00:00:30
**4**	Record amplification signal	
**5**	Go to step 2, for a total of 25 cycles	-

e.记录每个样本的Cq值。确定Cq值的阈值应沿放大图的指数阶段设置，~为峰值荧光值的25%。

e. Record the Cq Value for each sample.
The threshold for determining the Cq Value should be set along the exponential phase of the amplification plot, at ~25% of the peak fluorescence value.

Representative qPCR Amplification Plots

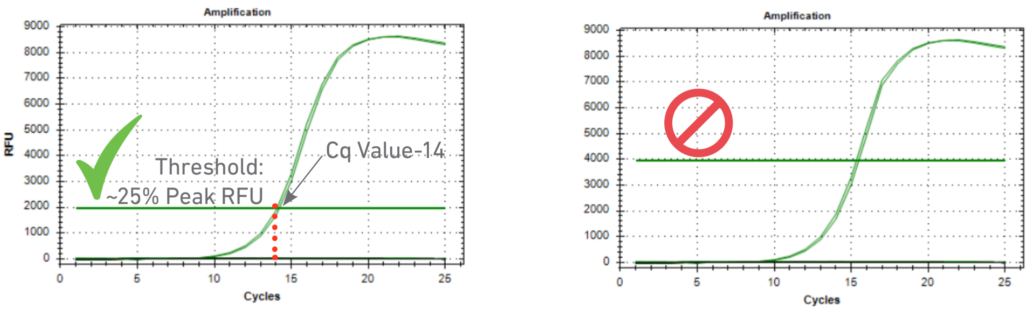

4.2
cDNA Amplification

a.冰上配制cDNA扩增混合物。混悬，并轻微离心。

b. 加入65µl混合物至步骤3.2h获得的cDNA剩余~35µl样品中。

c.pipette混合15次(吸管设置为90µl)。简单的离心。

d.在PCR仪中孵育，采用以下模式。

e.储存在4°C最多72小时或-20°C最多1周，或继续下一步。

a. Prepare cDNA Amplification Mix on ice. Vortex and centrifuge briefly.

	cDNA Amplification Mix<br>*Add reagents in the order listed*	PN	4X + 10% (µl)	8X + 10% (µl)
○	Amp Mix	2000047/ 2000103	220	440
●	cDNA Primers	2000089	66	132
	Total	-	**286**	**572**

b. Add **65 µl** cDNA Amplification Mix to remaining **~35 µl** sample from step 3.2h.

c. Pipette mix 15x (pipette set to 90 µl). Centrifuge briefly.

d. Incubate in a thermal cycler with the following protocol.

Lid Temperature	Reaction Volume	Run Time
105°C	100 µl	~45-60 min
**Step**	**Temperature**	**Time**
1	98°C	00:03:00
2	98°C	00:00:15
3	63°C	00:00:20
4	72°C	00:01:00
5	Go to Step 2, Use the Cq Value as the total # of cycles. See table below for total # of cycle examples	
6	72°C	00:01:00
7	4°C	Hold

Cycle number examples determined based on rounding the Cq Value.

Cq Value from qPCR	Total Cycles
12.2	12 cycles
13.5	14 cycles
13.8	14 cycles

STOP **e.** Store at **4°C** for up to **72 h** or at **–20°C** for up to **1 week**, or proceed to the next step.

4.3 cDNA Cleanup – SPRIselect

a.旋涡重悬SPRIselect试剂。 将60µl SPRIselect 试剂(0.6X)加入到每个样品(100µl)中，吸管混合15x(tips设置为150µl)。

b.室温下孵育5分钟。
c.放置磁铁上的E・高直到溶液变清。

d.移除上清液。
e.在球团中加入200ul80%乙醇，并等待30秒。

f.除去乙醇。

g.重复步骤e和f，共2次

h.离心机短暂离心，放置在磁铁・low。

STOP

i.除去任何剩余的乙醇。空气干燥2分钟。 不要超过2分钟，因为这将降低洗脱效率。

j.从磁铁上移开。加入40.5µl缓冲EB。吸管吹打混合15x(吸管设置为40µl)。

k.室温孵育2分钟。

l.将管条放置在磁铁上・低直到溶液清除。

m.将40µl样品转移到新管子带。 停止，

n. 4°C储存最多72小时或-20°C储存最多4周，或继续下一步。

a. Vortex to resuspend the SPRIselect reagent. Add **60 µl** SPRIselect reagent **(0.6X)** to each sample (100 µl) and pipette mix 15x (pipette set to 150 µl).

b. Incubate **5 min** at **room temperature**.

c. Place on the magnet•**High** until the solution clears.

d. Remove the supernatant.

e. Add **200 µl** 80% ethanol to the pellet. Wait **30 sec**.

f. Remove the ethanol.

g. **Repeat** steps e and f for a total of 2 washes.

h. Centrifuge briefly and place on the magnet•**Low**.

i. Remove any remaining ethanol. Air dry for **2 min**.
DO NOT exceed **2 min** as this will decrease elution efficiency.

j. Remove from the magnet. Add **40.5 µl** Buffer EB. Pipette mix 15x (pipette set to 40 µl).

k. Incubate **2 min** at **room temperature**.

l. Place the tube strip on the magnet•**Low** until the solution clears.

m. Transfer **40 µl** sample to a new tube strip.

n. Store at **4°C** for up to **72 h** or at **–20°C** for up to **4 weeks**, or proceed to the next step.

4.4 cDNA QC & Quantification

a. Run **1 µl** of sample on an Agilent Bioanalyzer High Sensitivity chip.
cDNA profile may vary depending on tissue type and quality.
Lower molecular weight product (35-150 bp) may be present. This is normal and does not affect sequencing or application performance.

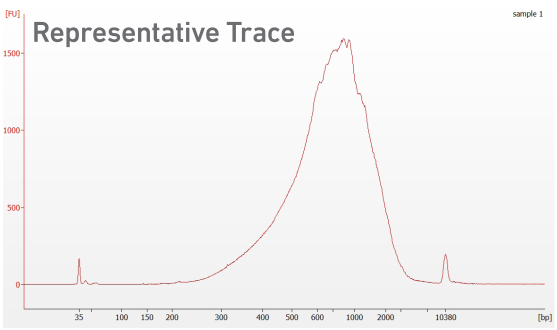

EXAMPLE CALCULATION

i. Select Region
Under the "Electropherogram" view choose the "Region Table". Manually select the region of ~200 – ~9000 bp.

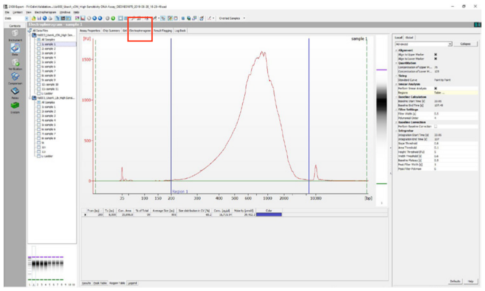

ii. Note Concentration [pg/µl]

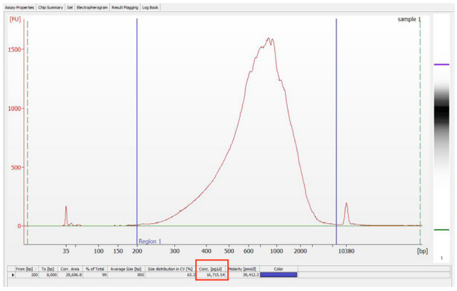

iii. Calculate
Multiply the cDNA concentration [pg/µl] reported via the Agilent 2100 Expert Software by the elution volume (40 µl) of the Post cDNA Amplification Reaction Clean Up sample and then divide by 1,000 to obtain the total cDNA yield in ng.

Example Calculation of cDNA Total Yield

Concentration: 16,715.54 pg/µl
Elution Volume: 40

Total cDNA Yield

$$= \frac{\text{Conc'n (pg/µl)} \times \text{Elution Volume (µl)}}{1000 \text{ (pg/ng)}}$$

$$= \frac{16{,}715.54 \text{ (pg/µl)} \times 40 \text{ (µl)}}{1000 \text{ (pg/ng)}} = 668.6. \text{ ng}$$

The carry forward cDNA volume is specified in step 5.1.
Refer to step 5.5e for appropriate number of Sample Index PCR cycles based on carry forward cDNA/input mass.

Alternate Quantification Methods:

- Agilent TapeStation
- LabChip

See Appendix for representative traces

Step 5

Spatial Gene Expression Library Construction

5.1 Fragmentation, End Repair & A-tailing

5.2 Post Fragmentation, End Repair & A-tailing Double Sided Size Selection – SPRIselect

5.3 Adaptor Ligation

5.4 Post Ligation Cleanup – SPRIselect

5.5 Sample Index PCR

5.6 Post Sample Index PCR Double Sided Size Selection – SPRIselect

5.7 Post Library Construction QC

5.0 Visium Spatial Gene Expression Library Construction

CHECKLIST – GET STARTED!

Item	10x PN	Preparation & Handling	Storage
**Equilibrate to room temperature**			
☐ ● **Fragmentation Buffer**	2000091	Vortex, verify no precipitate, centrifuge briefly.	−20°C
☐ ● **Adaptor Oligos**	2000094	Vortex, centrifuge briefly.	−20°C
☐ ● **Ligation Buffer**	2000092	Vortex, verify no precipitate, centrifuge briefly.	−20°C
☐ **Dual Index Plate TT Set A**	3000431	-	−20°C
☐ **Beckman Coulter SPRIselect Reagent**	-	Manufacturer's recommendations.	-
☐ **Agilent TapeStation Screen Tape and Reagents** If used for QC		Manufacturer's recommendations.	-
☐ **Agilent Bioanalyzer High Sensitivity kit** If used for QC	-	Manufacturer's recommendations.	-
☐ **DNA High Sensitivity Reagent Kit** If LabChip used for QC	-	Manufacturer's recommendations.	-
**Place on ice**			
☐ ● **Fragmentation Enzyme**	2000090/ 2000104	Pipette mix, centrifuge briefly before using.	−20°C
☐ ● **DNA Ligase**	220110/ 220131	Pipette mix, centrifuge briefly before using.	−20°C
☐ ○ **Amp Mix**	2000047/ 2000103	Vortex, centrifuge briefly.	−20°C
☐ **KAPA Library Quantification Kit for Illumina Platforms**	-	Manufacturer's recommendations.	-
**Obtain**			
☐ **Qiagen Buffer EB**	-		Ambient
☐ **10x Magnetic Separator**	230003	See Tips & Best Practices.	Ambient
☐ **Prepare 80% Ethanol** Prepare 20 ml for 8 reactions	-	Prepare fresh.	Ambient

Step Overview (Step 5.1d)

Correlation between input & library complexity

A Visium Spatial Gene Expression library is generated using a fixed proportion (10 µl, 25%) of the total cDNA (40 µl) obtained at step 4.3. The complexity of this library will be comparable to one generated using a higher proportion (>25%) of the cDNA. The remaining proportion (30 µl, 75%) of the cDNA may be stored at **4°C** for up to **72 h** or at **–20°C** for longer-term storage (up to **4 weeks**).

Correlation: cDNA input & Library Complexity

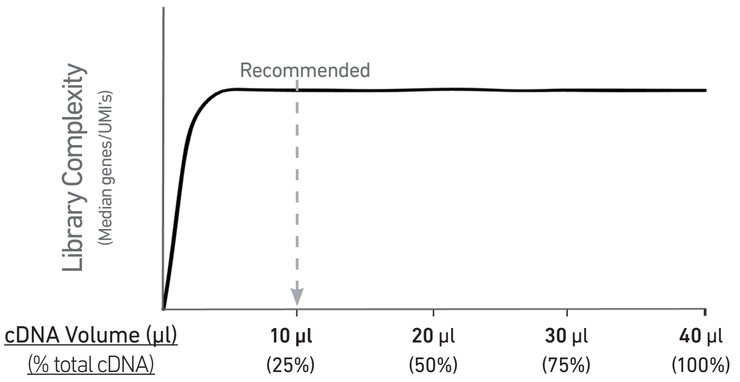

Note that irrespective of the total cDNA yield (ng), which may vary based on tissue type, coverage of Capture Area by tissue section, and tissue thickness, this protocol has been optimized for a broad range of input mass (ng), as shown in the example below. The total number of SI PCR cycles (step 5.5d) should be optimized based on carrying forward a fixed proportion (10 µl, 25%) of the total cDNA yield calculated during Post cDNA Amplification QC & Quantification (step 4.4).

Example: Library Construction Input Mass & SI PCR Cycles

Tissue Type	Tissue Covered Capture Area (%)	Total cDNA Amplification Cycles	Total cDNA Yield (ng)	cDNA Input into Fragmentation		SI PCR Cycle Number
				Volume (µl)	Mass (ng)	
High RNA Content	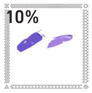	17	412	10	102	13
	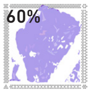	15	928	10	232	10
Low RNA Content	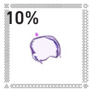	17	128	10	32	14
	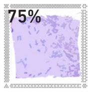	15	536	10	134	12

5.1 Fragmentation, End Repair & A-tailing

a. Prepare a thermal cycler with the following incubation protocol and start the program.

Lid Temperature	Reaction Volume	Run Time
65°C	50 µl	~35 min
**Step**	**Temperature**	**Time**
Pre-cool block<br>Pre-cool block prior to preparing the Fragmentation Mix	4°C	Hold
Fragmentation	32°C	00:05:00
End Repair & A-tailing	65°C	00:30:00
Hold	4°C	-

b. Prepare Fragmentation Mix on ice. Pipette mix and centrifuge briefly.

Fragmentation Mix<br>*Add reagents in the order listed*	PN	4X + 10% (µl)	8X + 10% (µl)
**Fragmentation Buffer**	2000091	22	44
**Fragmentation Enzyme**	2000090	44	88
**Total**	-	**66**	**132**

c. Transfer ONLY **10 µl** purified cDNA sample from cDNA Cleanup (step 4.3m) to a tube strip maintained on ice.
Note that only **10 µl** (25%) cDNA sample is sufficient for generating Visium Spatial Gene Expression library. The remaining **30 µl** (75%) cDNA sample can be stored at **4°C** for up to **72 h** or at **–20°C** for up **to 4 weeks** for generating additional libraries.

d. Add **25 µl** Buffer EB to each sample.

e. Add **15 µl** Fragmentation Mix to each sample.

f. Pipette mix 15x (pipette set to 35 µl) on ice. Centrifuge briefly.

g. Transfer into the pre-cooled thermal cycler **(4°C)**.

h. Skip Pre-cool block step to initiate Fragmentation.

5.2 Post Fragmentation, End Repair & A-tailing Double Sided Size Selection – SPRIselect

a. Vortex to resuspend SPRIselect reagent. Add **30 µl** SPRIselect **(0.6X)** reagent to each sample. Pipette mix 15x (pipette set to 75 µl).

b. Incubate **5 min** at **room temperature**.

c. Place on the magnet•**High** until the solution clears. DO NOT discard supernatant.

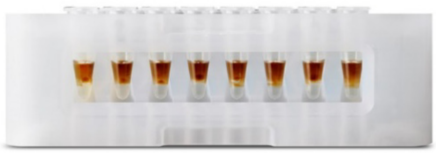

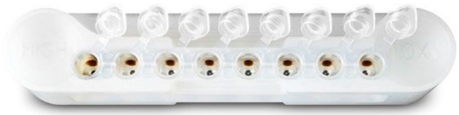

d. Transfer **75 µl** supernatant to a new tube strip.

e. Vortex to resuspend SPRIselect reagent. Add **10 µl** SPRIselect reagent **(0.8X)** to each sample. Pipette mix 15x (pipette set to 80 µl).

f. Incubate **5 min** at **room temperature**.

g. Place on the magnet•**High** until the solution clears.

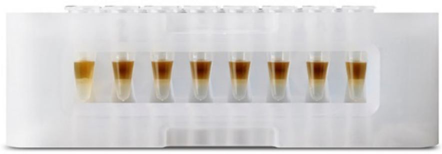

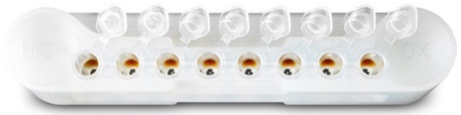

h. Remove **80 µl** supernatant. DO NOT discard any beads.

i. Add **125 µl** 80% ethanol to the pellet. Wait **30 sec.**

j. Remove the ethanol.

k. **Repeat** steps i and j for a total of 2 washes.

l. Centrifuge briefly. Place on the magnet•**Low** until the solution clears. Remove remaining ethanol. DO NOT over dry to ensure maximum elution efficiency.

m. Remove from the magnet. Add **50.5 µl** Buffer EB to each sample. Pipette mix 15x.

n. Incubate **2 min** at **room temperature**.

o. Place on the magnet•**High** until the solution clears.

p. Transfer **50 µl** sample to a new tube strip.

5.3 Adaptor Ligation

a. Prepare Adaptor Ligation Mix. Pipette mix and centrifuge briefly.

Adaptor Ligation Mix *Add reagents in the order listed*	PN	4X + 10% (µl)	8X + 10% (µl)
**Ligation Buffer**	2000092	88	176
**DNA Ligase**	220110/ 220131	44	88
**Adaptor Oligos**	2000094	88	176
**Total**	-	**220**	**440**

b. Add **50 µl** Adaptor Ligation Mix to **50 µl** sample. Pipette mix 15x (pipette set to 90 µl). Centrifuge briefly.

c. Incubate in a thermal cycler with the following protocol.

Lid Temperature	Reaction Volume	Run Time
30°C	100 µl	15 min
**Step**	**Temperature**	**Time**
1	20°C	00:15:00
2	4°C	Hold

5.4 Post Ligation Cleanup – SPRIselect

a. Vortex to resuspend SPRIselect Reagent. Add **80 µl** SPRIselect Reagent **(0.8X)** to each sample. Pipette mix 15x (pipette set to 150 µl).

b. Incubate **5 min** at **room temperature**.

c. Place on the magnet•**High** until the solution clears.

d. Remove the supernatant.

e. Add **200 µl** 80% ethanol to the pellet. Wait **30 sec**.

f. Remove the ethanol.

g. **Repeat** steps e and f for a total of 2 washes.

h. Centrifuge briefly. Place on the magnet•**Low**.

i. Remove any remaining ethanol. Air dry for **2 min**. DO NOT exceed **2 min** as this will decrease elution efficiency.

j. Remove from the magnet. Add **30.5 µl** Buffer EB. Pipette mix 15x.

k. Incubate **2 min** at **room temperature**.

l. Place on the magnet•**Low** until the solution clears.

m. Transfer **30 µl** sample to a new tube strip.

5.5 Sample Index PCR

a. Choose the appropriate sample index sets to ensure that no sample indices overlap in a multiplexed sequencing run. Record the 10x Sample Index name (PN-1000215 Dual Index Plate TT Set A well ID) used.

b. Add **50 µl** Amp Mix (PN-2000047 or 2000103) to **30 µl** sample.

c. Add **20 µl** of an individual Dual Index TT Set A to each well and record the well ID used. Pipette mix 5x (pipette set to 90 µl). Centrifuge briefly.

d. Incubate in a thermal cycler with the following protocol.

Lid Temperature	Reaction Volume	Run Time
105°C	100 µl	~25-40 min
**Step**	**Temperature**	**Time**
1	98°C	00:00:45
2	98°C	00:00:20
3	54°C	00:00:30
4	72°C	00:00:20
5	Go to step 2, see below for # of cycles	
6	72°C	00:01:00
7	4°C	Hold

The total cycles should be optimized based on 25% carry forward cDNA yield/input calculated during Post cDNA Amplification QC & Quantification (step 4.4)

Recommended cycle numbers

cDNA Input	Total Cycles
0.25-25 ng	14-16
25-150 ng	12-14
150-500 ng	10-12
500-1,000 ng	8-10
1,000-1,500 ng	6-8
>1500 ng	5

STOP

e. Store at **4°C** for up to **72 h** or proceed to the next step.

5.6 Post Sample Index PCR Double Sided Size Selection – SPRIselect

a. Vortex to resuspend the SPRIselect reagent. Add **60 µl** SPRIselect Reagent **(0.6X)** to each sample. Pipette mix 15x (pipette set to 150 µl).

b. Incubate **5 min** at **room temperature**.

c. Place on the magnet•**High** until the solution clears. DO NOT discard supernatant.

d. Transfer **150 µl** supernatant to a new tube strip.

e. Vortex to resuspend the SPRIselect reagent. Add **20 µl** SPRIselect Reagent **(0.8X)** to each sample. Pipette mix 15x (pipette set to 150 µl).

f. Incubate **5 min** at **room temperature**.

g. Place the magnet•**High** until the solution clears.

h. Remove **165 µl** supernatant. DO NOT discard any beads.

i. With the tube still in the magnet, add **200 µl** 80% ethanol to the pellet. Wait **30 sec**.

j. Remove the ethanol.

k. **Repeat** steps i and j for a total of 2 washes.

l. Centrifuge briefly. Place on the magnet•**Low**. Remove remaining ethanol.

m. Remove from the magnet. Add **35.5 µl** Buffer EB. Pipette mix 15x.

n. Incubate **2 min** at **room temperature**.

o. Place on the magnet•**Low** until the solution clears.

p. Transfer **35 µl** to a new tube strip.

STOP

q. Store at **4°C** for up to **72 h** or at **–20°C** for **long-term** storage.

5.7 Post Library Construction QC

Run **1 µl** of sample (1:10 dilution) on an Agilent Bioanalyzer High Sensitivity chip.

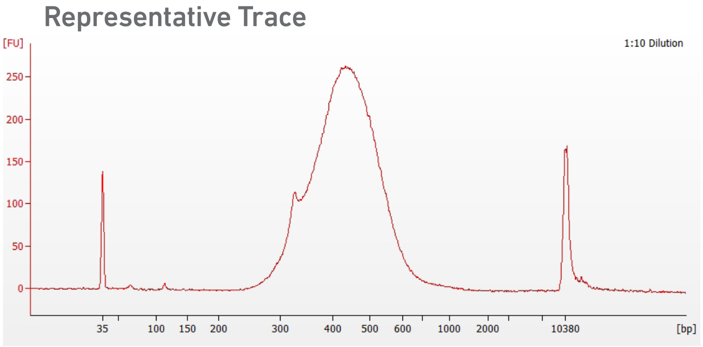

A smaller peak (~200-600 bp) may be present in some tissue types (e.g. mouse brain).

Determine the average fragment size from the Bioanalyzer trace. This will be used as the insert size for library quantification.

Alternate QC Method:

- Agilent TapeStation
- LabChip

See Appendix for representative traces

See Appendix for Post Library Construction Quantification

Sequencing

6

Sequencing Libraries

Visium Spatial Gene Expression libraries comprise standard Illumina paired-end constructs which begin with P5 and end with P7. 16 bp Spatial Barcodes are encoded at the start of TruSeq Read 1, while i7 and i5 sample index sequences are incorporated as the index read. TruSeq Read 1 and Read 2 are standard Illumina sequencing primer sites used in paired-end sequencing. TruSeq Read 1 is used to sequence 16 bp Spatial Barcodes and 12 bp UMI. Sequencing these libraries produce a standard Illumina BCL data output folder.

Visium Spatial Gene Expression Library

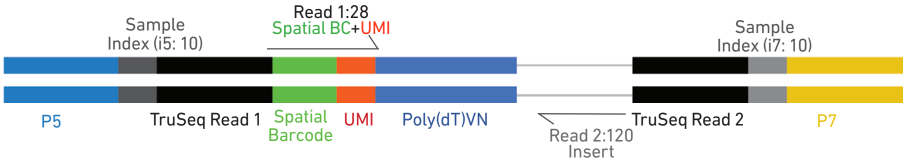

Sequencing Depth

**Sequencing Depth/spot**	Minimum 50,000 read pairs per tissue covered spot on Capture Area
**Sequencing Depth/sample**	See example calculation below

Example: Sequencing Depth for a Sample

- Estimate the approximate Capture Area (%) covered by the tissue section.
- **Calculate total sequencing depth=** (Coverage Area x total spots on the Capture Area) x 50,000 read pairs/spot
- **Example calculation for 60% coverage:** (0.60 x 5,000 total spots) x 50,000 read pairs/spot= 150 million total read pairs for that sample

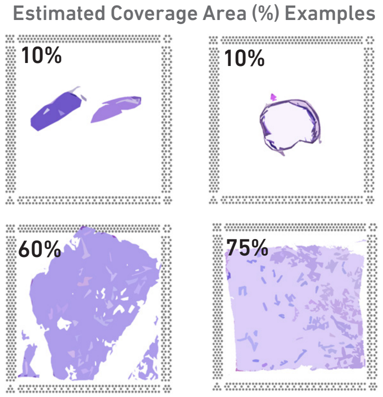

Sequencing Type & Run Parameters

Use the sequencing run type and parameters indicated.

Dual Index Library
Paired-end, dual indexed sequencing
Read 1: 28 cycles<br>i7 Index: 10 cycles<br>i5 Index: 10 cycles<br>Read 2: 120 cycles

Illumina Sequencer Compatibility

The compatibility of the listed sequencers has been verified by 10x Genomics. Some variation in assay performance is expected based on sequencer choice. For more information about performance variation, visit the 10x Genomics Support website.

- MiSeq
- NextSeq 500/550*
- HiSeq 2500 (Rapid Run)
- HiSeq 3000/4000
- NovaSeq
- iSeq

*Sequencing Visium Spatial Gene Expression libraries on the NextSeq 500/550 platform may yield reduced sequence quality and sensitivity relative to the MiSeq, HiSeq, and NovaSeq platforms. Refer to the 10x Genomics Support website for more information.

Sample Indices

Each well of the Dual Index Kit TT Set A (PN-1000215) contains a mix of one unique i7 and one unique i5 sample index. If multiple samples are pooled in a sequence lane, the sample index name (i.e. the Dual Index TT Set A plate well ID, SI-TT-) is needed in the sample sheet used for generating FASTQs with “spaceranger mkfastq”. Samples utilizing the same sample index should not be pooled together or run on the same flow cell lane, as this would not enable correct sample demultiplexing.

Library Loading

Once quantified and normalized, the Visium Spatial Gene Expression libraries should be denatured and diluted as recommended for Illumina sequencing platforms. Refer to Illumina documentation for denaturing and diluting libraries. Refer to the 10x Genomics Support website, for more information.

Instrument	Loading Concentration (pM)	PhiX (%)
MiSeq	11	1
NextSeq 500/550	1.8	1
HiSeq 2500 (RR)	11	1
HiSeq 4000	240	1
NovaSeq	150**/300	1
iSeq	150	1

** Use 150 pM loading concentration for Illumina XP workflow.

Library Pooling

The Visium Spatial Gene Expression libraries may be pooled for sequencing, taking into account the differences in tissue covered spot on a Capture Area and per-spot read depth requirements between each library. Samples utilizing the same sample index should not be pooled together, or run on the same flow cell lane, as this would not enable correct sample demultiplexing.

Troubleshooting

7

STEP	NOTES
Tissue Folding – Impact on UMI Count	**Folded tissue**<br>H&E stain 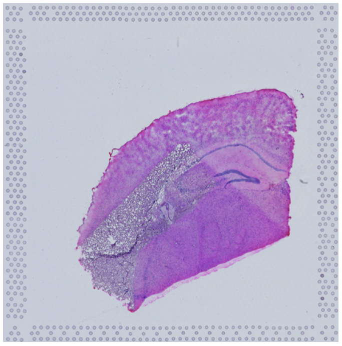 UMI count 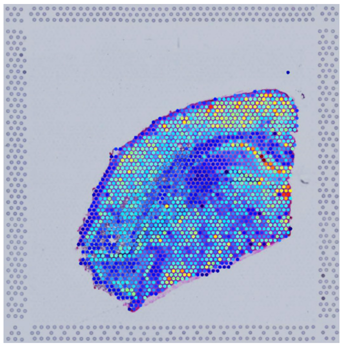<br>Folded tissue section can result in OCT induced tissue damage, impacting permeabilization, and diminishing assay sensitivity. However, the data derived from the rest of the tissue portions (not folded) can be analyzed.
Tissue Placement – Impact on UMI Count	**Fiducials are obstructed**<br>H&E stain 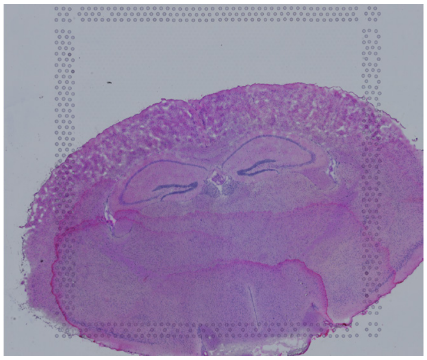 UMI Count 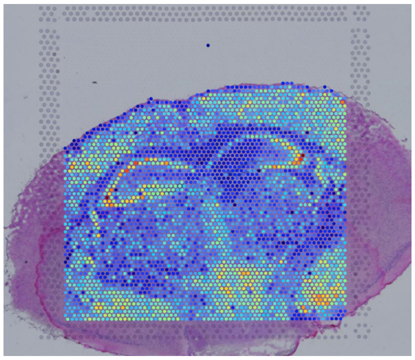<br>Fiducial obstruction may result in image analysis failure. Placement must be correct before the workflow begins. If necessary, software will prompt users to manually align tissue images during analysis.

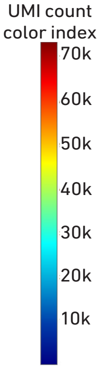

STEP	CORRECT	INCORRECT
1.2 Tissue Staining – Impact on UMI Count	Even H&E staining<br>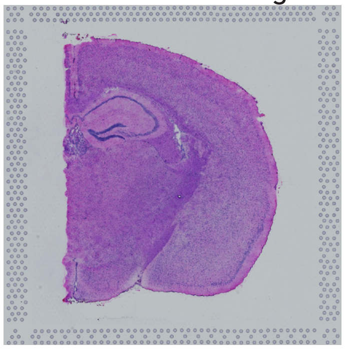<br>UMI Count<br>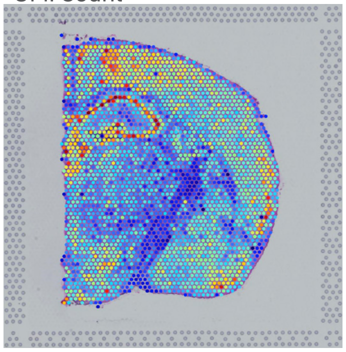	Uneven H&Estaining<br>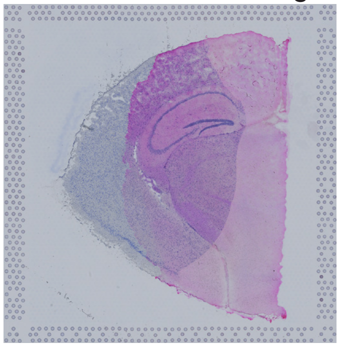<br>UMI Count<br>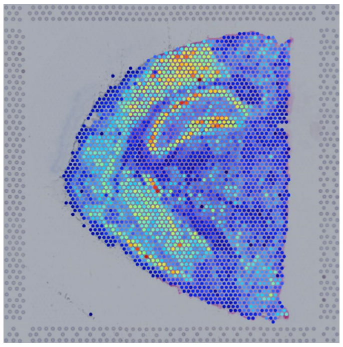

Ensure that staining reagents are applied to the tissue uniformly and adequate washes are performed. A gentle tap may help spread the reagent uniformly. Uneven staining may diminish sensitivity and spatial resolution. However, the data derived from the evenly stained tissue portions can be analyzed.

UMI count color index
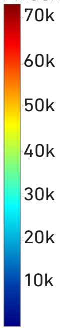

STEP	CORRECT	INCORRECT
2.1 Tissue Permeabilization – Reagent Coverage	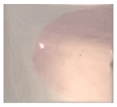 Slide Cassette Well<br>Tissue section covered completely<br>UMI Count<br>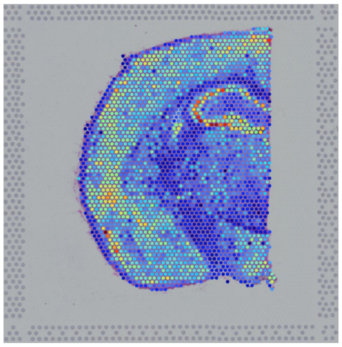	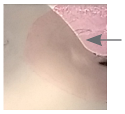 Slide Cassette Well<br>Tissue section not covered<br>UMI Count<br>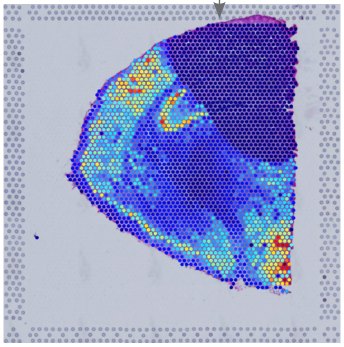
	Ensure that permeabilization reagents are applied to the tissue uniformly. Uneven permeabilization will diminish sensitivity and spatial resolution. However, the data derived from the optimally permeabilized tissue portions can be	
2.1 Tissue Permeabilization – Time	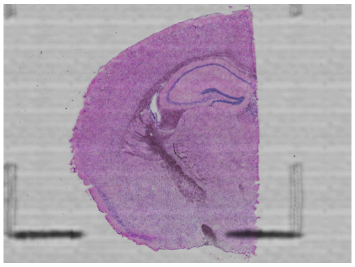<br>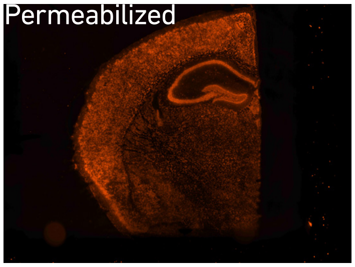	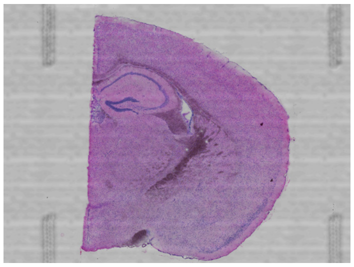<br>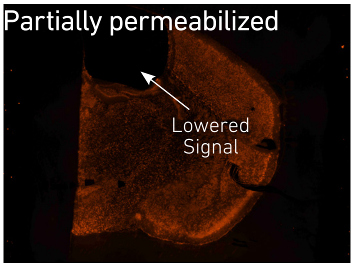
	Ensure that permeabilization times are optimized for each tissue type using the Visium Spatial Tissue Optimization protocol prior to beginning this workflow. Sub-optimal permeabilization will diminish sensitivity and spatial resolution.	
3.2 Denaturation – Partial	Cover the tissue section uniformly with 35 µl 0.08 M KOH to prevent partial denaturation.	
4.1 No Cq Value	Ensure that correct KOH dilution (0.08 M) is used at step 3.2d. Also, confirm that the qPCR mix includes KAPA SYBR FAST dye.	
4.4 Flat cDNA Trace (Cq value observed)	Flat cDNA trace, even though Cq value was observed at step 4.1.<br>Failure to properly neutralize KOH by addition of Tris-HCl (1 M, pH 7.0) at step 3.2f negatively impacts cDNA amplification efficiency (no impact on qPCR amplification, hence Cq value is observed).	

Appendix

Post Library Construction Quantification
Agilent TapeStation Traces
LabChip Traces
Coverslip Application & Removal
Oligonucleotide Sequences

8

Post Library Construction Quantification

a. Thaw KAPA Library Quantification Kit for Illumina Platforms.

b. Dilute **2 µl** sample with deionized water to appropriate dilutions that fall within the linear detection range of the KAPA Library Quantification Kit for Illumina Platforms. (For more accurate quantification, make the dilution(s) in duplicate).

c. Make enough Quantification Master Mix for the DNA dilutions per sample and the DNA Standards (plus 10% excess) using the guidance for 1 reaction volume below.

Quantification Master Mix	1X (µl)
SYBR Fast Master Mix + Primer	12
Water	4
**Total**	**16**

d. Dispense **16 µl** Quantification Master Mix for sample dilutions and DNA Standards into a 96 well PCR plate.

e. Add **4 µl** sample dilutions and **4 µl** DNA Standards to appropriate wells. Centrifuge briefly.

f. Incubate in a thermal cycler with the following protocol.

Step	Temperature	Run Time
1	95°C	00:03:00
2	95°C	00:00:05
3	67°C	00:00:30
4	Go to Step 2, 29X (Total 30 cycles)	

g. Follow the manufacturer's recommendations for qPCR-based quantification. For library quantification for sequencer clustering, determine the concentration based on insert size derived from the Bioanalyzer/TapeStation trace.

Agilent TapeStation Traces

Agilent TapeStation Traces

Agilent TapeStation High Sensitivity D5000 ScreenTape was used.
Protocol steps correspond to the Visium Spatial Gene Expression Reagent Kits User Guide (CG000239).

Protocol Step 4.4 – cDNA QC & Quantification

Run 2 µl sample mixed with 2 µl loading buffer. Ensure dilution factor is factored in when calculating cDNA yield/µl (divide by 2).

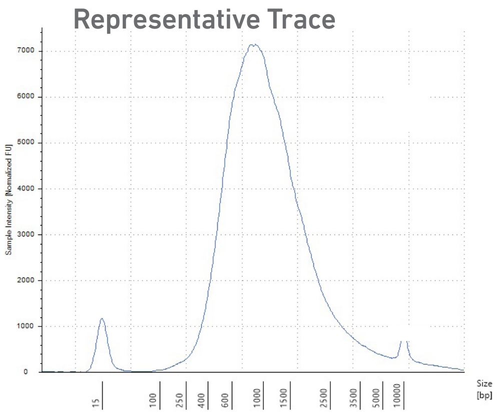

Protocol Step 5.7 – Post Library Construction QC

Run 2 µl diluted sample (1:10 dilution) mixed with 2 µl loading buffer.

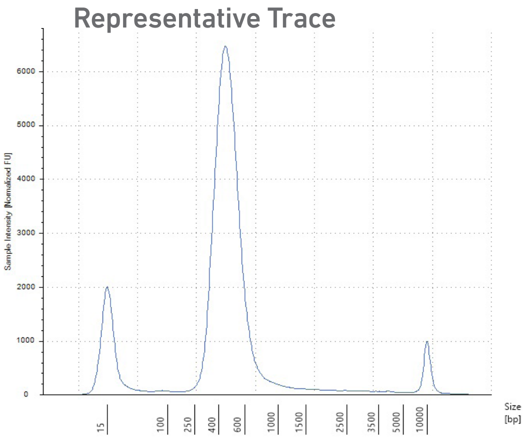

LabChip Traces

LabChip Traces

DNA High Sensitivity Reagent Kit was used.
Protocol steps correspond to the Visium Spatial Gene Expression Reagent Kits User Guide (CG000239).

Protocol Step 4.4 – cDNA QC & Quantification

Representative Trace

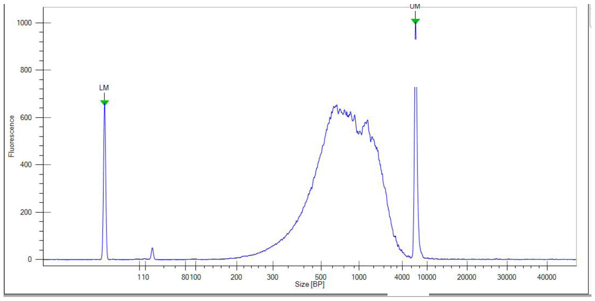

Run 10 µl undiluted sample.
cDNA yield calculation is same as Agilent Bioanalyzer traces.

Protocol Step 5.7 – Post Library Construction QC

Representative Trace

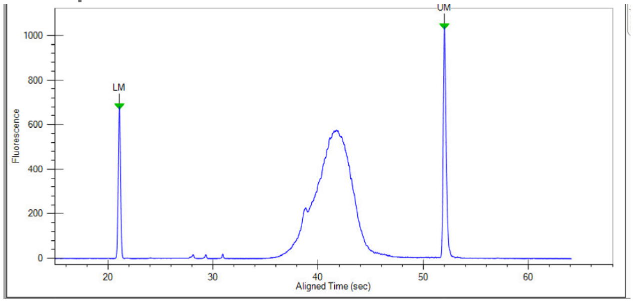

Run 10 µl diluted sample (1:10 dilution).

Coverslip Application & Removal

成像前可以在切片上加盖玻片，以提高光学质量。 虽然没有覆盖层的成像足以显示组织形态，但一些成像系统需要 关于盖玻片的使用。 如果使用盖玻片，请遵循此应用程序和移除协议，以确保组织切片和捕获区域不受损坏。

应用方法

应用程序在安装盖玻片之前，确保样品和带有组织的切片是干燥的。载玻片表面的水分可能溶解甘油，导致安装失败。如有必要，在37 ° C处孵育切片1分钟，放置在预平衡的热循环适配器上，盖子打开。

i．加入200μl85%甘油均匀覆盖切片上的组织。如果有必要，保持幻灯片的角度，以均匀覆盖。

二．将盖子用角度贴在的一端 他滑倒了。慢慢降低盖唇，用钳子轻轻压下，不引入气泡。

三．将多余的甘油放在实验室擦拭物上，然后将玻片的一个长边放掉来回倾斜切片。用幻灯片的第二长边重复。 重复这个过程，直到盖子固定为止。

四．保护好后，立即进行成像。不要让甘油附着的盖子干燥。不要使用软管或指甲油来固定盖子。

A coverslip may be mounted on the slides before imaging to enhance optical quality. Although imaging without a coverslip is sufficient to visualize the tissue morphology, some imaging systems require the use of coverslips.

If using a coverslip, follow this application and removal protocol to ensure that the tissue sections and the Capture Areas are not damaged.

Items
☐ **Large Coverslip** (Thermo Scientific 24 x 60 mm PN:22-050-233; Alternative, 24 x 50mm PN:22-050-232)
☐ **Milli-Q water (800 ml)**
☐ **80% Ethanol (50 ml)**
☐ **Laboratory Wipes**
☐ **Thermocycler Adaptor** (pre-equilibrated to **37°C** on a thermal cycler; may be used for drying)
☐ **Forceps**
☐ **85% Glycerol** (prepare **30 ml** – add **25.5 ml** 100% glycerol and **4.5 ml** Milli-Q water to a 50-ml centrifuge tube and vortex. Wait for the bubbles to dissipate or centrifuge at **300 rcf** for **1 min** before use)

Application

Prior to mounting the coverslip, ensure that the sample and the slide with the tissue sections are dry. Moisture on the surface of the slide may dissolve the glycerol, resulting in faulty mounting.

If necessary, incubate the slide for **1 min** at **37°C** by placing on the pre-equilibrated Thermocycler Adaptor placed on a thermal cycler with the lid open.

i. Add **200 µl** 85% glycerol to cover the tissue sections on the slide uniformly. If necessary, hold the slide at an angle for uniform coverage.

ii. Apply the coverslip at an angle on one end of the slide. Slowly lower the coverslip, pressing down gently with forceps, without introducing bubbles.

iii. Remove excess glycerol by placing one long edge of the slide on a laboratory wipe, and gently tilt the slide back and forth. Repeat with the second long edge of the slide. Repeat the process until the coverslip is secured.

iv. After the coverslip is secured, **immediately** proceed with imaging. DO NOT let the glycerol attached coverslip dry. DO NOT use Cytoseal or nail polish for securing the coverslip.

Cover uniformly with glycerol

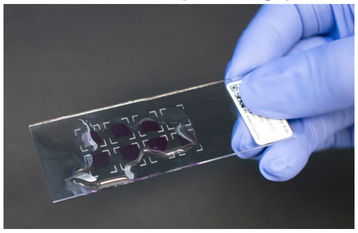

Apply coverslip

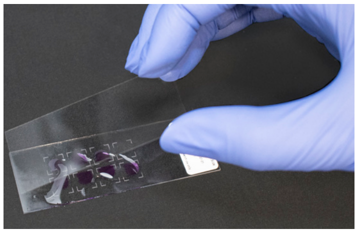

Press down

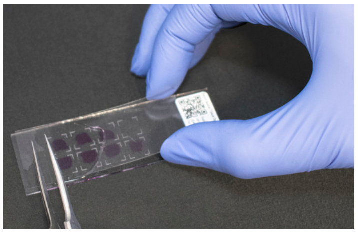

Remove excess glycerol

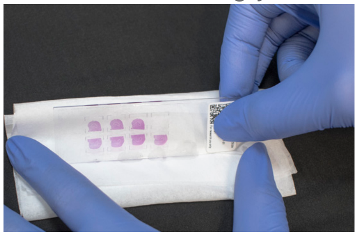

Coverslip Application & Removal

移除盖玻片

在成像完成后，立即移除盖玻片。

i. 在烧杯中装800毫升Milli-Q水，在50毫升离心管中加入50毫升80%乙醇。

二. 在~45 ° 浸泡切片在水中，被覆盖的表面完全淹没并朝下。

三. 把切片放在水里，直到盖玻片慢慢地从切片上分离出来。
不要向上移动玻片，用力向下或摇动，以防止损坏组织切片和捕获区域。

四. 一旦盖玻片分离，取出玻片，将玻片浸入~90 ° 角度的水中5x，以去除任何残留的甘油。

v.将玻片浸入80%乙醇中，在离心管中分配。

六. 室温空气干燥。如有需要，将载玻片置于37 ° C下孵育1分钟。在预平衡的热循环适配器上。 继续下一个工作流协议步骤。

Removal

Remove the coverslip immediately after imaging is complete.

i. Dispense **800 ml** Milli-Q water in a beaker and **50 ml** 80% ethanol in a 50-ml centrifuge tube.

ii. Immerse the slide at ~45° angle in the water with the coverslipped surface fully submerged and facing down.

iii. Hold the slide in water until the coverslip slowly separates away from the slide. DO NOT move the slide up and down or shake forcibly to prevent damaging the tissue sections and the Capture Areas.

iv. Once the coverslip is detached, remove slide and immerse the slide at ~90° angle in the water 5x to remove any residual glycerol.

v. Immerse the slide in 80% ethanol dispensed in the centrifuge tube.

vi. Air dry slide at room temperature. If necessary, incubate the slide for **1 min** at **37°C** by placing on a pre-equilibrated Thermocycler Adaptor.

Proceed to the next workflow protocol step.

Immerse in water

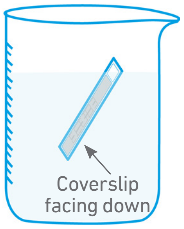

Hold in water

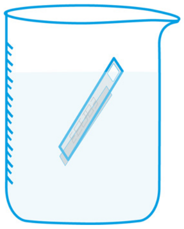

Coverslip detaches

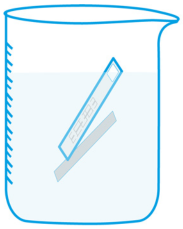

Oligonucleotide Sequences

Protocol steps correspond to the Visium Spatial Gene Expression Reagent Kits User Guide (CG000239)

Protocol Step 2.2 – Reverse Transcription

Slide Primers

5'-CTACACGACGCTCTTCCGATCT-NNNNNNNNNNNNNNNN-NNNNNNNNNNNN-TTTTTTTTTTTTTTTTTTTTTTTTTTTTTTVN-3'

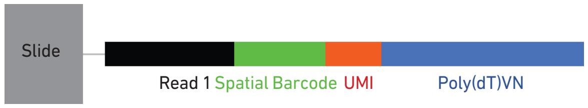

Template Switch Oligo PN -3000228

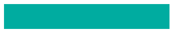

5'-AAGCAGTGGTATCAACGCAGAGTACATrGrGrG-3'

cDNA

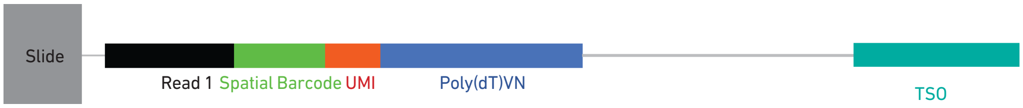

5'-CTACACGACGCTCTTCCGATCT-NNNNNNNNNNNNNNNN-NNNNNNNNNNNN-TTTTTTTTTTTTTTTTTTTTTTTTTTTTTTVN-cDNA_Insert-CCCATGTACTCTGCGTTGATACCACTGCTT-3'

Protocol Step 3.1 – Second Strand Synthesis

Second Strand Primer PN -2000217

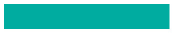

5'-AAGCAGTGGTATCAACGCAGAG-3'

Second Strand

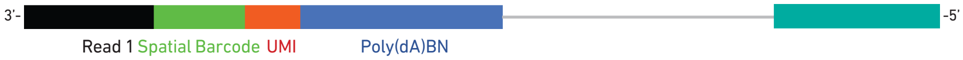

3'-GATGTGCTGCGAGAAGGCTAGA-NNNNNNNNNNNNNNNN-NNNNNNNNNNNN-AAAAAAAAAAAAAAAAAAAAAAAAAAAAAABN-cDNA_Insert-GGGTACATGAGACGCAACTATGGTGACGAA-5'

Protocol Step 4.2 – cDNA Amplification

cDNA Primers PN-2000089

Forward Primer:

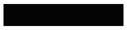

5'-CTACACGACGCTCTTCCGATCT-3'

Reverse Primer:

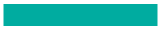

5'-AAGCAGTGGTATCAACGCAGAG-3'

Amplification Products

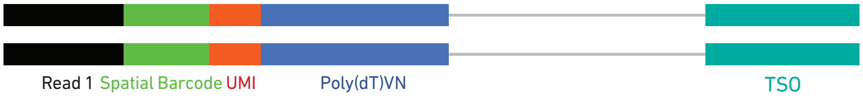

5'-CTACACGACGCTCTTCCGATCT-NNNNNNNNNNNNNNNN-NNNNNNNNNNNN-TTTTTTTTTTTTTTTTTTTTTTTTTTTTTTVN-cDNA_Insert-CCCATGTACTCTGCGTTGATACCACTGCTT-3'
3'-GATGTGCTGCGAGAAGGCTAGA-NNNNNNNNNNNNNNNN-NNNNNNNNNNNN-AAAAAAAAAAAAAAAAAAAAAAAAAAAAAABN-cDNA_Insert-GGGTACATGAGACGCAACTATGGTGACGAA-5'

Oligonucleotide Sequences

Protocol steps correspond to the Visium Spatial Gene Expression Reagent Kits User Guide (CG000239)

Protocol Step 5.3 – Adaptor Ligation

Adaptor Oligos
PN -2000094

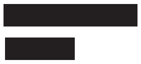

5'- GATCGGAAGAGCACACGTCTGAACTCCAGTCA-3'
3'-TCTAGCCTTCTCG-5'

Ligation
Product

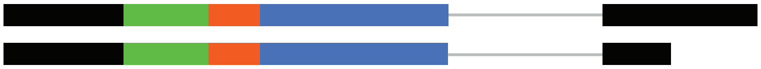

5'-CTACACGACGCTCTTCCGATCT-NNNNNNNNNNNNNNNN-NNNNNNNNNNNN-TTTTTTTTTTTTTTTTTTTTTTTTTTTTTTVN-cDNA_Insert-AGATCGGAAGAGC ACACGTCTGAACTCCAGTCAC-3'
3'-GATGTGCTGCGAGAAGGCTAGA-NNNNNNNNNNNNNNNN-NNNNNNNNNNNN-AAAAAAAAAAAAAAAAAAAAAAAAAAAAAABN-cDNA_Insert-TCTAGCCTTCTCG-5'

Protocol Step 5.5 – Sample Index PCR

Dual Indexing

Dual Index TT
Set A
PN-1000215

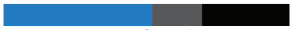

5'-AATGATACGGCGACCACCGAGATCT-NNNNNNNNNN-ACACTCTTTCCCTACACGACGCTC-3'

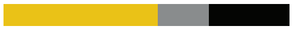

5'-CAAGCAGAAGACGGCATACGAGAT-NNNNNNNNNN- GTGACTGGAGTTCAGACGTGT-3'

Sample Index
PCR Product

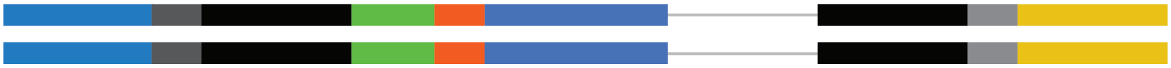

5'-AATGATACGGCGACCACCGAGATCT-NNNNNNNNNN-ACACTCTTTCCCTACACGACGCTCTTCCGATCT-NNNNNNNNNNNNNNNN-NNNNNNNNNNNN-TTTTTTTTTTTTTTTTTTTTTTTTTTTTTTVN-cDNA_Insert-AGATCGGAAGAGCACACGTCTGAACTCCAGTCAC-NNNNNNNNNN-ATCTCGTATGCCGTCTTCTGCTTG-3'
3'-TTACTATGCCGCTGGTGGCTCTAGA-NNNNNNNNNN-TGTGAGAAAGGGATGTGCTGCGAGAAGGCTAGA-NNNNNNNNNNNNNNNN-NNNNNNNNNNNN-AAAAAAAAAAAAAAAAAAAAAAAAAAAAAABN-cDNA_Insert-TCTAGCCTTCTCGTGTGCAGACTTGAGGTCAGTG-NNNNNNNNNN-TAGAGCATACGGCAGAAGACGAAC-5'
