# Supplementary material for: MIF promotes cell invasion by the LRP1-uPAR interaction in pancreatic cancer cells
Source: Front Oncol. 2023 Jan 10;12:1028070. doi: 10.3389/fonc.2022.1028070 (PMC9871987; doi:10.3389/fonc.2022.1028070)
Supplement: Supplementary file 3 [file DataSheet_3.pdf]

DEMONSTRATED PROTOCOL

Visium Spatial Protocols – Tissue Preparation Guide

Overview

The Visium Spatial Gene Expression Solution measures the total mRNA in tissue sections and requires a Visium Spatial slide with intact tissue sections as input. Proper tissue handling and preparation techniques preserve the morphological quality of the tissue sections and the integrity of mRNA transcripts. This is critical for downstream library preparation and generation of high quality sequencing data using the Visium Spatial Gene Expression protocols.

The Tissue Preparation Guide provides guidance on:

- Selecting appropriate Visium Spatial slides specific to the Visium Spatial protocol being used.
- Best practices for handling tissue samples and Visium Spatial slides before and after cryosectioning.
- Freezing and embedding tissue samples prior to cryosectioning.
- Cryosectioning of tissue samples and placement of sections on Visium Spatial slides.

Additional Guidance

This protocol was demonstrated using mouse brain tissues. However, the general principles for tissue preparation, cryosectioning, and storage are expected to be compatible with many tissue types (visit the 10x Genomics support website for a detailed list). Additional optimization may be required for the preparation of specialized tissues, such as tissue with high fat content.

The slides prepared using the Tissue Preparation Guide can be used with:

- Visium Spatial Gene Expression Reagent Kits – Tissue Optimization User Guide (CG000238)
- Visium Spatial Gene Expression Reagent Kits User Guide (CG000239)

Visium Slide Selection

Visium Spatial Tissue Optimization Slide

- Used with Visium Spatial Gene Expression Reagent Kits – Tissue Optimization User Guide (CG000238) to identify optimum permeabilization time for a specific tissue type.
- Includes 8 Capture Areas, each covered with oligonucleotides for mRNA capture.
- Each Capture Area is 8 x 8 mm and is surrounded by an etched frame.
- A readable label defines the active surface of the slide.

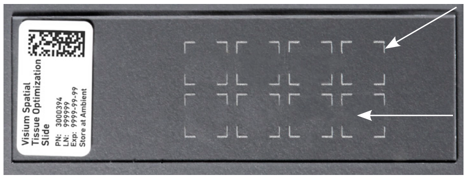

Visium Spatial Gene Expression Slide

- Used with Visium Spatial Gene Expression Reagent Kits User Guide (CG000239) to generate Visium Spatial Gene Expression libraries.
- Includes 4 Capture Areas, each with ~5,000 unique gene expression spots.
- Each Capture Area is 6.5 x 6.5 mm and is surrounded by a fiducial frame for a total area of 8 x 8 mm.
- A readable label with a serial number defines the active surface of the slide.

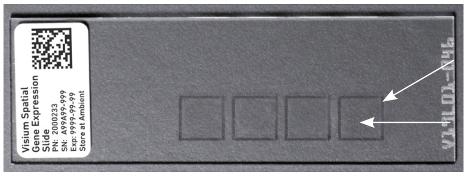

10x GENOMICS

Tips & Best Practices

Best Practices & Icons

- Best practices for handling any tissues include using sterile techniques, nuclease-free reagents and consumables.

Tips & Best Practices section includes additional guidance	Signifies critical step requiring accurate execution	Troubleshooting section includes additional guidance

Cryosectioning Temperature

- Cryosectioning temperatures impact tissue section integrity. A temperature setting of **–20°C** for cryostat chamber and **–10°C** for the specimen head is recommended.
- The temperature settings depend upon the local conditions, tissue types, and the cryostat used and should be optimized based on the quality of resulting tissue sections.
- During prolonged sectioning periods, allow the cryostat temperature to equilibrate by briefly closing the chamber.

Tissue Scoring

- A tissue section of ≤6.5 x 6.5 mm is compatible with Visium Spatial slides.
- OCT block with embedded tissue can be trimmed with a razor blade to fit the Capture Areas.
- Large tissue samples can be scored during sectioning to generate smaller samples to fit the Capture Areas.
- Scoring can be done by making a shallow incision (~1 mm deep) on the cutting surface of the tissue with a razor blade.
- The incision should be shallow. A deep incision may lead to tissue damage and disintegration.

Sectioning Speed

- Sectioning speed depends upon the desired thickness of the sections and the condition of the tissue. Harder and thicker sections require slow sectioning speed.
- Faster sectioning speed may lead to cracks or tears in the sections or damage to the tissue block or cryostat.

Section Thickness

- Recommended section thickness for most tissue types is 10 µm. Tissues with higher fat content (e.g., breast tissue) may require thicker sections.
- Visit the 10x Genomics support website for guidance on section thickness for compatible tissue types.

Handling Visium Slides

Handling Visium Spatial Slides Before Sectioning:

- Store unused slides in original packaging and keep sealed. DO NOT remove the desiccant.
- Equilibrate slides to cryostat temperature before proceeding with cryosectioning to prevent quick melting of the sample and the associated RNA degradation.

Handling Visium Spatial Slides Containing Tissue Sections:

- Maintain slides containing tissue sections in a low moisture environment.
- Keep slides cold and transport on dry ice.
- DO NOT leave slides at **room temperature**, especially with fresh sections as the resulting condensation will cause tissue disintegration.
- Store slides in a sealed container. If necessary, place slides in a secondary container, such as a resealable bag.
- Store slides individually (one slide per container) at **–80°C** to avoid multiple freeze-thaw cycles.

Section Placement on Slides

- Place the tissue section within the fiducial frame or the etched frames of the Capture Area on the pre-equilibrated Visium Spatial slides. Avoid covering the frames of the Capture Areas with the tissue.
- The section on the slides should be uniform without any cracks, tears, or folds.
- Only one section should be placed within each Capture Area.

Visium Spatial Gene Expression Slide

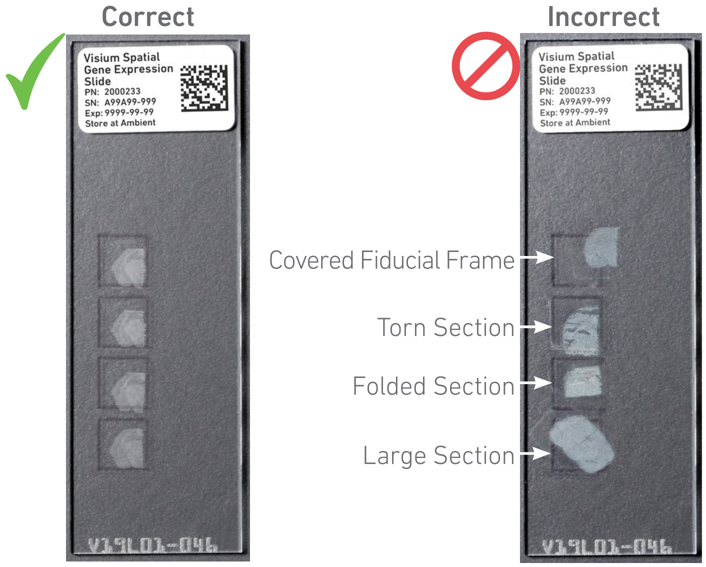

- For Visium Spatial Tissue Optimization Slide, place tissue sections on 7 of the 8 Capture Areas. Leave one Capture Area empty for positive RNA control.

Visium Spatial Tissue Optimization Slide

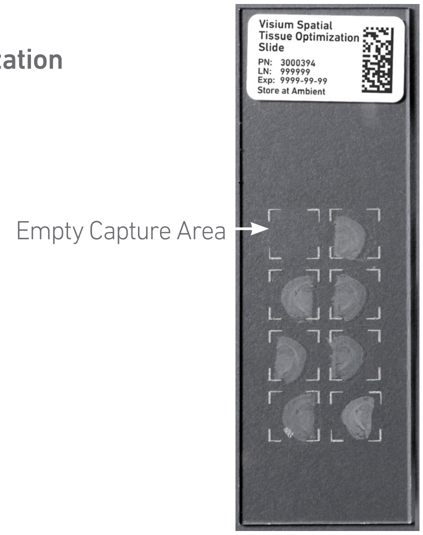

Practice Section Placement

- Create representative frames on a plain glass slide using the Visium Spatial Slide Layout (see Appendix).
- Practice correct section placement within the representative frames.

1. Tissue Freezing & Embedding

1.0 Overview

1.1 Reagents & Consumables

1.2 Tissue Freezing

1.3 Frozen Tissue Embedding

1.0 Overview

本章对组织冷冻和包埋提供指导.. 新鲜获得的组织样品必须被快速冷冻，以防止RNA降解，避免晶体形成，从而导致形态 组织的逻辑损伤。 一旦冷冻，组织样本被嵌入到冷冻和嵌入化合物，最佳切割温度(OCT)，以保持组织的结构和提供。 冷冻过程中的结构支持。 或者，对于有卷曲倾向的裂缝/缝隙或组织，在OCT中同时进行冷冻和嵌入（见附录） 详情）。

组织冷冻

用异戊烷和液氮浴对新鲜获得的组织进行冷冻。 由于温度不同，组织不应直接置于液氮中 NCE可能导致组织表面沸腾，导致空气袋和不均匀冻结。 这可能会裂开并在形态上损伤组织。

包埋

冰冻切片前的冷冻组织植入 冷冻组织样品嵌入OCT。与石蜡或树脂包埋技术不同，OCT不与影响抗原性的蛋白质和其他分子相互作用。

组织具有以下优点：

· 保存组织的结构，并在冷冻过程中提供结构支持。

*在切片过程中保持最佳温度我们通向平滑的路段。 新鲜组织冷冻组织嵌入组织冷冻异戊烷液氮OCT

· 兼容多种染色程序，因为它的水溶性。

This chapter provides guidance on tissue freezing and embedding. Freshly obtained tissue samples must be snap frozen to prevent RNA degradation and avoid crystal formation, which can lead to morphological damage to the tissue. Once frozen, tissue samples are embedded in a freezing and embedding compound, Optimal Cutting Temperature (OCT), to preserve the structure of the tissue and to provide structural support during cryosectioning.

Alternatively, perform simultaneous freezing and embedding in OCT for tissues with crevices/gaps or tissues that have a tendency to curl (see Appendix for details).

Tissue Freezing

A bath of isopentane and liquid nitrogen is used to freeze the freshly obtained tissue. Tissue should not be placed directly in liquid nitrogen as the temperature difference may cause boiling on the surface of the tissues, leading to air pockets and uneven freezing. This may crack and morphologically damage the tissue.

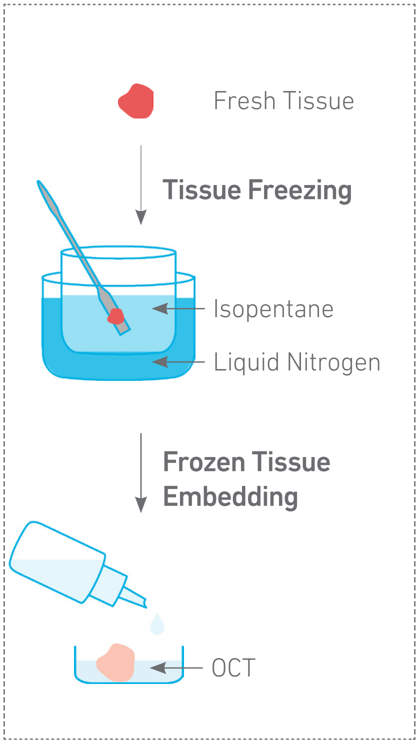

Frozen Tissue Embedding

Prior to cryosectioning, frozen tissue samples are embedded in OCT. Unlike paraffin or resin-based embedding techniques, OCT does not interact with proteins and other molecules that impact antigenicity.

OCT embedding of the tissue offers the following advantages:

- Preserves the structure of the tissue and provides structural support during cryosectioning.
- Maintains an optimal temperature during sectioning, thus leading to smooth sections.
- Compatible with multiple staining procedures due to its water solubility.

1.1 Reagents & Consumables

Tissue Freezing		
**Vendor**	**Item**	**Part Number**
Millipore Sigma	Isopentane (2-Methylbutane)	270342
VWR	Stainless Steel Beaker (250 ml)	89075-592
	Specimen Forceps, Straight, 203 mm (8")	82027-436
	Specimen Forceps, Straight, 152 mm (6")	82027-438
	Round/Tapered Spatula, Stainless Steel	82027-490
Wheaton	WHEATON 5 ml CryoELITE Tissue Vial	W985100

Frozen Tissue Embedding		
**Vendor**	**Item**	**Part Number**
VWR	TissueTek O.C.T. Compound	25608-930
	Disposable Based Molds (15 x 15 mm)<br>Dependent on the tissue size	60872-488

Additional Materials		
-	Dry Ice	-
-	Liquid Nitrogen	-
-	Razor Blades	-
-	Dewar for Liquid Nitrogen<br>Choose appropriate size based on the size of the steel beaker used	-

1.2 Tissue Freezing

在金属烧杯中填充三分之二的异戊烷（足以充分淹没组织），并放置在液氮杜瓦(与异戊烷相同的水平)，以允许足够的接触。 孵化15 min
使用卷起的实验室擦拭，从组织表面吸收多余的血液或溶液，以限制冰晶的形成。

Items	Preparation & Handling
**Prepare**	
☐ **Isopentane and liquid nitrogen bath**	Fill two-thirds of a metal beaker with isopentane (sufficient to fully submerge the tissue) and place in a liquid nitrogen dewar (same level as isopentane) to allow sufficient contact. Incubate **15 min**.
☐ **Tissue**	Using a rolled up laboratory wipe, absorb excess blood or solution from the surface of the tissue to limit ice crystal formation.

Isopentane and Liquid Nitrogen Bath

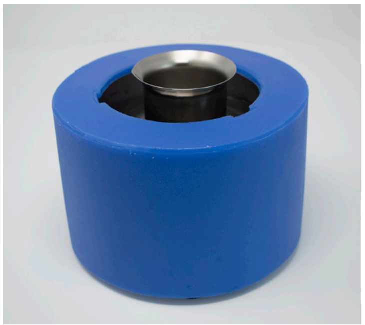

a. Using either forceps or a spatula, lower the tissue into the isopentane until fully submerged. Keep tissue submerged for ~**1 min** or until frozen. The freezing time may vary based upon the tissue type and size.

b. Once frozen, transfer the tissue to a pre-cooled WHEATON CryoELITE cryovial and place on dry ice.

c. Store frozen tissue at **–80°C** for **long-term** storage or **immediately** proceed to the next step (Frozen Tissue Embedding).

To prevent evaporation and dehydration of the tissue sample, snap-frozen tissue sample must be stored in a sealed container.

！！将冷冻组织储存在-80 ° C进行长期储存，或立即进行下一步（冷冻组织嵌入）。

！！为了防止组织样本的蒸发和脱水，快速冷冻组织，样品必须存放在密封的容器中。

1.3 Frozen Tissue Embedding

Items	Preparation & Handling
**Prepare**	
☐ **Powdered dry ice**	Use a mortar and pestle to prepare powdered dry ice.
☐ **Chilled OCT**	Place OCT in ice for ≥**30 min.**
☐ **Pre-cooled forceps**	Place forceps in dry ice for ≥**30 min.**
**Confirm**	
☐ **Cryomold**	The cryomold used for embedding should be of appropriate size to fit the tissue sample.

a. Label an appropriately sized cryomold to mark the orientation of the tissue.
Label the cryomold before adding OCT and tissue. The OCT will quickly turn white once frozen, making it hard to determine tissue orientation later.

b. Fill the cryomold with chilled OCT without introducing bubbles.

c. Remove frozen tissue from **–80°C** and transfer in dry ice.

d. Using pre-cooled forceps, place the frozen tissue into the OCT, covering any exposed surfaces with additional OCT.
Confirm there are no bubbles, especially near the tissue.

e. **Immediately** place the cryomold containing tissue and OCT on powdered dry ice.

f. Wait until the OCT is completely frozen.

g. Store the OCT embedded tissue block in a sealed container at **–80°C** for **long-term** storage or **immediately** proceed to Cryosectioning & Section Placement.

A WHEATON CryoELITE cryovial or a resealable bag can be used for storing the tissue block. Remove the tissue block from the cryomold and trim it using a razor blade to fit into the cryovial.

Failure to use a sealed container for storage may dehydrate and damage the tissue.

Frozen Tissue Embedding

Tissue in OCT

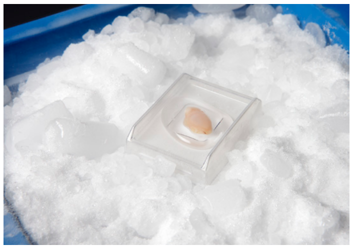

After Embedding

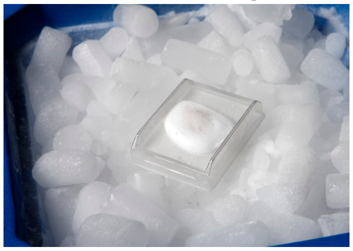

OCT Tissue Block Trimming

Before Trimming

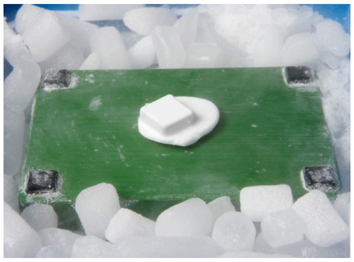

After Trimming

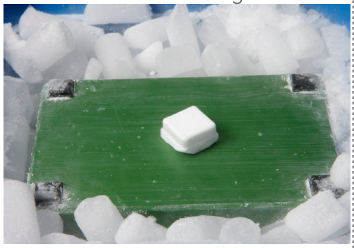

用研钵和杵制备粉末干冰.

a.标记一个适当大小的冷冻器来标记引入气泡。
 在加入OCT和组织之前给冷冻盒子贴上标签。 一旦冷冻，OCT将迅速变白，这使得很难确定组织的方向。
b.向冷冻盒里加入OCT。
c.从-80oC中取出冷冻组织并在干冰中转移。
d.用预冷镊子将冰冻组织放入 EOCT，用额外的OCT覆盖任何暴露的表面。 确认没有气泡，特别是在组织附近。
e.立即将含有组织和OCT的冷冻液放置在粉状干冰上。
f.等到OCT完全冻结。
g.将OCT嵌入的组织块存放在-80°C的密封容器中，以便长期储存，或立即进行冷冻和切片放置。
一个白色低温电子冷冻或一个可重新密封的袋子可以用来储存组织块。
从冷冻盒中取出组织块，用剃须刀刀片修剪，以适应冷冻模具。
使用密封容器储存，防止可能会脱水和损坏组织。
！ 停下！ 在OC中植入组织后修剪前组织阻滞修剪冷冻组织
 准备和处理准备粉状干冰使用砂浆和锤子准备粉状干冰。 冷冻OCT将OCT 放置在冰上≥30分钟。 预冷镊子置于干冰中≥30分钟。 确认Cryomold用于嵌入的Cryomold应该是合适的大小来适应组织样本。 组织冻结和嵌入方向的组织。

2. Cryosectioning & Section Placement

2.0 Overview

2.1 Reagents & Consumables

2.2 Cryosectioning

2.3 Section Placement

2.0 Overview

本章为OCT嵌入式组织的冷冻切割和组织切片在Visium空间幻灯片上的放置提供了指导。 根据Visium Spatial Pr选择合适的切片。 2. 切片和切片放置2.0概述2.1试剂和消耗品2.2冷冻2.3

切片
放置低温放置OCT嵌入组织块从TH中移除 在冷冻器中进行E-80oC存储和冷冻，以生成适当大小的Visium空间幻灯片，同时保持样品冷冻。

切片放置
组织切片放置在Visium空间切片上捕获区域。 每个捕获区域内只应放置一个组织。 对于视觉空间组织优化幻灯片，8个捕获区中有7个是我们组织的ED和一个是空的，用于阳性RNA对照。
每张切片只应测试一种组织类型。

This chapter provides guidance on cryosectioning of the OCT embedded tissue and placement of the tissue sections on the Visium Spatial slides.
Choose appropriate slide based on the Visium Spatial protocol being used.

Cryosectioning

OCT embedded tissue blocks are removed from the **–80°C** storage and cryosectioned in a cryostat to generate appropriately sized sections for Visium Spatial slides while keeping the samples frozen.

Section Placement

Tissue sections are placed within the frames of Capture Areas on Visium Spatial slides. Only one section should be placed within each Capture Area.

For Visium Spatial Tissue Optimization Slide, 7 of the 8 Capture Areas are used for tissue and one is left empty for a positive RNA control. Only one tissue type should be tested per slide.

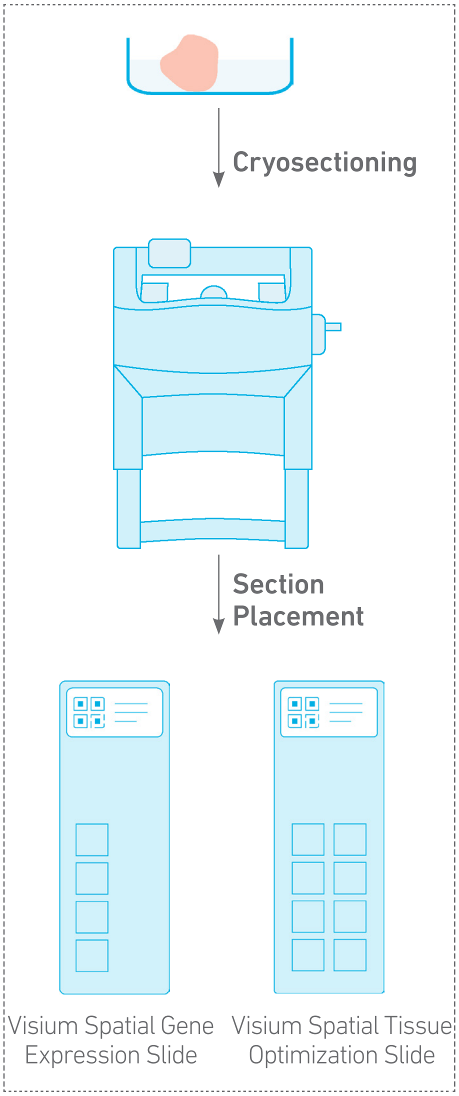

2.1 Reagents & Consumables

Vendor	Item	Part Number
VWR	TissueTek O.C.T. Compound	25608-930
	Sterile Centrifuge Tubes with Flat Caps, 50 ml	82018-050
10x Genomics	Visium Spatial Tissue Optimization Slide/ Visium Spatial Gene Expression Slide	3000394/ 2000233
Thermo Fisher Scientific	CryoStar NX70 Cryostat Vacutome, Low Profile Blade Carrier	957020
	Shandon ColorFrost Plus Slides (Optional)	6776214
	Flat cryostat brush, 10 mm	334160
	Brush, small beveled	334171
	Magnetic Brush, big	334172
Fisher Scientific	Thermo Scientific CryoStar NX70 Specimen Chuck	14-071-413
	Simport Scientific LockMailer Tamper Evident Slide Mailer (Alternatively, use a 50-ml centrifuge tube)	22-038-399
	MX35 Ultra Microtome Blade Low Profile	30-538-35350
	Glass Anti-Roll Plate	A78930200
**Additional Materials**		
-	Razor Blades	-
-	Dry Ice	-
-	Tissue Forceps	-

Cryostat Specifications

This protocol describes the use of a Cryostar NX70 Cryostat with specific capabilities. Alternatively, use a different cryostat with following features.

Function	Notes
Main Cryochamber	Separate and adjustable temperature control<br>Maintains stable temperatures from –8ºC to –35ºC
Specimen Head	Separate and adjustable temperature control<br>Maintains stable temperatures from –8ºC to –35ºC<br>X-axis and Y-axis adjustment
Blade Holder Base	Adjustable cutting angle<br>Adjustable blade position<br>Section thickness 10-50 μM
Cryobar	Rapid cooling

2.2 Cryosectioning

Items	Preparation & Handling
**Adjust**	
☐ **Cryostat temperature setting**	Recommended sectioning temperature is **–20°C** for cryostat chamber and **–10°C** for the specimen head.
**Equilibrate**	
☐ **Visium Spatial slides to the cryostat chamber temperature**	Slides should be cooled down to cryostat temperature for **≥30 min**. Warm slides will lead to quick melting of the sections and degradation of RNA.
☐ **OCT embedded tissue block to cryostat chamber temperature**	OCT embedded tissue block stored at **–80°C** must be equilibrated to cryostat chamber temperature for **30 min** before sectioning. If the tissue block is too cold, it will lead to section cracking. If the tissue block is too warm, it will lead to section compression or crumpling.

在标本阶段安装OCT嵌入式组织块：
a。 用OCT.填充试样阶段（夹头）。
b.将OCT嵌入的组织块放置在舞台上，切割表面远离切割表面 阶段
c.将标本头和组织块放置在低温室内的低温室上。
d.允许OCT和组织块冻结并附着在标本头上。

Mount OCT Embedded Tissue Block on the Specimen Stage:

a. Fill the specimen stage (chuck) with OCT.

b. Place the OCT embedded tissue block on the stage with the cutting surface facing away from the stage

c. Place the stage and the tissue block on the cryobar inside the cryostat chamber.

d. Allow the OCT and the tissue block to freeze and adhere to the specimen stage.

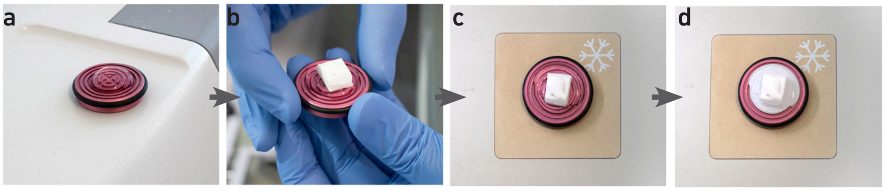

用冷冻机去除多余的OCT：
a.一旦冷冻，将带有组织块的安装到冷冻仪的标本头上，并开始切片以去除多余的OCT。
b.切片条件因不同而异 组织和冷冻剂。 按照制造商的建议进行冷冻。
c.继续切片直到组织可见。

Remove Excess OCT by Cryosectioning:

a. Once frozen, install the stage with the tissue block on to the specimen head of the cryostat and start sectioning to remove excess OCT.

b. Sectioning conditions vary across different tissues and cryostats. Follow manufacturer's recommendation for cryosectioning.

c. Continue sectioning until the tissue is visible.

Tissue Scoring:
Large tissue samples can be scored during sectioning to generate smaller samples to fit the Capture Areas. To score, make a shallow incision (~1 mm deep) on the cutting surface of the tissue with a pre-cooled razor blade. The incision should be shallow. A deep incision may lead to tissue damage and disintegration.

Example: To examine a specific region within one hemisphere of the mouse brain, scoring can be done by making a ~1 mm shallow incision at the midline of the brain.

Tissue Scoring

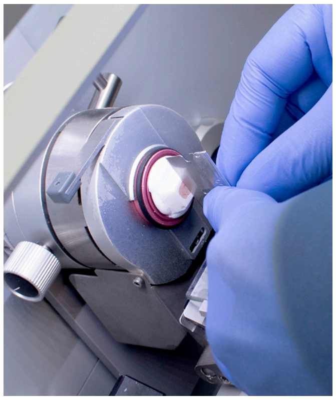

OPTIONAL

RNA Quality Assessment
RNA quality of the tissue block can be assessed at this stage by calculating RNA Integrity Number (RIN) of freshly collected tissue sections. See Appendix for details.

2.3 Section Placement

切片厚度
大多数组织类型的推荐切片厚度为10μm。 访问10x基因组学支持网站，以指导切片厚度的兼容组织类型。

防卷板已到位
辊板防止组织切片滚动。根据组织块大小优化防卷板位置。如有可能，在到达目标位置。

Items	Preparation & Handling
**Confirm**	
☐ **Section thickness setting**	Recommended section thickness is 10 μm for most tissue types. Visit the 10x Genomics support website for guidance on section thickness for compatible tissue types.
☐ **Anti-roll plate is in place**	Anti-roll plate prevents rolling of tissue sections. Optimize the position of anti-roll plate based on the tissue block size. If possible, adjust the position of anti-roll plate before reaching area of interest.

Position of Anti-roll Plate

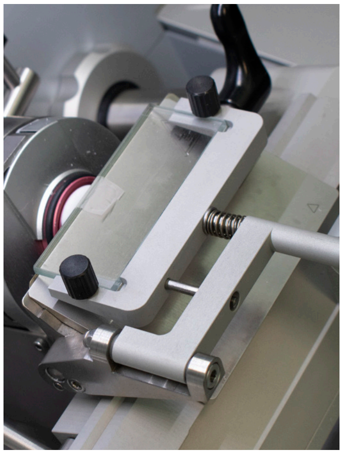

确认标本头温度，如果切片出现裂纹，试样头部太冷。 如果切片出现皱缩，标本头太温暖。 调节温度进行相应调节

切片放置练习
在普通玻璃切片上创建有代表性的框架，并在使用Visium空间幻灯片之前在框架内练习部分放置。
视觉空间幻灯片布局见附录。

Items	Preparation & Handling
☐ **Specimen head temperature**	Confirm the temperature of the specimen head. If the sections appear cracked, the specimen head is too cold. If the sections appear crumpled, the specimen head is too warm. Adjust temperature accordingly.
**Practice**	
☐ **Section placement on plain glass slides**	Create representative frames on a plain glass slide and practice section placement within the frames before working with the Visium Spatial slides. See Appendix for the Visium Spatial Slide Layout.

2.3 Section Placement

a.在普通玻璃幻灯片上练习切片放置。 视觉空间幻灯片布局见附录。

b. 一旦获得所需的组织切片，小心地将其压平，轻轻触摸周围OCT与低温器刷子。

c.将该部分放置在一个捕获区域内的预平衡Visium空间切片上，轻轻地触摸该部分与切片的活动表面。不要将切片放置在室温安抚。 应将切片放在冰冻切片机低温室以平衡切片稳定。 避免载玻片的活性表面与低温器接触，因为它会破坏寡核苷酸，降低了Visium空间幻灯片的捕获效率。

d.立即将一根手指放在幻灯片上的捕捉区背面几秒钟，以使该部分粘附完全。
确保整个组织完全粘附在切上，并且切片在整个切片放置过程中都在冷冻室内。
不要把切片从低温室移走。

e.立即将带有组织片的幻灯片放置在冷冻室上，以冻结切片。 继续切其他部分。
对于Visium空间组织优化幻灯片，将切片放置在8个捕获区域中的7个，将一个捕获区域空为阳性RNA对照。

f. 将切片发放给放置在干冰中的切片盒中。运送含有组织的切片

g.在-80oC存储幻灯片长达一周，或立即进行Visium空间组后续操作。 将切片单独（每个容器一张切片）存储在密封容器中。 如有必要，请放置幻灯片在二次容器中，如可重新密封的袋子。

将已贴敷上组织的切片保存在寒冷和低湿度的环境中。 不要将幻灯片暴露在室温下，因为由此产生的冷凝会导致组织崩解。

学习相见小贴士及最佳实践。

a. Practice section placement on plain glass slides. See Appendix for Visium Spatial slide layout.

b. Once desired tissue section is obtained, carefully flatten it out by gently touching the surrounding OCT with cryostat brushes.

c. Place the section within a Capture Area on the pre-equilibrated Visium Spatial slide by gently touching the section with the active surface of the slide.

DO NOT place sections on a room temperature slide. Slide should be equilibrated to cryostat chamber. Avoid contact between the active surface of the slide and the cryostat as it can damage the oligonucleotides and decrease the capture efficiency of the Visium Spatial slides.

d. **Immediately** place a finger on the backside of the Capture Area on the slide for a few seconds to allow the section to adhere to the slide.

Ensure that the entire tissue section is fully adhered to the slide and the slide is inside the cryostat chamber throughout section placement.
DO NOT remove the slide from the cryostat chamber at any point during sectioning and tissue placement.

e. **Immediately** place the slide with tissue section on the cryobar to freeze the section. Continue transferring sections on the remaining Capture Areas.

For Visium Spatial Tissue Optimization Slide, place sections on 7 of the 8 Capture Areas, leaving one Capture Area empty for positive RNA control.

f. Transfer the slide containing tissue sections to a slide mailer placed in dry ice.

g. Store slides at **–80°C** for up to a **week** or **immediately** proceed to Visium Spatial protocols.

Store slides individually (one slide per container) in a sealed container. If necessary, place the slides in a secondary container, such as a resealable bag.

Maintain slides containing sections in a cold and low moisture environment.
DO NOT expose slides to **room temperature** as the resulting condensation will cause tissue disintegration.

See Tips and Best Practices for handling slides.

Flatten the Section

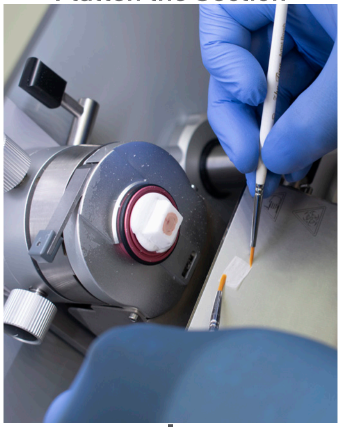

Transfer the Section

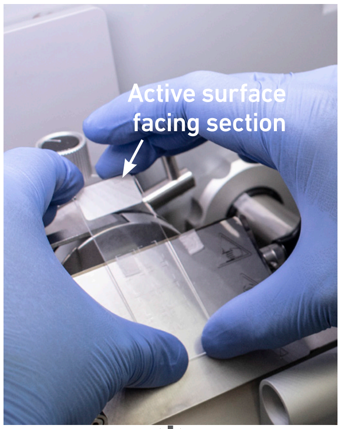

Adhere the Section

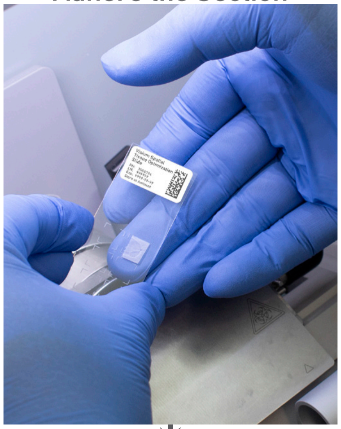

Immediately place the slide on the cryobar to allow section to freeze

Shipping of Slides:

If needed, slides containing tissue sections can be shipped on dry ice. See Appendix for detailed Shipping Guidelines.

Leftover Tissue Block Storage:

- Remove leftover tissue block attached to the specimen stage from the cryostat's specimen head and place onto cryobar.
- Cover the exposed tissue with OCT and allow to freeze.
- To separate the frozen tissue block from the stage, lift the tissue block and the stage from the cryobar and lightly warm the stage with hands or an aluminum block at room temperature

  Separation of the tissue block from the specimen stage is optional. The frozen tissue block can be stored attached to the specimen stage in a sealed container at **–80°C**.
- **Immediately** place the tissue block in dry ice. Ensure that the melted areas have refrozen.
- Store in a sealed container at **–80°C** for **long-term** storage.

Troubleshooting

Impact of Cryostat Specimen Head Temperatures on Tissue Tearing

-10°C

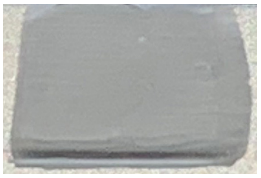

-14°C

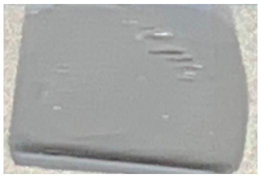

-20°C

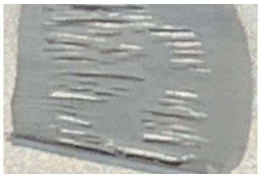

-30°C

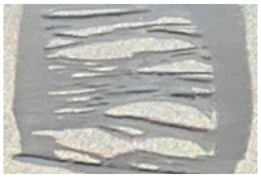

Normal Section

Torn sections. Adjust and confirm specimen head temperatures.

Impact of Condensation on Tissue Sections

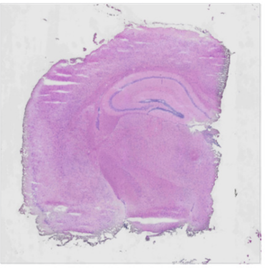

No Condensation. Intact tissue section.

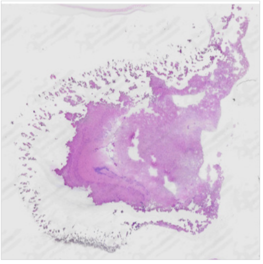

Tissue degraded due to condensation. DO NOT leave slides at room temperature, especially with fresh sections as the resulting condensation will cause tissue disintegration.

Incorrect Placement of Tissue Sections

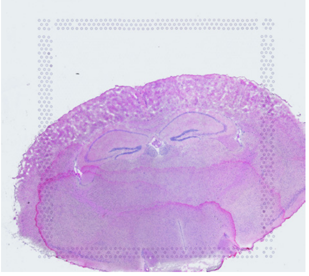

Fiducial frames covered

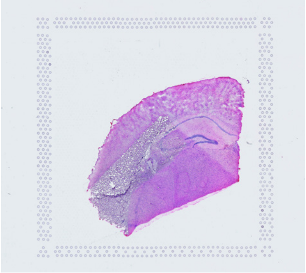

Folded tissue section

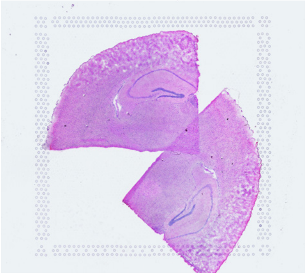

Overlapping sections

Practice correct section placement on blank glass slides before proceeding with Visium Spatial slides. See Appendix for Visium Spatial slide layout.

Images shown are tissue sections fixed with methanol and stained with hematoxylin and eosin (H&E).

Appendix

Simultaneous Freezing & Embedding

RNA Quality Assessment

Compatible Tissue Types

Visium Spatial Slide Layout

Shipping Guidelines

Simultaneous Freezing & Embedding

Items	Preparation & Handling
**Prepare**	
☐ **Isopentane and liquid nitrogen bath**	Fill two-thirds of a metal beaker with isopentane (sufficient to fully submerge the tissue) and place in a dewar of liquid nitrogen (same level as isopentane) to allow sufficient contact. Incubate for **15 min**.
☐ **Tissue**	Using a rolled up laboratory wipe, absorb excess blood or solution from the surface of the tissue to limit ice crystal formation.
**Confirm**	
☐ **Cryomold**	The cryomold used for embedding should be of appropriate size to fit the tissue sample.

a. In a petri dish, carefully coat fresh tissue sample with **room temperature** OCT. Confirm there are no bubbles on the surface of the tissue.

b. Using a spatula, place the OCT–coated tissue into an appropriately sized cryomold. Label the cryomold to mark the orientation of the tissue.

c. Fill the cryomold with additional OCT, ensuring that the tissue is completely covered. Confirm there are no bubbles, especially near the tissue.

d. Using forceps, lower the cryomold containing embedded tissue into the isopentane without fully submerging. Keep cryomold in contact with isopentane until the OCT has solidified and turned white.

If isopentane and liquid nitrogen are not available, powdered dry ice or a metal block chilled in dry ice can be used as an alternative.

e. Once frozen, place the cryomold on dry ice.

STOP

f. Store frozen embedded tissue in a sealed container at **–80°C** or liquid nitrogen for **long-term** storage or **immediately** proceed to Cryosectioning and Section Placement.

! Failure to use a sealed container for storage may dehydrate and damage the tissue.

RNA Quality Assessment

This section provides guidance on assessing the quality of the OCT embedded tissue blocks by calculating its RNA Integrity Number (RIN).

Vendor	Item	Part Number
Qiagen	RNeasy Mini Kit (50)	74104
	QIAshredder (50)	79654
Thermo Fisher Scientific	RNaseZap RNase Decontamination Solution	AM9780
	Nuclease-free Water	AM9937
Millipore Sigma	2-Mercaptoethanol	M6250-100ML
Eppendorf	DNA LoBind Tubes, 1.5 ml	022431021
Agilent	Agilent RNA 6000 Pico Kit	5067-1513
	Agilent RNA 6000 Nano Kit	5067-1511
**Additional Materials**		
-	Dry Ice	-
-	Tissue Forceps	-
-	Razor Blades	-

Pre-cool microcentrifuge tubes, cooling block, and forceps in cryostat chamber or at **–20°C** to prevent premature melting of the tissue sections.

a. Prepare 10 sections, each at 10 µm thickness.

b. Using the cooled forceps, pick up the sections and place inside a pre-cooled microcentrifuge tube.

c. Proceed to RNA isolation using Qiagen RNeasy Mini Kit or store at **–80°C** for **long-term** storage. Follow manufacturer's recommendation for RNA isolation. The section on Purification of Total RNA from Animal Tissues can be used for RNA isolation.

d. Store purified RNA at **–80°C** for **long-term** storage or **immediately** proceed to RIN calculation using either Agilent RNA 6000 Nano or Pico Kit. Follow manufacturer's instructions (Agilent) for RIN calculation.
The Visium Spatial protocol was optimized using samples with RIN ≥7.

在低温恒温器室或-20oC预冷微离心管、冷却块和镊子，以防止组织切片过早融化。
a、 准备10个截面，每个截面的厚度为10μm。
b、 用冷却的镊子取下切片，放入预冷却的微离心管内。
c、 使用Qiagen RNeasy微型试剂盒进行RNA分离或储存在-80度,用于长期储存。按照制造商的建议进行RNA分离。动物组织总RNA的纯化部分可用于RNA的分离。
d、 将纯化的RNA储存在-80o,用于长期储存或立即进行
使用Agilent RNA 6000 Nano或Pico试剂盒进行RIN计算。跟随
RIN计算的制造商说明（安捷伦）。
使用RIN≥的样本优化Visium空间协议

Visium Spatial Slide Layout

A layout of Capture Areas of Visium Spatial slides is shown below and can be used to create representative frames on plain glass slides to practice tissue section placement.

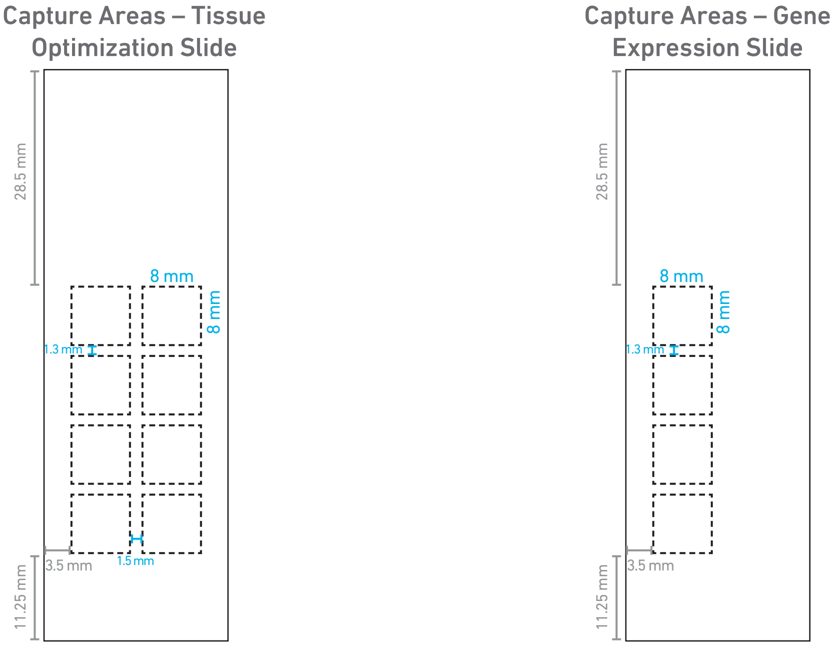

The slide dimensions represent a standard laboratory glass slide; printer settings may impact the image scaling.

For Visium Spatial Tissue Optimization Slide, each Capture Area is 8 x 8 mm and is surrounded by an etched frame. For Gene Expression Slide, each Capture Area is 6.5 x 6.5 mm and is surrounded by a fiducial frame for a total area of 8 x 8 mm.

Shipping Guidelines

- Place slides in a slide mailer. If multiple slides are being shipped, ensure that there is sufficient space in between the slides to avoid contact.
- Add folded paper towel to prevent excessive movement of the slides during shipping.
- Place the mailer in a sealed secondary container to limit exposure.
- Samples can be shipped overnight in dry ice, provided there is enough dry ice to account for transit and delivery times.
- Refer to the local institution or delivery service for detailed instructions on shipping samples in dry ice.

© 2019 10X Genomics, Inc. (10x Genomics). All rights reserved. Duplication and/or reproduction of all or any portion of this document without the express written consent of 10x Genomics, is strictly forbidden. Nothing contained herein shall constitute any warranty, express or implied, as to the performance of any products described herein. Any and all warranties applicable to any products are set forth in the applicable terms and conditions of sale accompanying the purchase of such product. 10x Genomics provides no warranty and hereby disclaims any and all warranties as to the use of any third-party products or protocols described herein. The use of products described herein is subject to certain restrictions as set forth in the applicable terms and conditions of sale accompanying the purchase of such product. A non-exhaustive list of 10x Genomics' marks, many of which are registered in the United States and other countries can be viewed at: www.10xgenomics.com/trademarks. 10x Genomics may refer to the products or services offered by other companies by their brand name or company name solely for clarity, and does not claim any rights in those third-party marks or names. 10x Genomics products may be covered by one or more of the patents as indicated at:www.10xgenomics.com/patents. The use of products described herein is subject to 10x Genomics Terms and Conditions of Sale, available at http://www:10xgenomics.com/legal-notices, or such other terms that have been agreed to in writing between 10x Genomics and user. All products and services described herein are intended FOR RESEARCH USE ONLY and NOT FOR USE IN DIAGNOSTIC PROCEDURES.

The use of 10x Genomics products in practicing the methods set forth herein has not been validated by 10x Genomics, and such non-validated use is NOT COVERED BY 10X GENOMICS STANDARD WARRANTY, AND 10X GENOMICS HEREBY DISCLAIMS ANY AND ALL WARRANTIES FOR SUCH USE. Nothing in this document should be construed as altering, waiving or amending in any manner 10x Genomics terms and conditions of sale for the Chromium Controller or the Chromium Single Cell Controller, consumables or software, including without limitation such terms and conditions relating to certain use restrictions, limited license, warranty and limitation of liability, and nothing in this document shall be deemed to be Documentation, as that term is set forth in such terms and conditions of sale. Nothing in this document shall be construed as any representation by 10x Genomics that it currently or will at any time in the future offer or in any way support any application set forth herein.

Contact:
support@10xgenomics.com
10x Genomics
6230 Stoneridge Mall Road
Pleasanton, CA 94588 USA

10x GENOMICS
