# Supplementary material for: MIF promotes cell invasion by the LRP1-uPAR interaction in pancreatic cancer cells
Source: Front Oncol. 2023 Jan 10;12:1028070. doi: 10.3389/fonc.2022.1028070 (PMC9871987; doi:10.3389/fonc.2022.1028070)
Supplement: Supplementary file 4 [file DataSheet_4.pdf]

PLANNER

Visium Spatial Gene Expression Protocol Planner

This document provides the time planner for the Visium Spatial Gene Expression protocol, along with recommendations for additional equipments, kits, and reagents.. The 10x Genomics Visium Spatial Reagent Kits are not listed in this document.

8 h

Steps	Timing	Stop & Store
**Tissue Staining & Imaging**		
Tissue Fixing	35 min	
Tissue Staining	30 min	
Tissue Imaging*	Variable	
**cDNA Synthesis**		
Tissue Permeabilization	15 min	
Reverse Transcription	65 min	
**Second Strand Synthesis & Denaturation**		
Second Strand Synthesis	25 min	
cDNA Denaturation	15 min	
**cDNA Amplification & QC**		
Cycle Number Determination – qPCR	45 min	
cDNA Amplification	45-60 min	STOP 4°C ≤72 h or –20°C ≤1 week
cDNA Cleanup – SPRIselect	20 min	STOP 4°C ≤72 h –20°C ≤4 weeks
cDNA QC & Quantification*	50 min	
**Visium Spatial Gene Expression Library Construction**		
Fragmentation, End Repair & A-tailing	50 min	
Post Fragmentation, End Repair & A-tailing Double Sided Size Selection – SPRIselect	30 min	
Adaptor Ligation	25 min	
Post Ligation Cleanup- SPRIselect	20 min	
Sample Index PCR	40 min	STOP 4°C ≤72 h
Post Sample Index PCR Double Sided Size Selection- SPRIselect	30 min	STOP 4°C ≤72 h or -20°C long term
Post Library Construction QC*	50 min	

*~8 h workflow excludes imaging time (varies based on imaging system) & QC steps

Cryostat Specifications

The Cryostar NX70 Cryostat with listed features was used by 10x Genomics. Any equivalent system with the listed features may be used.

Component	Features
Main Cryochamber	• Separate and adjustable temperature control<br>• Maintains stable temperatures from -8°C to -35°C
Specimen Head	• Separate and adjustable temperature control<br>• Maintains stable temperatures from -8°C to -35°C<br>• X-axis and Y-axis adjustment
Blade Holder Base	• Adjustable cutting angle<br>• Adjustable blade position<br>• Cuts 10-50 μM thick sections
Cryobar	• Rapid cooling

Additional Items for Cryosectioning

Vendor	Item	Part Number (US)
VWR	Optimal Cutting Temperature TissueTek	25608-930
10x Genomics	Visium Spatial Tissue Optimization Slide /<br>Visium Spatial Gene Expression Slide	3000394/<br>2000233
Thermo Fisher Scientific	CryoStar NX70 Cryostat, Vacutome, Low Profile Blade Carrier	957020
	30 mM Specimen Chuck	715870-CN
	Shandon ColorFrost Plus Slides (Optional)	6776214
	Simport Scientific LockMailer Tamper Evident Slide Mailer (case of 500)	22-038-399
	Flat cryostat brush, 12 mm	334160
	MX35 Ultra Microtome Blade (Low-Profile)	3053835
	Anti-Roll Plate (Glass)	1407086
	Brush, small beveled	334171
	Magnetic Brush, big	334172

Imaging System Recommendations

The microscope systems listed below were used by 10x Genomics. Any equivalent system with the listed features may be used for imaging.

Imaging Systems & Specifications	
**Microscopes** (Any equivalent system with the listed features may be used for imaging)	
Nikon	Nikon Eclipse Ti2 with brightfield and fluorescence capacity (TRITC)
Molecular Devices	ImageXpress Nano Automated Slide Imaging System
**Microscope Features**	
Objectives	• 4X (Plan APO λ; NA 0.20)<br>• 10X (Plan APO λ; NA 0.45)<br>• 20X (Plan APO λ; NA 0.75)
Scanning Stage	Microscope tile scanning functionality is required for imaging tissue sections placed on a Capture Area of a Visium Spatial slide.
Brightfield Features	• Color camera (3 x 8 bit, 2,424 x 2,424 pixel resolution)<br>• White balancing functionality<br>• Minimum Capture Resolution 2.18 µm/pixel<br>• Exposure times 2-10 milli sec
Fluorescence Features*	• Light source (or equivalent) with a wavelength range of 380-680 nm<br>• Monochrome camera (14 bit, 2424 x 2424 pixel resolution)<br>• TRITC filter cube (Excitation 542/20, Emission 620/52) (only required for Tissue Optimization protocol)<br>• Minimum Capture Resolution 2.18 µm/pixel<br>• Exposure times 100 milli sec-2 sec
* Only required for Visium Spatial Tissue Optimization protocol & Visium Spatial Imaging Test Slide verification	
**Additional Specifications**	
Image Format	Save image in jpeg or tiff format
Computer	Computer with sufficient power to handle large images (0.5-5 GB)
Software	Image stitching software<br>(microscope's software or equivalent, like Image J)

Recommended Thermal Cyclers

Supplier	Description	Part Number
BioRad	C1000 Touch Thermal Cycler with 96-Deep Well Reaction Module	1851197
Eppendorf	MasterCycler Pro	North America 950030010<br>International 6321 000.019
Thermo Fisher Scientific	Veriti 96-Well Thermal Cycler	4375786

Recommended Real Time qPCR Systems

Supplier	Description	Part Number
Applied Biosystems	QuantStudio 12K Flex system	4471087
Bio-Rad	CFX96 Real-time System	1855096

Additional Kits, Reagents & Equipment

The items in the table below have been validated by 10x Genomics and are highly recommended for the Visium Spatial Reagent Kits protocol. Substituting materials may adversely affect system performance. This list does not include standard laboratory equipment such as water baths, centrifuges, vortex mixers, pH meters, freezers etc.

Supplier	Description	Part Number (US)
**Plastics**		
Eppendorf	PCR Tubes 0.2 ml 8-tube strips<br>DNA LoBind Tubes, 1.5 ml<br>DNA LoBind Tubes, 2.0 ml	951010022<br>022431021<br>022431048
Corning	Self-Standing Polypropylene Centrifuge Tubes (50 ml), sterile<br>Corning 250 mL Vacuum System, 0.2 µm Pore 19.6cm^2 NY Membrane	430921<br>430771
USA Scientific	TempAssure PCR 8-tube strip	1402-4700
Bio-Rad	Hard-shell PCR Plates 96-well, thin wall (pkg of 50)<br>(alternatively, use any compatible PCR Plate)<br>Microseal 'B' PCR Plate Sealing Film, adhesive<br>(alternatively, use any PCR Plate sealing adhesive)	HSP9665<br><br>MSB1001
Thermo Fisher Scientific	Simport Scientific LockMailer Tamper Evidence Slide Mailer<br>(alternatively, use a 50-ml centrifuge tube)<br>MicroAmp 8-Tube Strip, 0.2 ml<br>MicroAmp 8 -Cap Strip, clear	22-038-399<br><br>N8010580<br>N8010535
Rainin	Tips LTS 200UL Filter RT-L200FLR<br>Tips LTS 1ML Filter RT-L1000FLR<br>Tips LTS 20UL Filter RT-L10FLR	30389240<br>30389213<br>30389226
VWR	Divided Polystyrene Reservoirs	41428-958
**Kits & Reagents**		
Agilent	Hematoxylin, Mayer's (Lillie's Modification)<br>(alternatively, Mayer's Hematoxylin from Electron Microscopy Services, 2638102 may be used)<br>Bluing Buffer, Dako	S330930-2<br><br>CS70230-2
Thermo Fisher Scientific	Nuclease-free Water<br>Low TE Buffer (10 mM Tris-HCl pH 8.0, 0.1 mM EDTA)<br>Tris Base (White Crystals or Crystalline Powder/Molecular Biology)<br>Tris 1M, pH 7.0, RNase-free	AM9937<br>12090-015<br>BP152-500<br>AM9850G
Fisher Chemical	Hydrochloric Acid Solution, 0.1N	SA54-1
KAPA Biosystems	KAPA SYBR FAST qPCR Master Mix (2X)	KK4600
Millipore Sigma	Ethanol, Pure (200 Proof, anhydrous)<br>Potassium Hydroxide Solution, 8M<br>Methanol, for HPLC, ≥ 99.9%<br>2-Propanol (Isopropanol)<br>20X SSC<br>Eosin Y solution, aqueous, 0.5% (w/v) in water<br>Acetic acid, ≥ 99.9%	E7023-500ML<br>P4494-50ML<br>34860<br>I9516-25ML<br>S6639-1L<br>HT110216-500ML<br>A6283
Beckman Coulter	SPRIselect Reagent Kit	B23318
Qiagen	Qiagen Buffer EB	19086
-	Ultrapure/Milli-Q water (from Milli-Q Integral ultrapure water system or equivalent)	

Additional Kits, Reagents & Equipment

The items in the table below have been validated by 10x Genomics and are highly recommended for the Visium Spatial Reagent Kits protocol. Substituting materials may adversely affect system performance. This list does not include standard laboratory equipment such as water baths, centrifuges, vortex mixers, pH meters, freezers etc.

Supplier	Description	Part Number (US)
**Equipment**		
Labnet	Slide Spinner<br>(alternatively, use a 50-ml centrifuge tube in a centrifuge with a swing-bucket rotor)	C1303-T
Rainin	Pipet-Lite Multi Pipette L8-200XLS+<br>Pipet-Lite LTS Pipette L-2XLS+<br>Pipet-Lite LTS Pipette L-10XLS+<br>Pipet-Lite LTS Pipette L-20XLS+<br>Pipet-Lite LTS Pipette L-100XLS+<br>Pipet-Lite LTS Pipette L-200XLS+<br>Pipet-Lite LTS Pipette L-1000XLS+	17013805<br>17014393<br>17014388<br>17014392<br>17014384<br>17014391<br>17014382
**Quantification & Quality Control**		
Agilent	2100 Bioanalyzer Laptop Bundle<br>High Sensitivity DNA Kit<br>4200 TapeStation<br>High Sensitivity D1000 ScreenTape/Reagents<br>High Sensitivity D5000 ScreenTape/Reagents	G2943CA<br>5067-4626<br>G2991AA<br>5067-5592/ 5067-5593<br>5067-5584/ 5067-5585
PerkinElmer	LabChip GX Touch HT Nucleic Acid Analyzer<br>DNA High Sensitivity Reagent Kit	CLS137031<br>CLS760672
Advanced Analytical	Fragment Analyzer Automated CE System - 12 cap<br>Fragment Analyzer Automated CE System - 48/96 cap<br>High Sensitivity NGS Fragment Analysis Kit	FSv2-CE2F<br>FSv2-CE10F<br>DNF-474
KAPA Biosystems	KAPA Library Quantification Kit for Illumina Platforms	KK4824

Choose Bioanalyzer, TapeStation, Lab Chip or Fragment Analyzer based on availability & preference.
