# Supplementary material for: MIF promotes cell invasion by the LRP1-uPAR interaction in pancreatic cancer cells
Source: Front Oncol. 2023 Jan 10;12:1028070. doi: 10.3389/fonc.2022.1028070 (PMC9871987; doi:10.3389/fonc.2022.1028070)
Supplement: Supplementary file 5 [file DataSheet_5.pdf]

1. Flow chart of Experiments

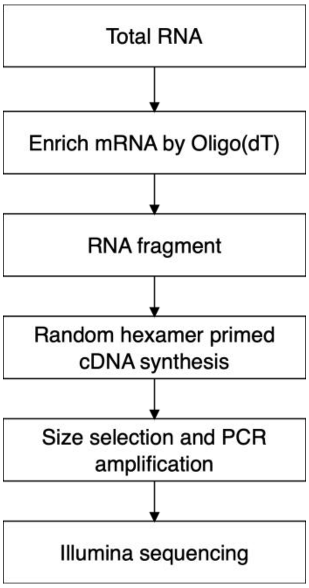

2. RNA extraction library construction and sequencing

Total RNA was extracted using Trizol reagent (thermofisher, 15596018) following the manufacturer's procedure. The total RNA quantity and purity were analysis of Bioanalyzer 2100 and RNA 6000 Nano LabChip Kit (Agilent, CA, USA, 5067-1511) , high-quality RNA samples with RIN number > 7.0 were used to construct sequencing library. After total RNA was extracted, mRNA was purified from total RNA (5ug) using Dynabeads Oligo (dT) (Thermo Fisher, CA, USA) with two rounds of purification. Following purification, the mRNA was fragmented into short fragments using divalent cations under elevated temperature (Magnesium RNA Fragmentation Module (NEB, cat.e6150, USA) under 94℃ 5-7min). Then the cleaved RNA fragments were reverse-transcribed to create the cDNA by SuperScript™ II Reverse Transcriptase (Invitrogen, cat. 1896649, USA), which were next used to synthesise U-labeled second-stranded DNAs with E. coli DNA polymerase I (NEB, cat.m0209, USA), RNase H (NEB, cat.m0297, USA) and dUTP Solution (Thermo Fisher, cat.R0133, USA). An A-base was then added to the blunt ends of each strand, preparing them for ligation to the indexed adapters. Each adapter contained a T-base overhang for ligating the adapter to the A-tailed fragmented DNA. Dual-index adapters were ligated to the fragments, and size selection was performed with AMPureXP beads. After the heat-labile UDG enzyme (NEB, cat.m0280, USA) treatment of the U-labeled second-stranded DNAs, the ligated products were amplified with PCR by the following conditions: initial denaturation at 95℃ for 3 min; 8 cycles of denaturation at 98℃ for 15 sec, annealing at 60℃ for 15 sec, and extension at 72℃ for 30 sec; and then final extension at 72℃ for 5 min. The average insert size for the final cDNA librarys were 300±50 bp. At last, we performed the 2×150bp paired-end sequencing (PE150) on an Illumina Novaseq™ 6000 (LC-Bio Technology CO., Ltd., Hangzhou, China) following the vendor's recommended protocol.

3. Bioinformatics Analysis

3.1 Sequence and filtering of Clean Reads

A cDNA library constructed by technology from the pooled RNA from < **sample description** > samples of < **research species** > was sequenced run with Illumina Novaseq™ 6000 sequence platform. Using the Illumina paired-end RNA-seq approach, we sequenced the transcriptome, generating a total of millon 2 x 150 bp paired-end reads. Reads obtained from the sequencing machines includes raw reads containing adapters or low quality bases which will affect the following assembly and analysis. Thus, to get high quality clean reads, reads were further filtered by Cutadapt (https://cutadapt.readthedocs.io/en/stable/, version:cutadapt-1.9). The parameters were as follows:

1) removing reads containing adapters;
2) removing reads containing polyA and polyG;
3) removing reads containing more than 5% of unknown nucleotides (N);
4) removing low quality reads containing more than 20% of low quality (Q-value≤20) bases.

Then sequence quality was verified using FastQC (http://www.bioinformatics.babraham.ac.uk/projects/fastqc/, 0.11.9). including the Q20, Q30 and GC-content of the clean data.

After that, a total of G bp of cleaned, paired-end reads were produced. The raw sequence data have been submitted to the NCBI Gene Expression Omnibus (GEO) datasets with accession number < **GEO accession** >
or NCBI Short Read Archive (SRA) with accession number with accession number < **SRA accession** >.

References:

MARTIN, Marcel. Cutadapt removes adapter sequences from high-throughput sequencing reads. EMBnet.journal, [S.l.], v. 17, n. 1, p. pp. 10-12, may 2011. ISSN 2226-6089.

Thompson O, von Meyenn F, Hewitt Z, Alexander J, Wood A, Weightman R, Gregory S, Krueger F, Andrews S, Barbaric I, Gokhale PJ, Moore HD, Reik W, Milo M, Nik-Zainal S, Yusa K, Andrews PW. Low rates of mutation in clinical grade human pluripotent stem cells under different culture conditions. Nat Commun.2020 Mar 23;11(1):1528. doi: 10.1038/s41467-020-15271-3(FastQC)

3.2 Alignment with Reference Genome

We aligned reads of all samples to the < **research species** > reference genome using HISAT2 (https://daehwankimlab.github.io/hisat2/, version:hisat2-2.0.4) package, which initially remove a portion of the reads based on quality information accompanying each read and then maps the reads to the reference genome. HISAT2 allows multiple alignments per read (up to 20 by default) and a maximum of two mismatch when mapping the reads to the reference. HISAT2 build a database of potential splice junctions and confirms these by comparing the previously unmapped reads against the database of putative junctions.

References:

Kim, D., Paggi, J.M., Park, C. et al. Graph-based genome alignment and genotyping with HISAT2 and

HISAT-genotype. Nat Biotechnol 37, 907–915 (2019).
Kim D, Langmead B and Salzberg SL. HISAT: a fast spliced aligner with low memory requirements. Nature Methods 2015.
Pertea M, Kim D, Pertea G, Leek JT and Salzberg SL. Transcript-level expression analysis of RNA-seq experiments with HISAT, StringTie and Ballgown. Nature Protocols 2016.

3.3 Quantification of Gene Abundance

The mapped reads of each sample were assembled using StringTie (http://ccb.jhu.edu/software/stringtie/, version:stringtie-1.3.4d) with default parameters. Then, all transcriptomes from all samples were merged to reconstruct a comprehensive transcriptome using gffcompare software (http://ccb.jhu.edu/software/stringtie/gffcompare.shtml, version:gffcompare-0.9.8). After the final transcriptome was generated, StringTie and ballgown (http://www.bioconductor.org/packages/release/bioc/html/ballgown.html) were used to estimate the expression levels of all transcripts and perform expression abundance for mRNAs by calculating FPKM (fragment per kilobase of transcript per million mapped reads) value.

References:

Pertea M, Kim D, Pertea G, Leek JT and Salzberg SL. Transcript-level expression analysis of RNA-seq experiments with HISAT, StringTie and Ballgown. Nature Protocols 2016.
Kovaka S, Zimin AV, Pertea GM, Razaghi R, Salzberg SL, Pertea M Transcriptome assembly from long-read RNA-seq alignments with StringTie2, Genome Biology 20, 278 (2019), doi:10.1186/s13059-019-1910-1.
Pertea M, Pertea GM, Antonescu CM, Chang TC, Mendell JT & Salzberg SL. StringTie enables improved reconstruction of a transcriptome from RNA-seq reads Nature Biotechnology 2015, doi:10.1038/nbt.3122.

3.4 Differentially expressed genes (DEGs) Analysis

Genes differential expression analysis was performed by DESeq2 software between two different groups (and by edgeR between two samples). The genes with the parameter of false discovery rate (FDR) below 0.05 and absolute fold change ≥ 2 were considered differentially expressed genes. Differentially expressed genes were then subjected to enrichment analysis of GO functions and KEGG pathways.

References:

Sahraeian SME, Mohiyuddin M, Sebra R, et al. Gaining comprehensive biological insight into the transcriptome by performing a broad-spectrum RNA-seq analysis. Nat Commun. 2017 Jul 5;8(1):59.
Love MI, Huber W, Anders S. Moderated estimation of fold change and dispersion for RNA-seq data with DESeq2. Genome Biol. 2014;15(12):550.
Robinson MD, McCarthy DJ, Smyth GK. edgeR: a Bioconductor package for differential expression analysis of digital gene expression data. Bioinformatics. 2010 Jan 1;26(1):139-40.

Benjamini, Y., & Hochberg, Y. (1995). Controlling the False Discovery Rate: A Practical and Powerful Approach to Multiple Testing. Journal of the Royal Statistical Society. Series B (Methodological), 57(1), 289-300. Retrieved May 30, 2021.

3.5 Relationship analysis of samples

3.5.1 Correlation Analysis of Replicas

We use R to perform correlation analysis. Correlation of two parallel experiments provides the evaluation of the reliability of experimental results as well as operational stability. The pearson correlation coefficient between two replicas was calculated to evaluate repeatability between samples. The closer the correlation coefficient gets to 1, the better the repeatability between two parallel experiments.

3.5.2 Principal Component Analysis

Principal component analysis (PCA) was performed with princomp function of R (http://www.r- project.org/) in this experience. PCA is a statistical procedure that converts hundreds of thousands of correlated variables (gene expression) into a set of values of linearly uncorrelated variables called principal components. PCA is largely used to reveal the structure/relationship of the samples/datas.

3.6 GO Enrichment Analysis

Gene Ontology (GO) is an international standardized gene functional classification system which offers a dynamic-updated controlled vocabulary and a strictly defined concept to comprehensively describe properties of genes and their products in any organism. GO has three ontologies: molecular function, cellular component and biological process. The basic unit of GO is GO-term. Each GO-term belongs to a type of ontology.

GO enrichment analysis provides all GO terms that significantly enriched in DEGs comparing to the genome background. Firstly all DEGs were mapped to GO terms in the Gene Ontology database (http://www.geneontology.org/), gene numbers were calculated for every term, significantly enriched GO terms in DEGs comparing to the genome background were defined by hypergeometric test. The calculating formula of P-value is:

$$P = 1 - \sum_{i=0}^{m-1} \frac{\binom{M}{i}\binom{N-M}{n-i}}{\binom{N}{n}}$$

Here N is the number of all genes with GO annotation; n is the number of DEGs in N; M is the number of all genes that are annotated to the certain GO terms; m is the number of DEGs in M.

N stands for Total background gene (TB gene number); n stands for Total significant gene (TS gene number); M

stands for Background gene (B gene number); m stands for Significant gene (S gene number).
GO terms meeting this condition with $p < 0.05$ were defined as significantly enriched GO terms in DEGs. This analysis was able to recognize the main biological functions that DEGs exercise.

References:

http://geneontology.org

Ashburner M, Ball CA, Blake JA, Botstein D, Butler H, Cherry JM, Davis AP, Dolinski K, Dwight SS, Eppig JT, Harris MA, Hill DP, Issel-Tarver L, Kasarskis A, Lewis S, Matese JC, Richardson JE, Ringwald M, Rubin GM, Sherlock G. Gene ontology: tool for the unification of biology. The Gene Ontology Consortium. Nat Genet. 2000 May;25(1):25-9. doi: 10.1038/75556. PMID: 10802651; PMCID: PMC3037419.

Gene Ontology Consortium. The Gene Ontology resource: enriching a GOld mine. Nucleic Acids Res. 2021 Jan 8;49(D1):D325-D334. doi: 10.1093/nar/gkaa1113. PMID: 33290552; PMCID: PMC7779012.

3.7 Pathway Enrichment Analysis (KEGG)

Genes usually interact with each other to play roles in certain biological functions. Pathway-based analysis helps to further understand genes biological functions. KEGG is the major public pathway-related database. Pathway enrichment analysis identified significantly enriched metabolic pathways or signal transduction pathways in DEGs comparing with the whole genome background. The calculating formula of P-value is:

$$P = 1 - \sum_{i=0}^{m-1} \frac{\binom{M}{i}\binom{N-M}{n-i}}{\binom{N}{n}}$$

Here N is the number of all genes that with KEGG annotation, n is the number of DEGs in N, M is the number of all genes annotated to specific pathways, and m is number of DEGs in M. Pathways meeting this condition with $p < 0.05$ were defined as significantly enriched pathways in DEGs.
N stands for Total background gene (TB gene number); n stands for Total significant gene (TS gene number); M stands for Background gene (B gene number); m stands for Significant gene (S gene number).

References:

https://www.kegg.jp/kegg/

Kanehisa M, Furumichi M, Sato Y, Ishiguro-Watanabe M, Tanabe M. KEGG: integrating viruses and cellular organisms. Nucleic Acids Res. 2021 Jan 8;49(D1):D545-D551. doi: 10.1093/nar/gkaa970. PMID: 33125081; PMCID: PMC7779016.

3.8 Gene Set Enrichment Analysis (GSEA)

We performed gene set enrichment analysis using software GSEA (v4.1.0) and MSigDB to identify whether a set of genes in specific GO terms, KEGG pathways, DO terms (for Homo sapiens), Reactome (for a few model animals) shows significant differences in two groups. Briefly, we input gene expression matrix and rank genes by Signal2Noise normalization method. Enrichment scores and p value was calculated in default parameters. GO terms, KEGG pathways (DO terms, Reactome) meeting this condition with |NES|>1, NOM p-val<0.05, FDR q-val<0.25 were considered to be different in two groups.

References:

Subramanian A, Tamayo P, Mootha VK, Mukherjee S, Ebert BL, Gillette MA, Paulovich A, Pomeroy SL, Golub TR, Lander ES, Mesirov JP. Gene set enrichment analysis: a knowledge-based approach for interpreting genome-wide expression profiles. Proc Natl Acad Sci U S A. 2005 Oct 25;102(43):15545-50. doi: 10.1073/pnas.0506580102. Epub 2005 Sep 30. PMID: 16199517; PMCID: PMC1239896.

Mootha VK, Lindgren CM, Eriksson KF, Subramanian A, Sihag S, Lehar J, Puigserver P, Carlsson E, Ridderstråle M, Laurila E, Houstis N, Daly MJ, Patterson N, Mesirov JP, Golub TR, Tamayo P, Spiegelman B, Lander ES, Hirschhorn JN, Altshuler D, Groop LC. PGC-1alpha-responsive genes involved in oxidative phosphorylation are coordinately downregulated in human diabetes. Nat Genet. 2003 Jul;34(3):267-73. doi: 10.1038/ng1180. PMID: 12808457.

3.9 Alternative Splicing Analysis

rMATS (version 4.1.1) (http://rnaseq-mats.sourceforge.net) was used to identify alternative splicing events and analyze differential alternative splicing events between samples. We identified AS events with a false discovery rate (FDR) < 0.05 in a comparison as significant AS events.

The classification of alternative splicing is as follows:

SE: skipped exon

MXE: mutually exclusive exon

A5SS: alternative 5’ splice site

A3SS: alternative 3’ splice site

RI: retained intron

References:

Shen S., Park JW., Lu ZX., Lin L., Henry MD., Wu YN., Zhou Q., Xing Y. rMATS: Robust and Flexible Detection of Differential Alternative Splicing from Replicate RNA-Seq Data. PNAS, 111(51):E5593-601. doi: 10.1073/pnas.1419161111.

Park JW., Tokheim C., Shen S., Xing Y. Identifying differential alternative splicing events from RNA sequencing data using RNASeq-MATS. Methods in Molecular Biology: Deep Sequencing Data Analysis, 2013;1038:171-179 doi: 10.1007/978-1-62703-514-9_10.

Shen S., Park JW., Huang J., Dittmar KA., Lu ZX., Zhou Q., Carstens RP., Xing Y. MATS: A Bayesian Framework

for Flexible Detection of Differential Alternative Splicing from RNA-Seq Data. Nucleic Acids Research, 2012;40(8):e61 doi: 10.1093/nar/gkr1291.

3.10 Single-nucleotide Polymorphism (SNP) Analysis

The Samtools (0.1.19) was used for calling variants of transcripts, and ANNOVAR was used for SNP/InDel annotation. The function, genome site and type of variation of SNPs were also analyzed.

References:

Danecek P, Bonfield JK, Liddle J, Marshall J, Ohan V, Pollard MO, Whitwham A, Keane T, McCarthy SA, Davies RM, Li H. Twelve years of SAMtools and BCFtools. Gigascience. 2021 Feb 16;10(2):giab008. doi: 10.1093/gigascience/giab008. PMID: 33590861; PMCID: PMC7931819.

Li H. A statistical framework for SNP calling, mutation discovery, association mapping and population genetical parameter estimation from sequencing data. Bioinformatics. 2011 Nov 1;27(21):2987-93. doi: 10.1093/bioinformatics/btr509. Epub 2011 Sep 8. PMID: 21903627; PMCID: PMC3198575.

Wang K, Li M, Hakonarson H. ANNOVAR: functional annotation of genetic variants from high-throughput sequencing data. Nucleic Acids Res. 2010 Sep;38(16):e164. doi: 10.1093/nar/gkq603. Epub 2010 Jul 3. PMID: 20601685; PMCID: PMC2938201.
