# Supplementary material for: MIF promotes cell invasion by the LRP1-uPAR interaction in pancreatic cancer cells
Source: Front Oncol. 2023 Jan 10;12:1028070. doi: 10.3389/fonc.2022.1028070 (PMC9871987; doi:10.3389/fonc.2022.1028070)
Supplement: Supplementary file 6 [file Image_1.pdf]

Supplementary Material

Supplementary Figures

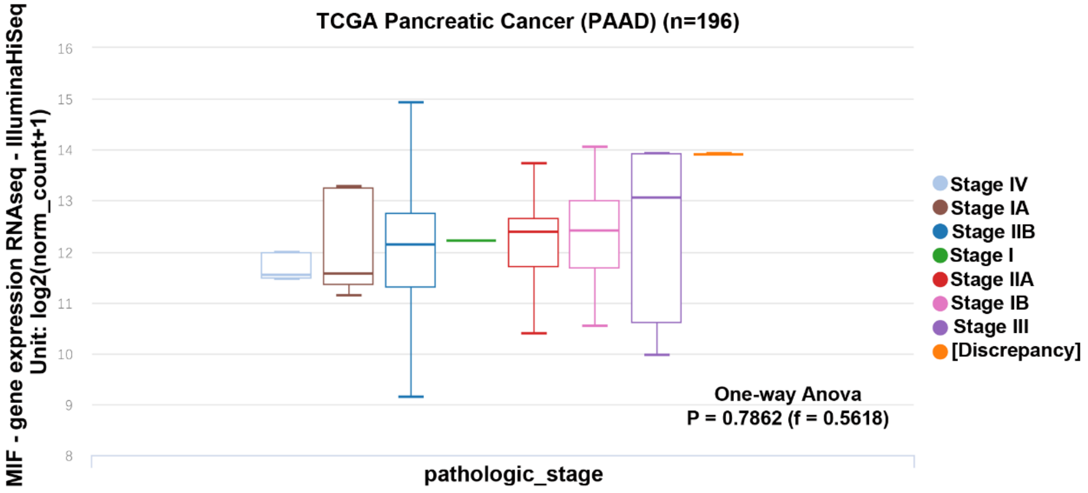

Figure S 1. Correlation of MIF expression with the stages of pancreatic cancer.

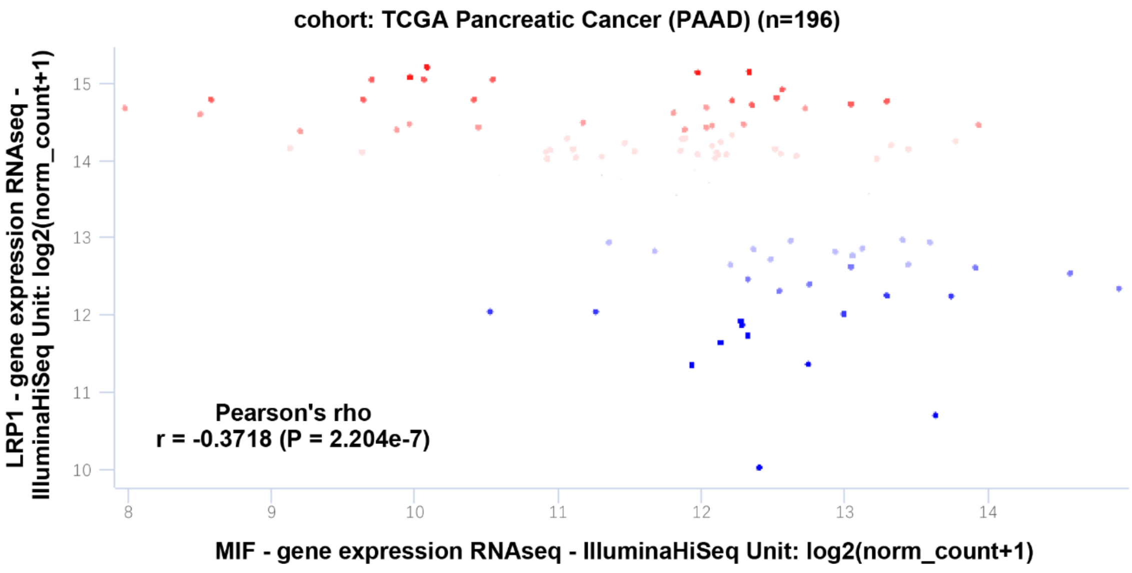

Figure S 2. Correlation of MIF and LRP1 levels in 196 pancreatic cancer samples.

The negative correlation between MIF (x axis) and IRP1 (y axis) in the TCGA-pancreatic cancer (PAAD) patients analyzed from the UCSC Xena database. Pearson's correlation coefficient (rho) r = -0.3718 (P = 2.204e-7).

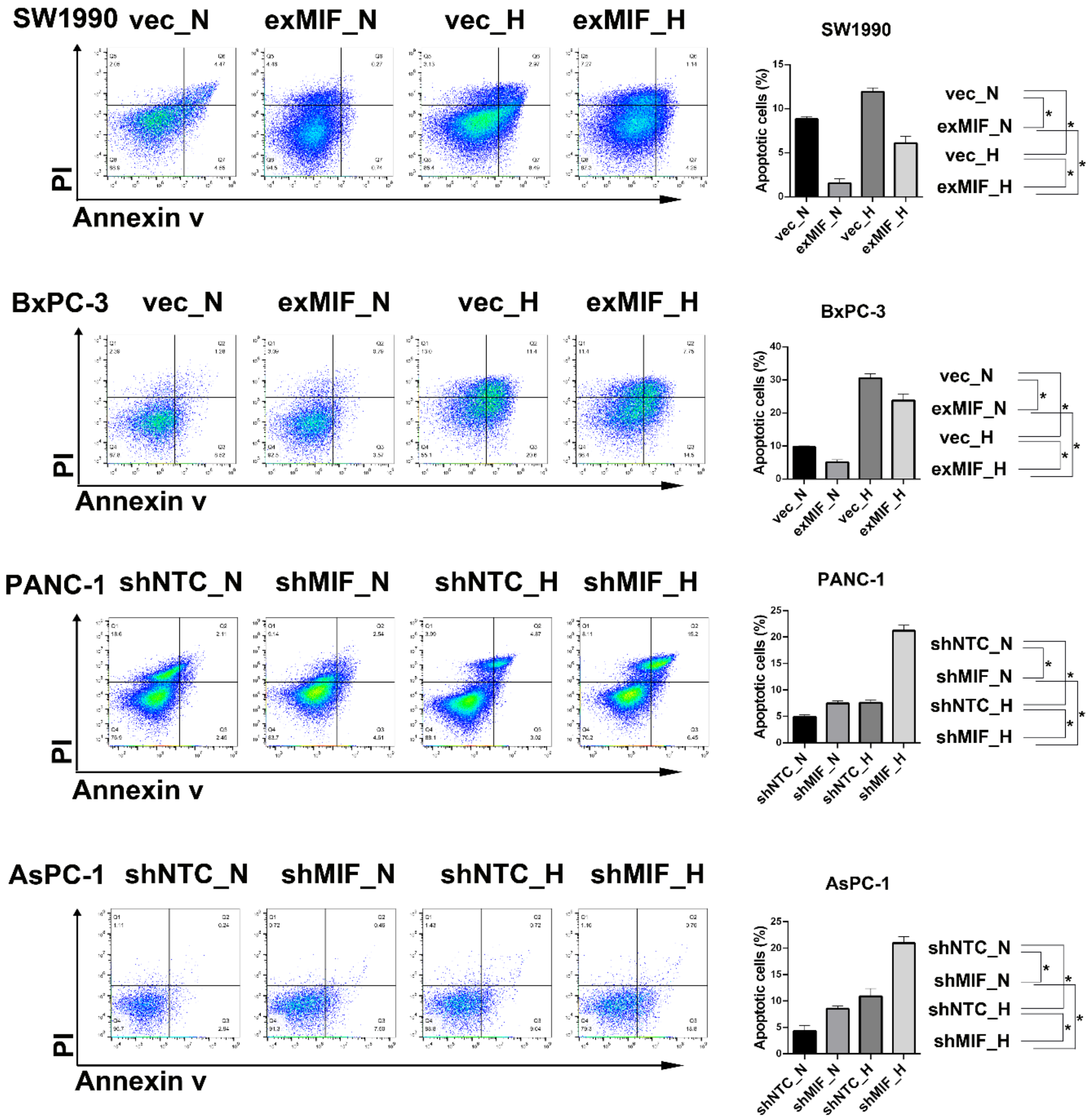

Figure S 3. MIF knockdown promotes apoptosis of PDAC cells.

Representative images and quantification of apoptotic cells measured using the An-nexin V-APC/PI by flow cytometry after $CoCl_2$ (H, hypoxia) treatment or vehicle (N, normoxia) for 24h. *$P < 0.05$.
